# Photodegradable branched polyethylenes from carbon monoxide copolymerization under benign conditions

**DOI:** 10.1038/s41467-020-17542-5

**Published:** 2020-07-23

**Authors:** Tobias O. Morgen, Maximilian Baur, Inigo Göttker-Schnetmann, Stefan Mecking

**Affiliations:** Chair of Chemical Materials Science, Department of Chemistry, University of Konstanz, 78457 Konstanz, Germany

**Keywords:** Pollution remediation, Green chemistry, Polymer characterization, Polymer synthesis, Polymerization mechanisms

## Abstract

Small amounts of in-chain keto groups render polyethylene (PE) photodegradable, a desirable feature in view of environmental plastics pollution. Free-radical copolymerization of CO and ethylene is challenging due to the formation of stable acyl radicals which hinders further chain growth. Here, we report that copolymerization to polyethylenes with desirable low ketone content is enabled in dimethyl carbonate organic solvent or under aqueous conditions at comparatively moderate pressures <350 atm that compare favorable to typical ethylene polymerization at 2000 atm. Hereby, thermoplastic processable materials can be obtained as demonstrated by injection molding and tensile testing. Colloidally stable dipersions from aqueous polymerizations form continuous thin films upon drying at ambient conditions. Extensive spectroscopic investigation including ^13^C labeling provides an understanding of the branching microstructures associated with keto groups. Exposure of injection molded materials or thin films to simulated sunlight under sea-like conditions results in photodegradation.

## Abstract (web summary)

Small amounts of in-chain keto groups render polyethylene (PE) photodegradable, a desirable feature in view of environmental plastics pollution. Here the authors introduce low ketone content by copolymerization in organic solvent or under aqueous conditions at moderate pressures.

## Introduction

Polyethylenes (PEs) are the largest scale synthetic polymers, fulfilling a myriad of essential functions in modern technologies^1^. This versatility arises from the ability to vary mechanical properties via the branching structure and resulting crystallinity, together with the chemically inert and hydrophobic nature of polyethylene. Thus applications of PEs span from high performance structural applications over strong films to aqueous-based coatings^2^.

Due to their extensive use, PEs also account for a substantial share of environmental plastic waste pollution^3^. Their unreactive hydrocarbon chains resist breakdown, and can persist for many years. In general terms, across different types of plastics, hydrolytic breakdown is the most common approach to degrade plastics in the environment^4^. This is extremely slow under marine conditions^5^. A powerful alternative is photodegradation. In-chain keto groups are particularly well suited to promote photodegradation, as they enable chain scission by Norrish type I and II reactions^6^. While this principle is recognized for polyolefins^7,8^, it has found little attention due to the challenges associated with synthesizing polyolefins with in-chain keto groups.

PEs are produced by catalytic processes, as well as high pressure free-radical polymerization at typically 200 °C and 2000 atm^9,10^. Rather undefined keto-, ester-, and carboxyl-modified PEs can be obtained by an additional partial oxidation step after polymerization^11,12^. In both free-radical and catalytic insertion chain growth of ethylene, PEs with in-chain keto groups can be generated in only one step by incorporation of carbon monoxide comonomer^13,14^. Albeit these reactions are entirely different mechanistically, they are both hampered by CO. In catalytic chain growth, free CO and the formed keto groups strongly block coordination sites for further chain growth. Also, the high reactivity of CO results in the formation of alternating polyketones rather than the desired PEs with a low keto group density^15,16^. Therefore, other catalytic approaches using special comonomers to incorporate isolated keto groups into PE were investigated more recently^17^. In free-radical growth, the low reactivity of the acyl radicals formed upon incorporation of CO hinders further chain growth and favors the formation of oligomers rather than PE materials^18^.

We now report that PEs with a desirable concentration of in-chain keto groups (e.g., 1%) can be generated at comparatively moderate pressures of <350 atm. Key is the choice solvent, combined with an appropriate relative monomer concentration. Thermoplastic processable material is obtained. Aqueous polymerizations yield dispersions of keto-substituted branched PEs amenable to film formation.

## Results

### Polymerizations in organic solution

Commercial free-radical polymerization of ethylene commonly employs supercritical ethylene as a solvent, which requires high pressures and temperatures (>1000 atm). The presence of organic solvents usually results in chain transfer reactions to the solvent which deteriorate molecular weight^13,19^. Recently, dimethyl carbonate has been found to be uniquely inert concerning chain transfer to solvent in studies of ethylene homopolymerization by Monteil and coworkers^19^.

**Fig. 1:**
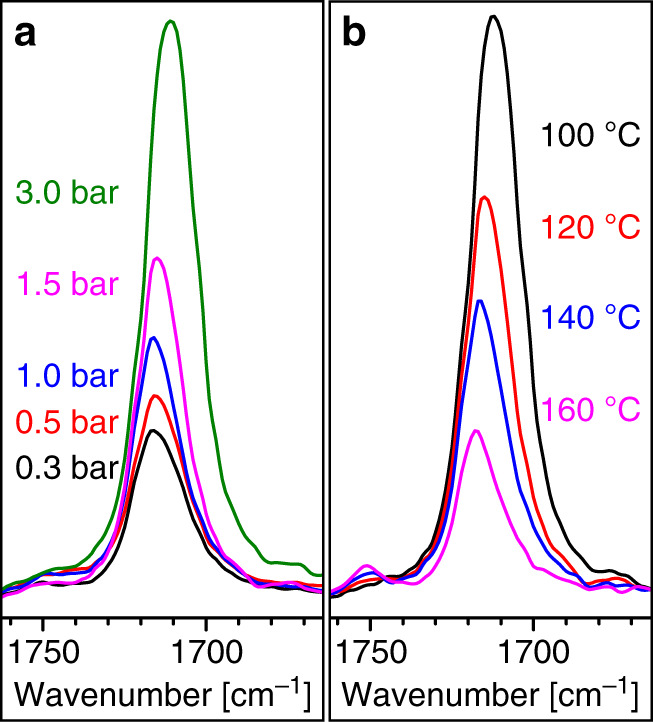
IR spectra of polyketones. Signals of the keto group in ATR-IR spectra of samples synthesized at (**a**) different initial CO pressures (entries 2–4, 6, and 7 in Table 1) and (**b**) temperatures (entries 6, 8–10 in Table 1).

Ethylene and CO were therefore copolymerized in dimethyl carbonate (DMC) in addition to more common solvents tetrahydrofuran (THF), dichloromethane (DCM), and ethyl acetate (EA). Polymerization at 300 bar and 100–160 °C at low partial pressures of CO afforded PEs with ketone contents of 1.5–13% CO (Table 1. For details of polymerization procedures and polymer analysis *cf*. Supplementary Methods and Supplementary Figs. 1–18 in the Supplementary Information. Note that due to the virtually identical molecular weights of ethylene and CO, molar and weight incorporations are numerically identical). As expected, the ketone content correlates with the CO partial pressure, and is lower at higher polymerization temperatures (Fig. 1. *Vide infra* for a detailed discussion of factors determining copolymer composition). Polymerizations at higher temperatures yield more copolymer but with decreased molecular weight.

**Table 1:** Free-radical copolymerization of ethylene and carbon monoxide in different organic solvents^a^.

#	*T* (°C)	Solvent	Initial *p*(CO) at 20 °C (bar)	Yield (g)	Peak *T*_m_^b^ (°C)	Cryst.^b^ (%)	*M*_w_^c^ (10^3^ g × mol^−1^)	*M*_w_/*M*_n_^c^	*χ*^d^ (mol-%)
1	120	DMC	–	0.92	114	43	25.2	2.0	–
2	120	DMC	0.3	0.66	112	37	22.9	1.7	2.6
3	120	DMC	0.5	0.46	110	36	18.9	1.7	3.6
4	120	DMC	1.0	0.31	108	35	16.5	1.6	4.0
5^e^	120	DMC	1.5	0.23	106	42	13.2	2.0	5.4 (5.6)
6	120	DMC	1.5	0.40	105	34	16.0	1.5	5.9 (5.0)
7	120	DMC	3.0	0.41	99	28	13.7	1.6	13.1 (13.2)
8	100	DMC	1.5	0.09	112	34	19.6	1.7	9.8
9	140	DMC	1.5	2.43	106	32	12.3	1.6	3.9
10	160	DMC	1.5	7.42	103	34	10.2	1.7	2.1
11	120	THF	–	4.31	113	55	1.21	1.3	–
12	120	THF	0.5	2.24	107	45	1.78	1.7	1.5
13	120	THF	1.5	1.29	107	41	1.18	1.3	5.2 (4.1)
14	120	THF	3.1	1.18	101	36	1.17	1.3	10.5 (9.0)
15	100	THF	1.5	0.25	104	40	1.20	1.3	7.4
16	140	THF	1.5	8.50	104	39	1.10	1.3	1.9
17	120	DCM	–	5.10	107	49	<1.0	–	–
18	120	DCM	1.5	1.33	101	39	<1.0	–	5.0 (3.8)
19	120	EA	–	1.52	114	45	9.33	1.9	–
20	120	EA	1.5	0.37	105	35	7.31	1.6	6.0 (4.8)

^a^Reaction conditions: 1 h reaction time, 4 mM di-*tert*-butyl peroxide initiator, 75 mL of solvent, initial ethylene pressure 300–330 bar, 1000 rpm stirring rate (pitched blade).

^b^Determined from 2nd heating cycle of DSC on the isolated bulk polymer.

^c^Determined by GPC at 160 °C calibrated with linear PE.

^d^Carbon monoxide incorporation from ATR-IR (calibrated with a polyketone reference). In brackets: according to ^13^C NMR.

^e^Reaction with ^13^CO instead of CO.

**Fig. 2:**
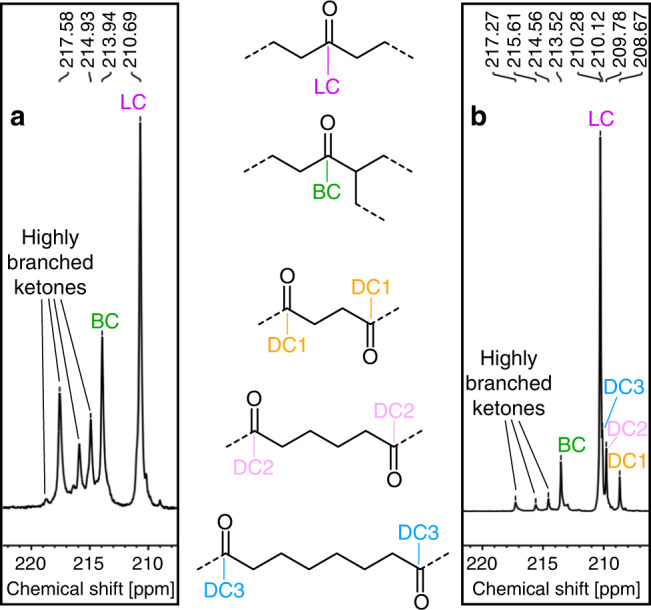
^13^C NMR spectra of polyketones. Resonances of different keto groups in 1,1,2,2-tetrachloroethane-*d*_*2*_ of a polyketone synthesized in (**a**) water (entry 10 in Table 2) and a polyketone snythesized in (**b**) DMC (entry 5 in Table 1). LC: linear carbonyl, BC: α-branched carbonyl, DC: double carbonyls.

To unravel the microstructures of the keto-modified PEs formed, polymers were also prepared using ^13^CO as the comonomer to enable ^13^C NMR analysis. The keto units are largely isolated, with a minor portion in close proximity to one another. The relative amount of alternating to non-alternating CO-ethylene-motives agrees well with expectations for a random copolymerization model (note that consecutive CO incorporations do not occur for thermodynamic reasons). Both NMR and ATR-IR analyses exclude the presence of strictly alternating polyketone segments even in samples with CO contents >10%. That is, in contrast to catalytic CO-ethylene copolymerization, there is indeed no inherent tendency for formation of alternating motives in the free-radical copolymerization studied here. Besides linear ketones, the obtained polymers contain mostly ketones with branches in α-position to the carbonyl (Fig. 2). These groups can originate from either intramolecular chain transfer to the α-position of an in-chain ketone (backbiting^20^) followed by chain propagation or from intramolecular chain transfer to a PE segment followed by incorporation of CO and subsequent ethylene. As concluded from the chain transfer constants of aliphatic ketones compared to alkanes, abstraction of H-atoms in α-position to a carbonyl by a primary polyethylenyl radical is faster than for hydrogens in a PE segment^20^.

Besides mono-branched ketones significant amounts of ketones with two or three branches are observed. These can be accounted for by multiple abstractions, each followed by chain propagation, of the different α-hydrogens of a given ketone moiety. Reported chain transfer constants of low molecular weight compounds indeed suggest that mono-α-branched ketones undergo faster chain transfer reactions than their linear analogues, which favors the formation of highly branched carbonyls^20^. Complimentary to NMR spectroscopic analysis, the fraction of highly branched ketones in the PEs could also be quantified by ATR-IR spectroscopic band deconvolution (*cf*. Supplementary Figs. 11–13, 37–39) in which these groups evoke an additional band at 1699 cm^−1^.

The overall branching densities are in the range of 8–20 branches per 1000 carbon atoms. Branches tend to cluster directly beside keto groups (e.g., 30% of branches are in α-position to ketones in sample 5 of Table 1, although ketones account for only 5% of repeat units). Small amounts of ketones are located in β- or γ-position to terminal double bonds. It is known that terminal olefins are formed during free-radical polymerization of ethylene when secondary alkyl radicals undergo β-scission^20^. α,β-unsaturated ketones were not observed.

Polyketones synthesized in DCM or THF possess low molecular weights of ca. 1000 g × mol^−1^ and they contain solvent-derived groups. This indicates that transfer reactions of the growing polymer chain with the solvent are the predominant pathway of chain transfer and this dictates polymer molecular weights in the copolymerization in these solvents. EA as a reaction solvent was found to afford significantly higher molecular weights, though not superior to dimethyl carbonate.

**Fig. 3:**
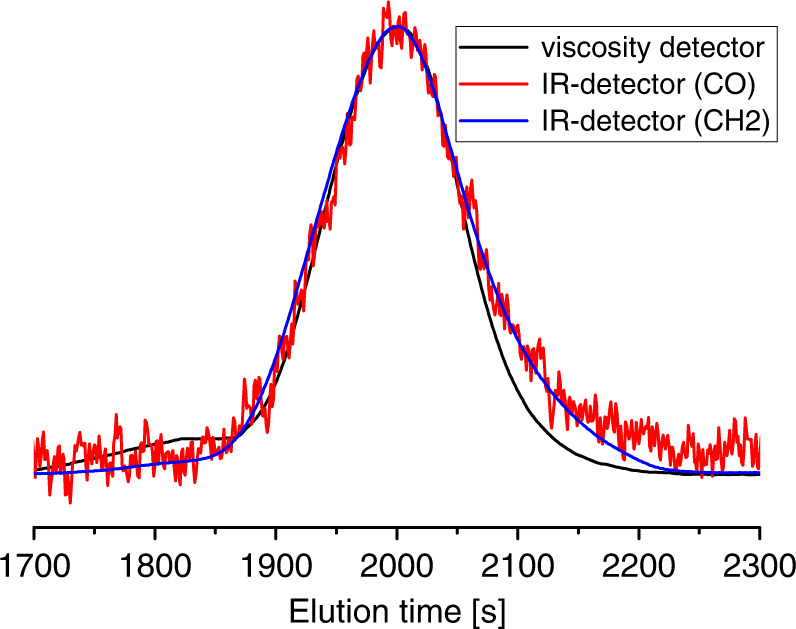
GPC analysis of polyketones. Traces of the viscosity detector (black), C-H sensitive IR detector (blue), and carbonyl-sensitive IR detector (red).

GPC with IR detection at different spectral positions enables an observation of chemical composition resolved by molecular weight. This showed that the polymers formed are homogeneous and well defined with carbonyls equally distributed regardless of molecular weight of the polymer chain (Fig. 3).

The polymers are semicrystalline with crystallinities of 30–55% and peak melting points of 99–114 °C as revealed by DSC analysis. The peak *T*_m_ and the degree of crystallinity decrease somewhat with increasing ketone content. Studies of linear model polymers showed that isolated keto groups can be incorporated into the PE crystal lattice, and this goes along with only a low energy penalty^21^.

### Mechanistic considerations

We used a First-order Markov treatment to determine the relative reactivity of CO to ethylene (*cf*. Supplementary Table 1, Fig. 19).

Since two consecutive CO incorporations do not occur, the CO copolymerization parameter is *r*_CO_ = 0. This simplifies the copolymerization equation, resolved here for the copolymerization parameter of ethylene, to Eq. (1) (*k*_e-E_ = rate constant for addition of ethylene to an alkyl radical and *k*_e-CO_ = rate constant for addition of CO to an alkyl radical, [*E*] = concentration of ethylene in the reaction mixture). Since the conversions of both CO and ethylene are low, [*E*] and [CO] can be approximated by their concentrations in the initial reaction mixture, [*E*]_0_ and [CO]_0_.

1 $$r_E \,=\, \frac{{k_{{\mathrm{e - E}}}}}{{k_{{\mathrm{e - CO}}}}} \,=\, \left\{ {\frac{{{{[E]}}_{{\mathrm{poly}}}}}{{{\mathrm{[CO]}}_{{\mathrm{poly}}}}} \,-\, {\mathrm{1}}} \right\} \,\cdot\, \frac{{\left[ {{\mathrm{CO}}} \right]}}{{\left[ E \right]}} \,\approx\, \left\{ {\frac{{{{[E]}}_{{\mathrm{poly}}}}}{{{\mathrm{[CO]}}_{{\mathrm{poly}}}}} \,-\, {\mathrm{1}}} \right\} \,\cdot\, \frac{{\left[ {{\mathrm{CO}}} \right]_0}}{{\left[ E \right]_0}}.$$

[*E*]_0_ and [CO]_0_ were determined experimentally or calculated by appropriate methods, respectively. From our data, for polymerizations in dimethyl carbonate we determined *r*_E_ = 0.05, 0.11, 0.18, and 0.38 at temperatures of 100, 120, 140, and 160 °C, respectively. The rate constant for the addition of ethylene to an alkyl radical at 83 °C is 4.7 × 10^2^ M^−1^ × s^−1^ ^20^. Literature values for the rate constants of carbonylation of alkyl radicals at 80 °C are in the range of 1.2–6.3 × 10^5^ M^−1^ × s^−1^ ^22^. The ratio of these two rates (at 80 °C) agrees qualitatively with the observed copolymerization parameters.

A comparison with the relative monomer reactivities in catalytic copolymerization is instructive. Data for ethylene-CO copolymerizations by neutral Pd(II) phosphinosulfonato catalysts, which are capable of generating non-alternating motifs at high ethylene/CO ratios in the reaction mixture have been reported by different authors^23–25^. Compared with polyketones from free-radical copolymerization, catalytically synthesized polymers contain more alternating segments even at comparable CO incorporations around 10%. Applying Eq. (1) to the data of Luo et al. (*cf*. Supplementary Tables 2, 3)^24^, we determined *r*_E_ = 0.13 for 90–110 °C. The similarity of this value to the *r*_E_ of the free-radical copolymerization may be interesting from a preparative perspective. Mechanistically, it is clearly coincidental. In both, free-radical and catalytic polymerization, CO is more reactive than ethylene in the immediate incorporation step, that is addition of monomer to an alkyl radical and insertion in a Pd(II) alkyl carbonyl vs. a Pd(II) alkyl olefin complex, respectively. However, in catalytic polymerization the higher affinity for CO vs. ethylene coordination also contributes essentially to its favored incorporation. Further chain growth by addition of ethylene to the resultant acyl radical is comparatively slow, due to the stability of an acyl radical compared to an alkyl radical. This accounts for the lower productivities in radical ethylene-CO copolymerizations compared with ethylene homopolymerization under identical conditions (*cf*. Tables 1, 2). By contrast, insertion of ethylene into the Pd acyl formed in catalytic polymerization is rapid compared with neat polyethylene chain growth (that is insertion in an acyl ethylene complex compared to insertion in the corresponding alkyl ethylene complex). However, this step is hindered by the high binding affinity of CO, forming a dormant acyl carbonyl complex. Furthermore, catalytic copolymerization is hampered by the product of this ethylene insertion forming a stable chelate complex^15,16^.

**Table 2:** Free-radical copolymerization of ethylene and carbon monoxide in water^a^.

#	*T* (°C)	Initial *p*(CO) at 20 °C (bar)	SDS (wt.-%)	Yield (g)	*d*_p_^b^ (nm)	Peak *T*_m_^c^ (°C)	Cryst.^c^ (%)	*M*_w_^d^ (10^3^ g × mol^−1^)	*M*_w_/*M*_n_^d^	*χ*^e^ (mol-%)
1^f^	85	0.7	–	0.42	22 (17)	87	22	7.91	6.0	1.9
2^f^	85	1.1	–	0.29	18 (24)	77	14	8.40	8.6	3.4
3^f^	85	31.4	–	Traces	21	–	–	–	–	15.9
4^f^	95	0.9	–	0.60	20 (33)	82	18	8.32	6.8	2.1
5^f^	115	5.3	–	0.34	87	–	–	–	–	1.3
6^g^	120	–	0.5	17.9^i^	45	86	27	669^j^	>15^j^	–
7^g^	120	0.6	0.5	2.22	14 (28)	86	23	258	11.6	0.6 (0.3)
8^g^	120	1.0	0.5	1.07	7 (23)	72	19	9.17	4.9	1.1
9^g^	120	1.5	0.5	0.87	6 (34)	67	15	6.53	4.2	2.4 (2.3)
10^g, h^	120	1.5	0.5	0.93	6 (21)	73	16	6.78	5.1	1.7 (1.5)
11^g^	120	2.3	0.5	0.59	9 (33)	61	14	5.51	3.3	3.1 (4.8)
12^g^	120	3.0	0.5	0.56	8 (39)	54	10	5.20	3.4	5.4 (7.5)
13^g^	130	3.0	0.5	0.39	10 (46)	53	9	3.37	2.5	2.9
14^g^	135	5.3	0.5	0.39	12	52	5	1.69	1.4	3.5
15^g^	145	0.5	0.5	1.12	12 (13)	69	21	8.74	5.3	0.4

^a^Reaction conditions: 1 h reaction time, 150 mL of water, initial ethylene pressure 260–290 bar, 1000 rpm stirring rate (pitched blade).

^b^Number-average particle diameter determined by DLS. In brackets: particle diameter determined by TEM.

^c^Determined from 2nd heating cycle of DSC on the isolated bulk polymer.

^d^Determined by GPC at 160 °C calibrated with linear PE.

^e^Carbon monoxide incorporation from ATR-IR (calibrated with a polyketone reference). In brackets: according to ^13^C NMR.

^f^Initiated with 2 mM KPS.

^g^Initiated with 2 mM VA-086.

^h^Reaction with ^13^CO instead of CO.

^i^Polymer partially precipitates during reaction.

^j^Bimodal molecular weight distribution with peak molecular weights of 2.3 × 10^4^ and 3.0 × 10^6^ g × mol^−1^.

### Materials properties

Polymers obtained from reactions in DMC or EA could be processed by injection molding to specimens for tensile testing. The materials investigated (entries 9, 10 in Table 1) showed Young’s moduli of 250 and 350 MPa and moderate elongations at break of 10 and 15%. Mechanical properties could be enhanced by terpolymerization of ethylene and CO with small amounts of difunctional ethylene glycol dimethacrylate (EGDMA) or divinyl adipate (DVA) (for details on terpolymer synthesis and characterization *cf*. Supplementary Tables 4, 5, Supplementary Figs. 20–27). These materials displayed elongations at break up to 28% and 50%, respectively, for terpolymers containing ~2% CO and either 1.2 or 0.3 mol-% of EGDMA or DVA.

To gain first indications on their photodegradability in a marine environment specimens immersed in water were exposed to simulated sunlight. After a light exposure corresponding to 16 months of natural sunlight in Southern Europe, a slight but significant weight loss was observed (*cf*. Supplementary Figs. 28, 29). Microscopic observation showed an uneven surface indicative of degradation from the surface and embrittlement. Upon slight mechanical stress the specimens disintegrated.

### Aqueous polymerizations

**Fig. 4:**
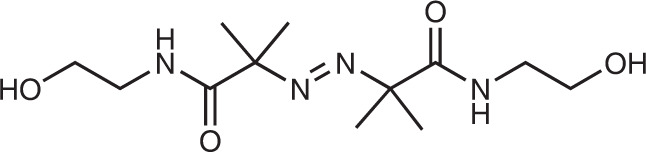
Water-soluble initiator VA-086. Chemical structure of the azo-initiator 2,2′-azobis-[2-methyl-N-(2-hydroxyethyl)-propionamide].

With regard to environmentally friendly and safer polymerization procedures, water is an especially interesting reaction medium. The aqueous dispersion polymerization of ethylene is well studied^26,27^, and besides high shear postpolymerization dispersion preparation from bulk polymer^28^, comprises an efficient route to PE dispersions relevant for e.g., film applications. Copolymerizations of ethylene and CO in water as a reaction medium yield dispersions of keto-modified polyethylene nanoparticles with diameters of ~20 nm (Table 2, *cf*. Supplementary Figs. 30–41). Colloidally stable dispersions were obtained both in a surfactant-free approach^29^ using the anionic initiator potassium persulfate (KPS), and in polymerizations with sodium dodecyl sulfate (SDS) as a stabilizer and the water-soluble azo-initiator VA-086 (Fig. 4).

The particles are mostly spherical, but less uniform in terms of shape and size the higher the CO content, which results in an increasing softness (for details on particle analysis *cf*. Supplementary Figs. 30, 31). Peak melting points are in the range of 50–90 °C and crystallinities range from 5–23%. Since all crystallization temperatures observed on isolated bulk polymers are <80 °C, we conclude that the copolymers do not form semicrystalline particles during the dispersion polymerization but are present as electrostatically stabilized droplets that crystallize only upon work up^29^. Copolymers from these aqueous polymerizations have 27–40 branches per 1000 carbon atoms which is up to fivefold higher vs. polymers from solution polymerization. We ascribe this to the confined volume of growing polymer particles in which the reaction takes place. The higher local polymer concentration favors bimolecular transfer reactions and branch formation.

**Fig. 5:**
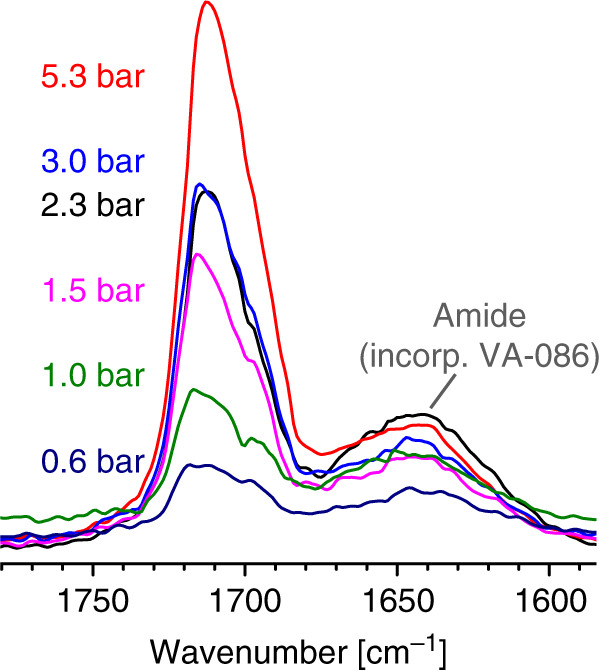
IR spectra of polyketones synthesized in water. Signals of the keto group in ATR-IR spectra of samples synthesized in water at different initial CO pressures (entries 7–9, 11, 12, and 14 in Table 2).

Clustering of branches around keto groups is observed again, with e.g., 50% of branches being in α-position to ketones in the sample of entry 10 in Table 2 (Fig. 2). Besides mono-α-branched ketones, there is a considerable amount of ketones with 2 or 3 branches in α-position indicated by both NMR and ATR-IR spectra (Fig. 5). Again, no alternating polyketone segments were found. According to IR analysis, the spacial distribution of keto groups in the material is similar regardless of the reaction medium (aqueous or organic solvents) and the initiators employed (*cf*. Supplementary Figs. 37–39). Keto moieties appear to be distributed evenly throughout the material, that is there is no indication of any keto-rich and -poor domains.

### Comparison of different polymerization media

**Fig. 6:**
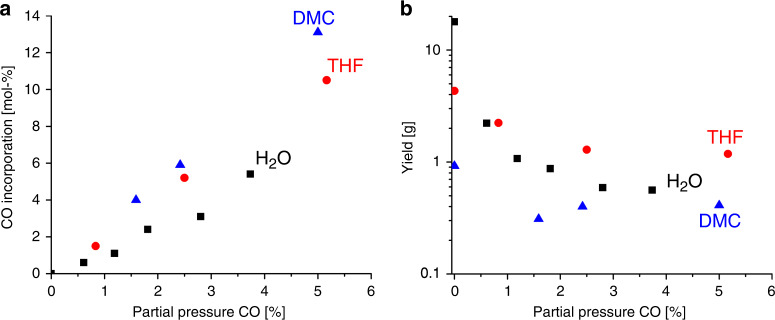
Comparison of different reaction media. Effect of partial CO pressure adjusted at 20 °C on (**a**) the CO incorporation and (**b**) the copolymer yield. Reaction conditions: 120 °C, 260–330 bar total pressure, 1 h, 150 mL of water (black squares) or 75 mL of respective organic solvent (THF: red circles, DMC: blue triangles), 0.3 mmol free-radical initiator (VA-086 in water, di-*tert*-butyl peroxide in organic solvents), 1000 rpm stirring rate with pitched blade stirrer.

The amount of incorporated CO was found to be proportional to the partial pressure of carbon monoxide in the initial monomer mixture (Fig. 6). The incorporation was most efficient in DMC, THF, and water. In all media, the overall copolymer yield decreases with an increasing initial CO partial pressure (Fig. 6), due to the retardation of chain growth by the lower reactivity of acyl radicals (*cf*. mechanistic considerations). The dependence of yield and CO incorporation on the amount of added CO are obviously not affected strongly by the heterogeneous nature of the reaction in water. However, for aqueous ethylene polymerization and copolymerizations with low concentration of CO a high-molecular-weight fraction >10^6^ g × mol^−1^ besides the polymer chains with ~10,000 g × mol^−1^ causes a drastic increase in average *M*_w_ and *M*_w_/*M*_n_. The copolymer yield increases tremendously with the reaction temperature in DMC and THF (*cf*. Supplementary Fig. 43). This was not observed for heterogeneous reactions in water.

Melting points and degrees of crystallinity of isolated bulk polyketones synthesized at identical temperatures of 120 °C are dependent on their CO content and the polymerization medium. We find a pseudo-linear decrease of the peak melting points with increasing CO content for copolymers synthesized in water, DMC, and THF in the composition range investigated (*cf*. Supplementary Fig. 44).

### Keto-functionalized polyethylene films

**Fig. 7:**
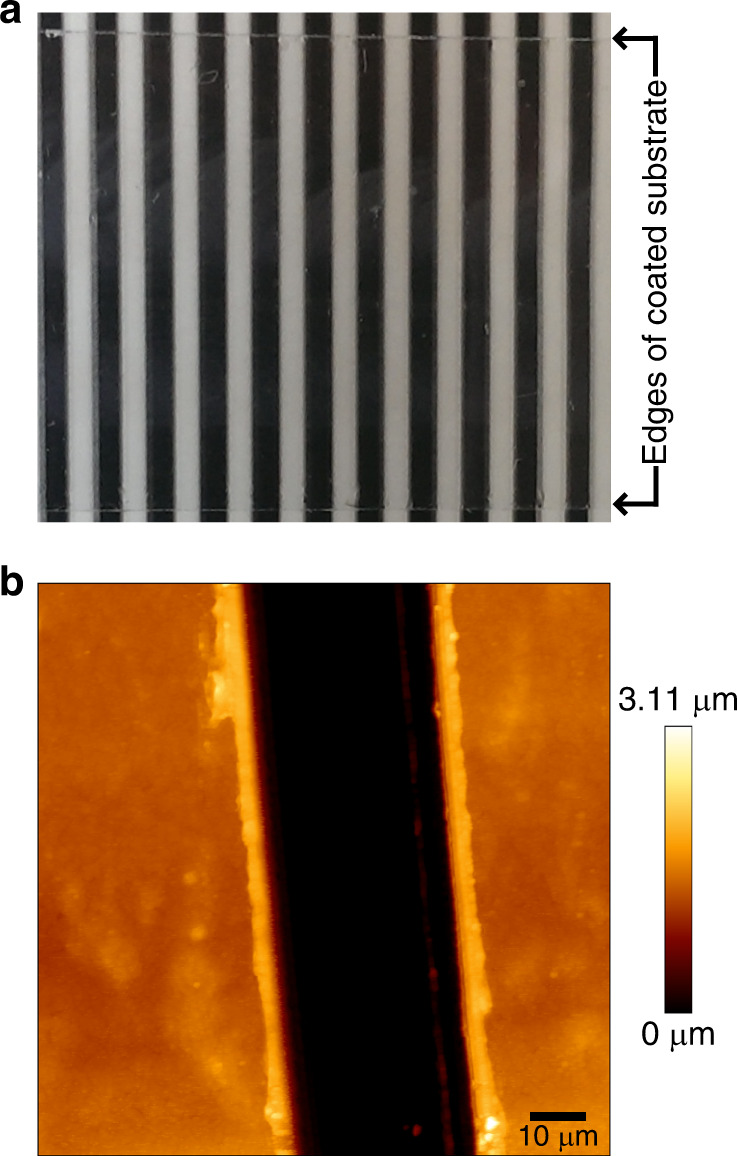
Exemplary polyketone film. Optical appearance of an exemplary polyketone film (0.5% CO content, average *M*_n_ = 2.2 × 10^4^ g × mol^−1^, 3 μm thickness) on a glass substrate in front of a striped background (**a**). Exemplary AFM image of a cut in a film (1.5 μm thickness) deposited from the same polyketone dispersion on a silicon substrate (**b**).

From the aqueous dispersions continuous films of the polyethylene with in-chain keto groups could be generated at room temperature (*cf*. Supplementary Table 6 and Supplementary Figs. 45, 46). Drop casting resulted in films with a thickness of 1–4 μm with arithmetic average roughness of 30–110 nm on typical AFM scan size (Fig. 7). In contrast, thinner films of 100–600 nm and roughness of 15–35 nm were accessible via vertical deposition. Polyketone films with low CO content are stiff and hard, whereas polyketones with higher CO content, which additionally have lower molecular weights, are softer or even sticky.

The decomposition of copolymers under UV-light (*λ* = 350–400 nm) was also studied on films of polyketones from solution copolymerization with ketone contents of 2–10 % (*cf*. Supplementary Figs. 47, 48). The average *M*_n_ decreases faster the higher the ketone content in agreement with chain cleavage being promoted by the ketone groups. Molecular weight distributions are broadened and show a small fraction with increased *M* after irradiation for CO contents ≤6%. This implies that, besides chain cleavage, crosslinking reactions might occur to minor extent which is reasonable due to the uncontrolled free-radical nature of Norrish reactions.

## Discussion

Polyethylene materials with in-chain keto groups are accessible at relatively moderate pressures of <350 atm by ethylene-carbon monoxide copolymerization in dimethyl carbonate solvent or aqueous conditions. The practicability of our method is underlined by the use of commercially available standard non-customized equipment exclusively in this study. Despite the different phase behavior, polymerizations in the heterophase system and in single-phase organic solutions yield similar ketone densities in the polymer chain, dependent on the CO partial pressure and the temperature. Desirable low CO contents could be achieved, sufficient for photodegradability but otherwise largely retaining characteristic PE properties. Notably, polyketones from dispersion polymerization differ from solution polymerized analogues by an up to fivefold increased branching density and thus lower crystallinity, and can contain a high-molecular weight fraction. Transfer reactions to the α-position of ketones and thus formation of mono-, di-, and trialkyl-branched ketones are rather prominent events, even more pronounced at the high local polymer concentration in the compartmented aqueous polymerization. Considerations on the relative reactivity of ethylene and CO with respect to the addition to the growing alkyl radical chain end reveal a ca. tenfold higher reactivity of CO (at 120 °C). Curiously, this coincides with reactivity in catalytic insertion copolymerization which, however, is characterized by an undesired propensity for formation of alternating motifs. This is not the case for the polymers studied here, which contain isolated in-chain keto groups randomly distributed along the chain and homogeneously distributed in a well-defined fashion also over all molecular weights. Notably, although carbon monoxide incorporations hamper chain growth as expected, the modified PEs obtained under our conditions possess desirable materials properties and can be processed by injection molding.

## Methods

### Materials

Materials and general considerations are available in the Supplementary Methods (Supplementary Information).

### Analytics

Methods for particle and polymer (microstructure) analytics, determination of materials properties, degradation and film forming behavior are described in the Supplementary Methods (Supplementary Information).

### Polymerization experiments

The general procedures for (co)polymerizations in organic solvents and water and detailed description of the high-pressure equipment used is available in the Supplementary Methods (Supplementary Information).

## Supplementary information

Supplementary Information

## Data Availability

The authors declare that all data supporting the findings of this study are available in the paper or its Supplementary Information file. Supplementary Information contains experimental and analytical procedures as well as setup details (Supplementary Figs. 1–2), data on polyketone and terpolymer microstructure analysis (Supplementary Figs. 3–13, 20–23, 32–39), analytics on materials properties (Supplementary Figs. 14–18, 24–27, 40–41), weathering study data (Supplementary Figs. 28–29), TEM images of polyketone particles (Supplementary Figs. 30–31), comparative graphs of products and reactions in different media (Supplementary Figs. 42–44), and information on film preparation and analysis (Supplementary Figs. 45–48). All data are available from the corresponding author upon reasonable request.
