# Supplementary material for: Photodegradable branched polyethylenes from carbon monoxide copolymerization under benign conditions
Source: Nat Commun. 2020 Jul 23;11:3693. doi: 10.1038/s41467-020-17542-5 (PMC7378081; doi:10.1038/s41467-020-17542-5)
Supplement: Supplementary file 1 — Supplementary Information [file 41467_2020_17542_MOESM1_ESM.pdf]

Supplementary Information

Photodegradable Branched Polyethylenes from Carbon Monoxide Copolymerization under Benign Conditions

Morgen et al.

Chair of Chemical Materials Science, Department of Chemistry, University of Konstanz, Universitätsstrasse 10, 78457 Konstanz, Germany

Contents

Supplementary Methods **4**

Materials.. 4

Analytical methods 4

General (co)polymerization procedure 7

Supplementary Figures **9**

NMR spectra of copolymers (solution polymerizations).. 9

ATR-IR spectra of copolymers (solution polymerizations)................................... 14

Analysis of microstructure by IR band deconvolution (solution polymerizations) 17

DSC traces of copolymers (solution polymerizations)... 20

GPC traces of copolymers (solution polymerizations)... 23

Determination of C_2H_4 solubility under polymerization conditions 25

CO contents in the C_2H_4-DMC-mixtures under polymerization conditions.......................... 27

Monomer reactivity ratio for free-radical CO-ethylene-copolymerization 28

Monomer reactivity ratio for catalytic CO-ethylene-copolymerization 29

Synthesis and characterization of terpolymers of E, CO and difunctional monomer 30

Specimen preparation and stress-strain tests... 35

Weathering study.. 38

TEM images of polyketone nanoparticles (aqueous polymerizations)................................. 40

NMR spectra of copolymers (aqueous polymerizations)...................................... 42

ATR-IR spectra of copolymers (aqueous polymerizations) 45

Analysis of microstructure by IR band deconvolution (aqueous polymerizations) 47

DSC traces of copolymers (aqueous polymerizations) 51

GPC traces of copolymers (aqueous polymerizations) 52

Effect of CO partial pressure and reaction temperature in different media 53

Film preparation and characterization 56

Film decomposition under UV-irradiation 58

Supplementary Tables 60

Monomer reactivity ratio for free-radical CO-ethylene-copolymerization 60

Monomer reactivity ratio for catalytic CO-ethylene-copolymerization 60

Synthesis and characterization of terpolymers of E, CO and difunctional monomer 61

Specimen preparation and stress-strain tests 62

Film preparation and characterization 63

Supplementary References 64

Supplementary Methods

Materials

Ethylene of 3.5 and carbon monoxide of 4.7 grade were supplied by Air Liquide. ^{13}CO with a purity of >99% was purchased from Euriso-Top. The water-soluble free-radical initiator potassium persulfate (KPS, >99%) was supplied by Sigma-Aldrich, and 2,2'-azobis[2-methyl-*N*-(2-hydroxyethyl)propionamide] (VA-086) by Wako Chemicals. Di-*tert*-butyl peroxide (95%, 5% *tert*-butyl hydroperoxide) was purchased from Fluka. Ethylene glycol dimethacrylate (EGDMA; >98%), Dimethyl carbonate (DMC; >99%), dichloromethane (DCM; >99%), tetrahydrofuran (THF; >99%), ethyl acetate (EE; >99%) and sodium dodecyl sulfate (SDS; >99%) were supplied by Sigma-Aldrich. Divinyl adipate (DVA; >99%) was purchased from TCI. Oxygen was removed from ethylene glycol dimethacrylate, dimethyl carbonate and ethyl acetate by freeze-pump-thaw degassing prior to use. THF and dichloromethane were degassed with a MB-SPS-800 solvent purification system by MBRAUN. Deionized water was purged with nitrogen with vigorous stirring for 2 h before the polymerization.

Analytical methods

For dynamic light scattering (DLS) a Malvern Nano-ZS ZEN 3600 particle sizer (173° backscattering) was used. Dilute samples were measured at 25°C. The autocorrelation function was analyzed using the Malvern dispersion technology software 7.12 algorithm which yields number-weighted particle size distributions and the polydispersity index (PDI) which is a measure for the width of the particle size distribution. Differential scanning calorimetry (DSC) was performed on a Netzsch DSC 204 F1 with a heating/cooling rate of 10 K min^{-1} on bulk polymer samples. Transmission electron microscopy (TEM) images were acquired on a Zeiss Libra 120 EF-TEM instrument (120 kV). The respective samples were diluted to a solids content of ca. 0.03 wt.-% and dialyzed in a Spectrum Laboratories Spectra/Por Dialysis Membrane 1, MWCO 6000-8000 with deionized water for at least two days. The resulting dispersions were dropped onto a TEM copper grid and dried for 2 h. Average particle sizes from TEM images were determined by measuring the average diameter of 100 particles. Optical microscopy images were acquired in reflected light mode on a Leica DM4000 M equipped with an LMscope C-Mount connected to a Canon EOS 800D camera. GPC (Gel Permeation Chromatography) was performed on a PolymerChar GPC-IR instrument equipped with PSS Polefin Linear XL columns (3 × 30 cm, additional guard column) at 160 °C in 1,2-dichlorobenzene or 1,2,4-trichlorobenzene and 1.0 mL×min^{-1} flow rate. The

polymer was detected by an integrated four-capillary viscometer and an IR4 dual wavelength infrared detector (selective for methylene, methyl and/or carbonyl bands). Calibration was done versus narrow polystyrene standards using universal calibration method. The raw data was evaluated with PSS WinGPC UniChrom software. NMR spectra were recorded on a Bruker Avance III 600 spectrometer. The chemical shifts were referenced to the respective solvent signals. The high temperature measurements of polymers were carried out in 1,1,2,2-tetrachlorethane-d_2 at 100°C (^{1}H NMR: 6.00 ppm, ^{13}C NMR: 73.78 ppm). ATR-IR spectra of polymers were measured on a Spectrum 100 by Perkin Elmer. For calculation of the comonomer incorporation, the ratio of the intensity of the C=O signal (1700-1720 cm^{-1}) to the intensity of the C-H signal of the PE at ~2915 cm^{-1} was calculated and referenced with polyketone samples with known C=O content synthesized via ADMET copolymerization and hydrogenation (Supplementary Figure 1).[1] Films of polyketones were deposited from dialyzed aqueous copolymer dispersions at room temperature by drop casting or vertical deposition on cleaned glass or silicon substrates. The decomposition of polyketone films on glass substrates was investigated by exposing the samples to UV light under a Proma UV exposure unit (350-400 nm, 4 × 8W tubes) for 6 h. AFM measurements were carried out in intermittent contact mode on a NanoWizard device by JPK Instruments equipped with a Tap300 cantilever from BudgetSensors (300 kHz resonance frequency, 40 N×m^{-1} force constant). Images were recorded with a line scanning rate of 0.1 or 0.2 Hz. Tensile testing experiments were carried out with dogbone specimens (75 × 12.5 × 2 mm^3, 5 mm min^{-1} crosshead speed, 50 mm specimen holder distance, ISO 527-2, type 5A) which were prepared by injection molding using a HAAKE Minijet II (Thermo Scientific). Depending on the amount of difunctional comonomer in the processed polymer, cylinder temperatures of 150-190°C, mold temperatures of 60-70°C and injection pressures of 500-600 bar were applied. Tensile tests were performed on a Zwick 1446 RetroLine testControl II instrument, controlled and evaluated with the Zwick test Xpert software. The weathering of specimens was performed in an Atlas SUNTEST CPS+ connected to a SunFlood flooding device. The UV-light of the lamp was filtered to simulate natural sunlight on sea level. The samples were permanently flushed with distilled water of 35°C and irradiated with 30 W m^{-2}.

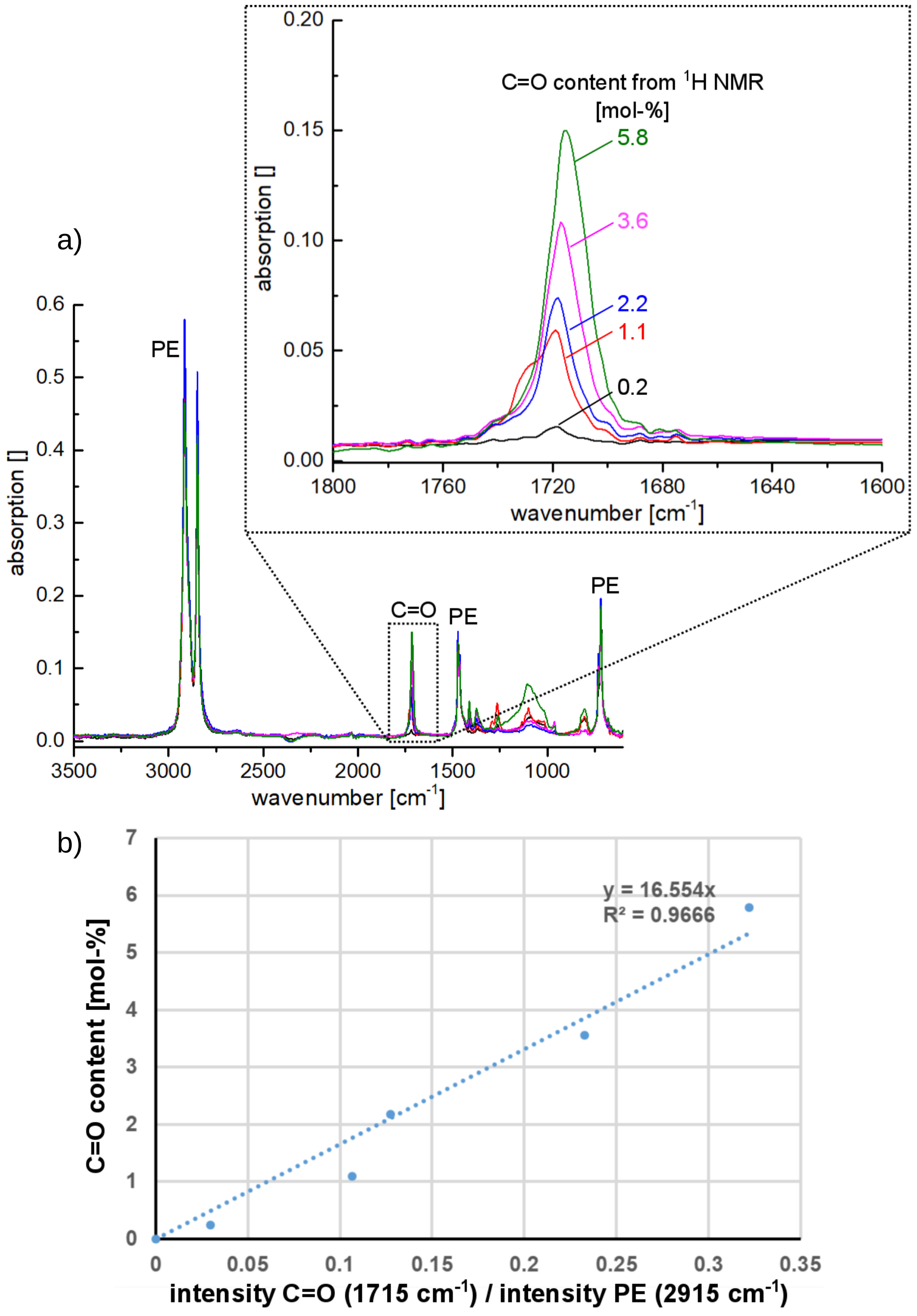

Supplementary Figure 1. Referencing of IR spectra. Polyketones with known carbonyl content (from ^{1}H NMR) were analyzed by ATR-IR spectroscopy (a). The samples were synthesized via ADMET copolymerization of different ratios of docosa-1,21-dien-11-one and undeca-1,10-diene followed by hydrogenation.[1] The intensity ratios of the carbonyl band at 1715 cm^{-1} and the band of the C-H vibration at 2915 cm^{-1} is proportional to the concentration of C=O groups in the polymer (b).

General (co)polymerization procedure

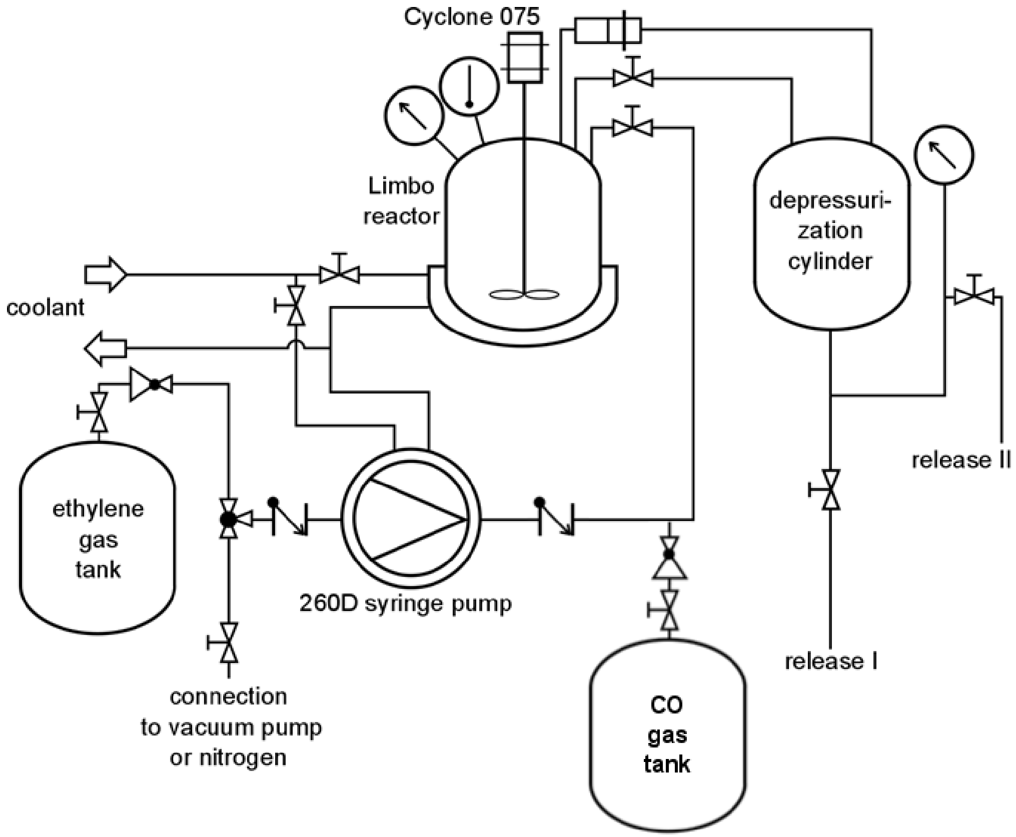

Supplementary Figure 2. Flow diagram of the setup used. It includes a 260D syringe pump, a depressurization cylinder and a Limbo reactor equipped with a Cyclone 075 stirrer. Reactor and pump can be cooled with water. CAUTION: The design and operation of any apparatus for working with high pressure ethylene requires an individual safety analysis and trained personnel.

For polymerization in organic solvents di-*tert*-butyl peroxide (44 mg, 0.3 mmol) was dissolved in 75 mL of the respective solvent under a nitrogen atmosphere. For reactions in aqueous media KPS (81 mg, 0.3 mmol) or VA-086 (87 mg, 0.3 mmol) was added to 150 mL of oxygen-free deionized water. Polymerizations initiated with VA-086 were carried out in the presence of 0.5 wt.-% SDS (0.75 g, 2.6 mmol) as a stabilizer. All polymerization experiments were performed in a 285 mL Limbo reactor by Büchi, Uster constructed from Hastelloy equipped with a Cyclone 075 magnetically coupled mechanical pitched blade stirrer, in- and outlet valve, a temperature sensor and a digital pressure sensor, and an electric heating mantle (Supplementary Figure 2). The reactor was evacuated and purged with nitrogen three times before the respective reaction mixture was transferred into the reactor via cannula, and the reactor was closed. The stirring rate was adjusted

to 1000 rpm and the temperature was set to 20°C. 0.5 to 30 bar of carbon monoxide were filled into the reactor and the system was equilibrated for 20 min. The reactor was then pressurized with ethylene to a total pressure of 110 bar for reactions with water or to 60 bar for reactions in organic solvents, respectively. Compression of the supercritical ethylene was carried out with a Teledyne Isco 260D Syringe Pump. After equilibrating temperature and pressure for 30 min, the reactor was closed to the ethylene supply and the internal temperature was raised to the desired polymerization temperature within 30 min. After the desired reaction time, the reactions were stopped by venting followed by cooling to room temperature. For determination of the polymer yield and for analysis of the polymer properties the respective organic solvent was removed under reduced pressure and the residual polymer was freed from solvent at 60°C under vacuum for 24 h. The solids content of aqueous polyketone dispersions was determined by evaporating 100 mL of the mixture in a rotary evaporator under reduced pressure. The residue was washed excessively with water, dried under vacuum at 60°C and weighed.

Supplementary Figures

NMR spectra of copolymers (solution polymerizations)

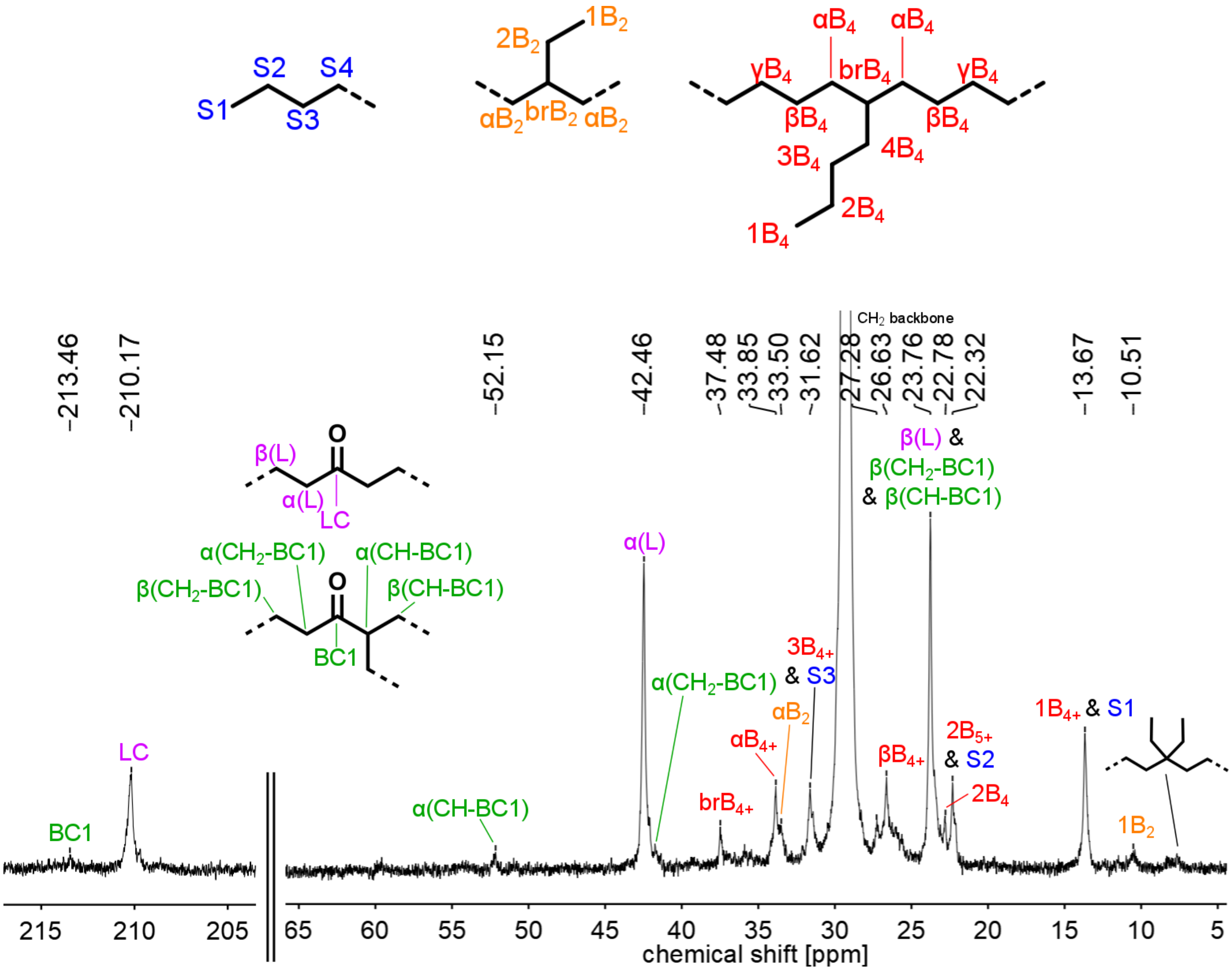

Supplementary Figure 3: ^{13}C NMR spectrum of a polyketone. Inverse-gated spectrum at 100°C in 1,1,2,2-tetrachloroethane-d_2 of a polyketone synthesized in dimethyl carbonate with CO content of 5.0 mol-%. Signals of branches or backbone methylene groups in close proximity to branches were assigned according to [2]. LC: linear carbonyl. BC: α-branched carbonyl.

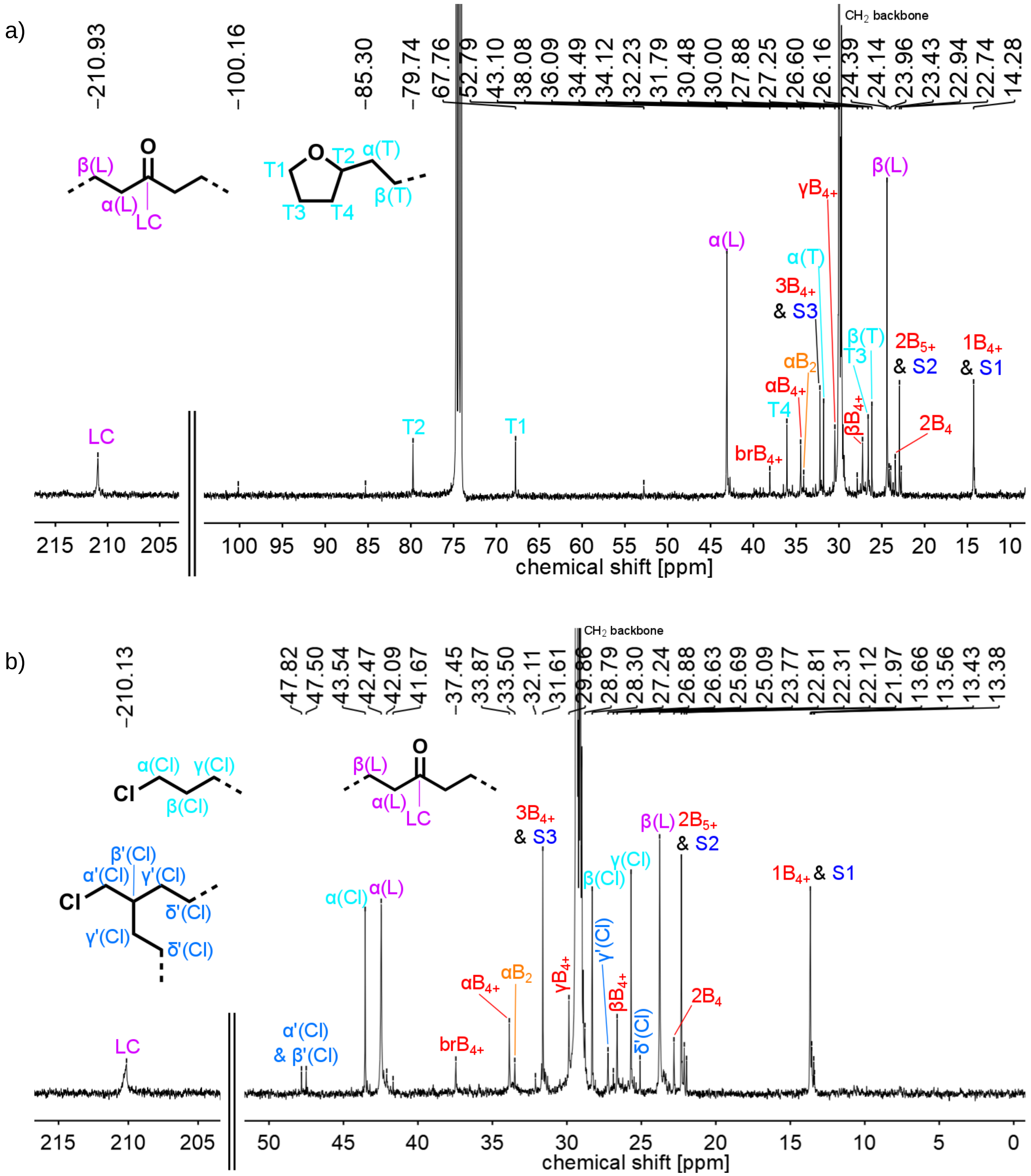

Supplementary Figure 4: ^{13}C NMR spectra of polyketones. Inverse-gated spectra at 100°C in 1,1,2,2-tetrachloroethane-d_2 of polyketones synthesized in tetrahydrofuran (a) or in dichloromethane (b) with CO contents of 4.1 or 3.8 mol-%, respectively. Signals were assigned according to [2], [3] and [4]. LC: linear carbonyl.

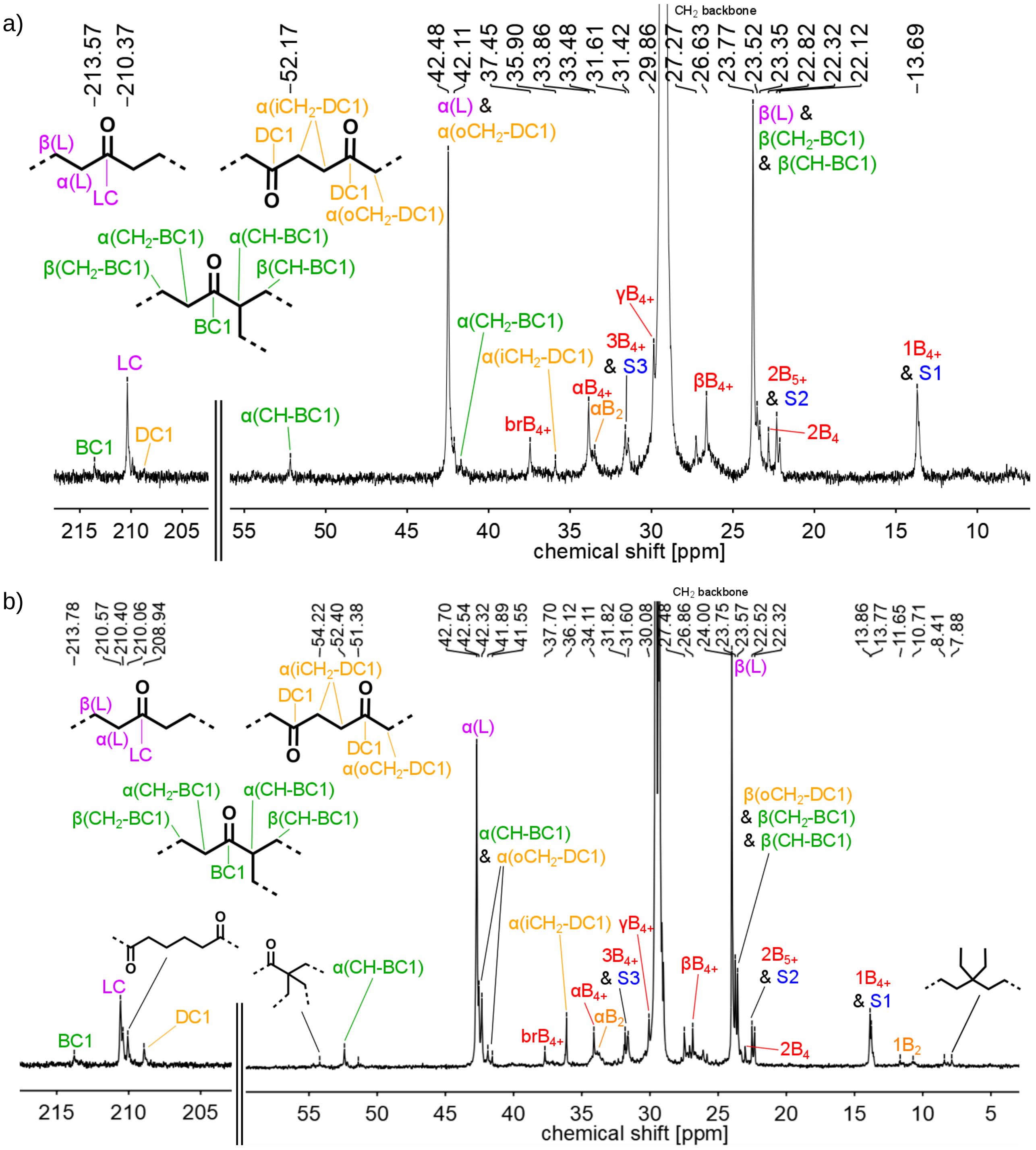

Supplementary Figure 5: ^{13}C NMR spectra of polyketones. Inverse-gated spectra at 100°C in 1,1,2,2-tetrachloroethane-d_2 of polyketones synthesized in ethyl acetate (a) and dimethyl carbonate (b) with CO contents of 4.8 mol-% and 13.2 mol-%, respectively. Signals of branches or backbone methylene groups in close proximity to branches were assigned according to [2]. LC: linear carbonyl. BC: α-branched carbonyl. DC: double carbonyls.

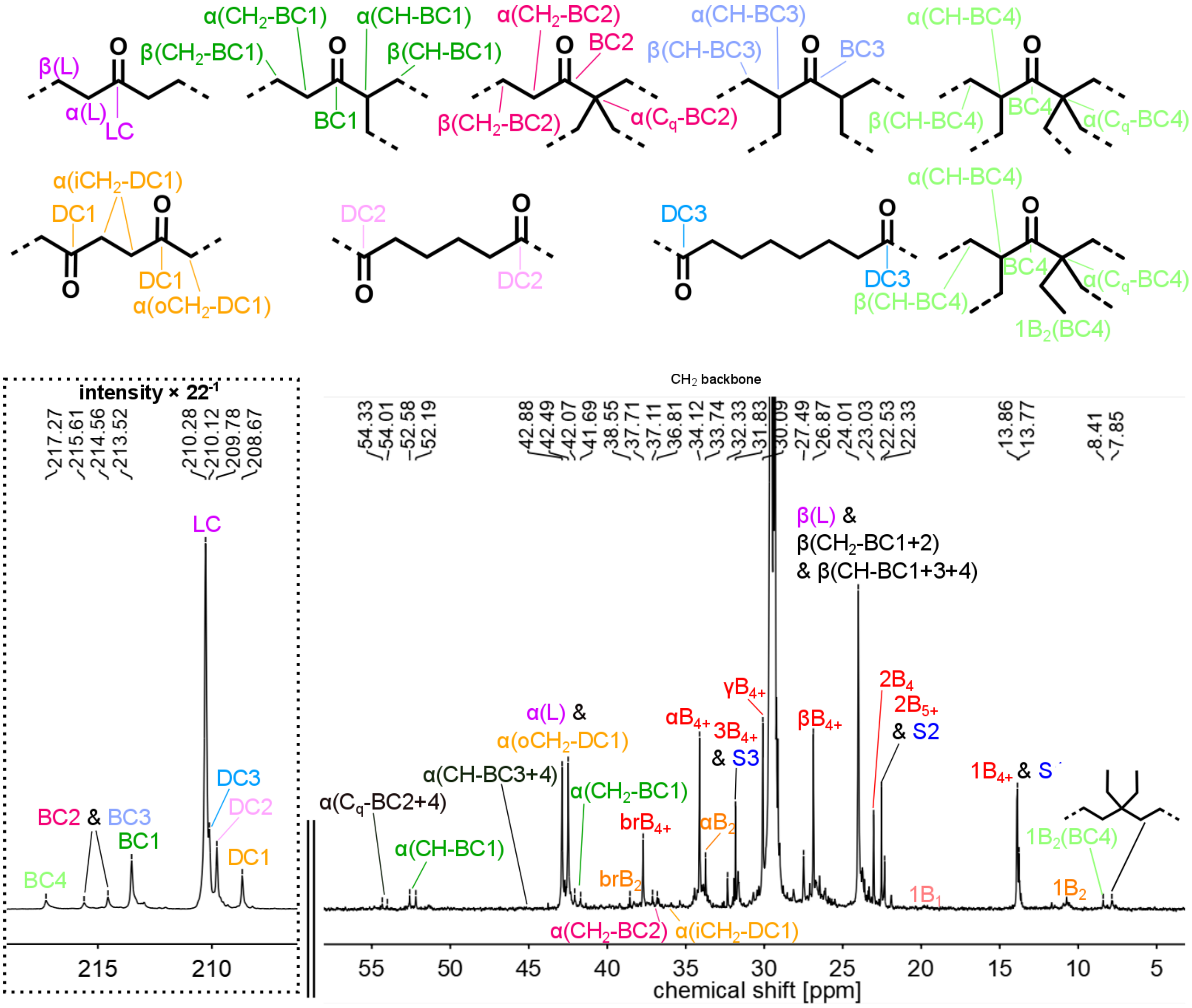

Supplementary Figure 6: ^{13}C NMR spectrum of a ^{13}C-labeled polyketone. Inverse-gated spectrum at 100°C in 1,1,2,2-tetrachloroethane-d_2 of a polyketone synthesized in dimethyl carbonate with ^{13}CO content of 5.6 mol-%. Peaks of the different ketone groups, α- and β-carbons were assigned in accordance to HMBC and HSQC (Supplementary Figures 7, 34), spectra of reference compounds and [5]. Signals of branches or backbone methylene groups in close proximity to branches were assigned according to [2]. LC: linear carbonyl. BC: α-branched carbonyl. DC: double carbonyls.

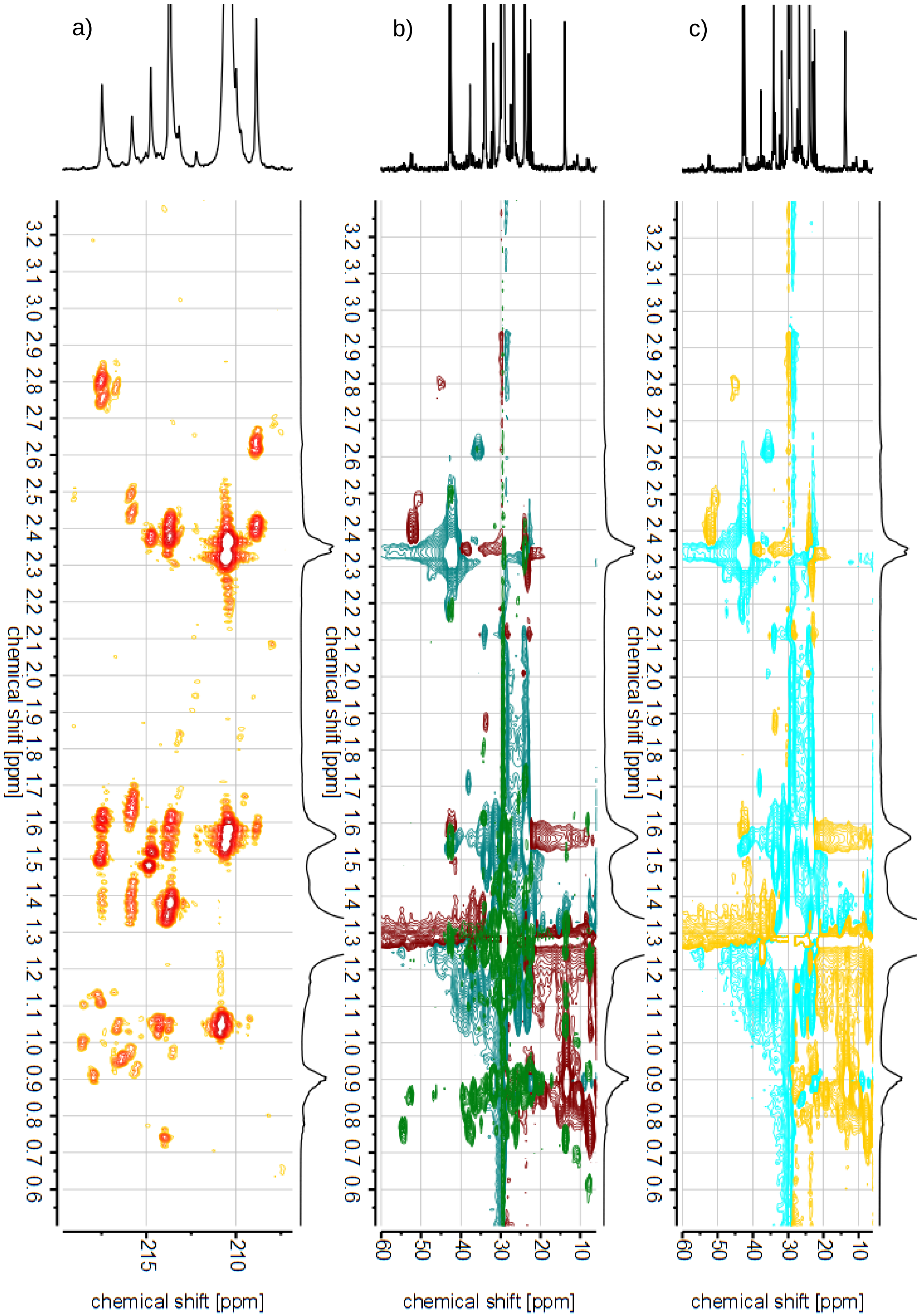

Supplementary Figure 7: 2D NMR spectra of a ^{13}C-labeled polyketone. ^{1}H-^{13}C HMBC of carbonyl carbons (207-220 ppm; a), overlap of ^{1}H-^{13}C HMBC and HSQC (5-60 ppm; b) and ^{1}H-^{13}C HSQC (5-60 ppm; c).

ATR-IR spectra of copolymers (solution polymerizations)

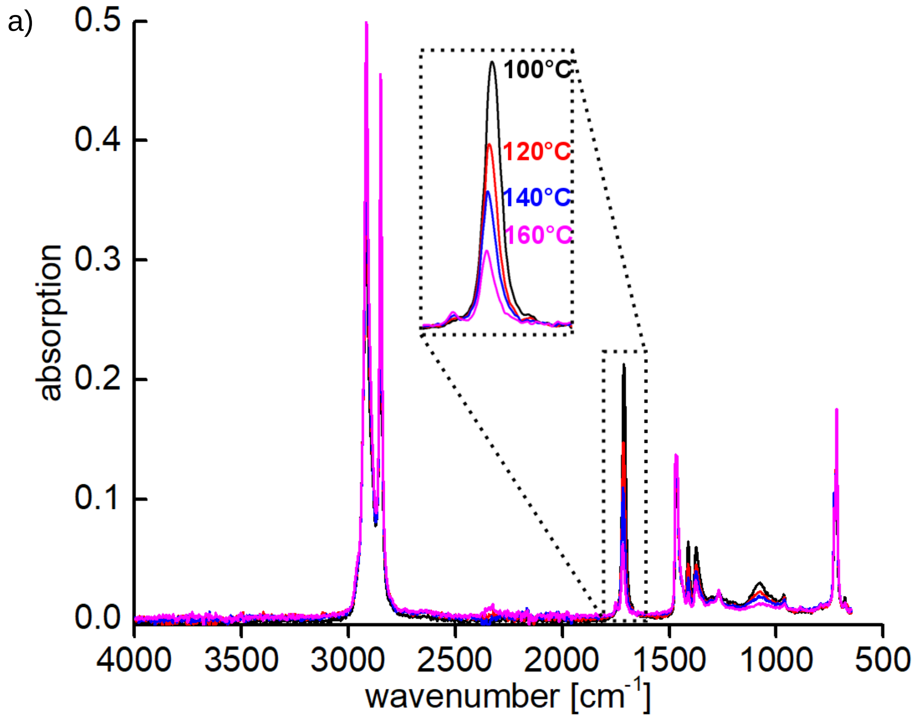

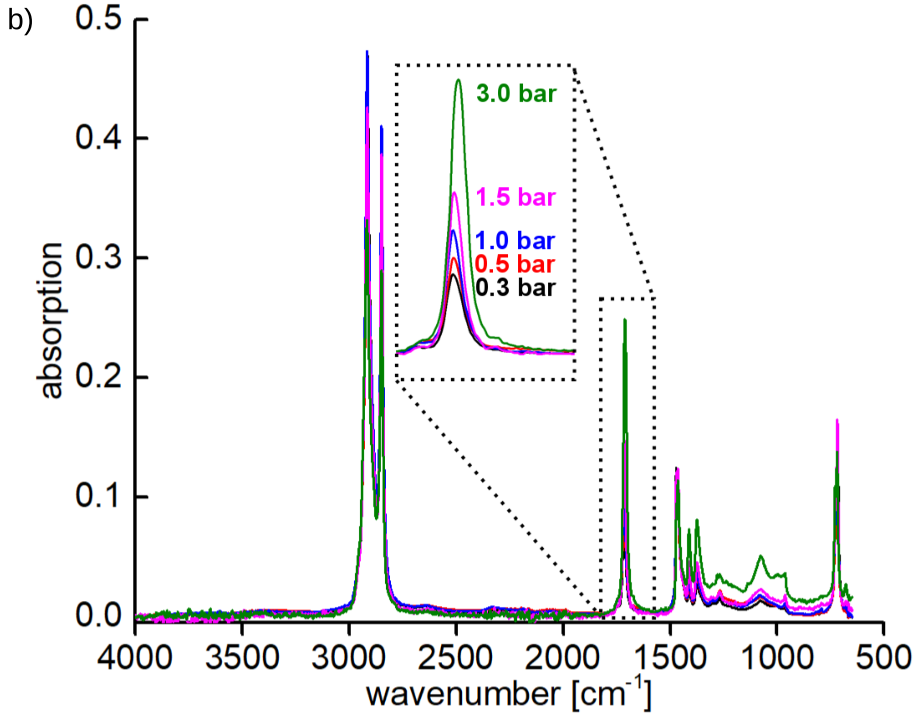

Supplementary Figure 8: ATR-IR spectra of polyketones. Synthesis in dimethyl carbonate at different polymerization temperatures (a) and initial carbon monoxide pressures (b).

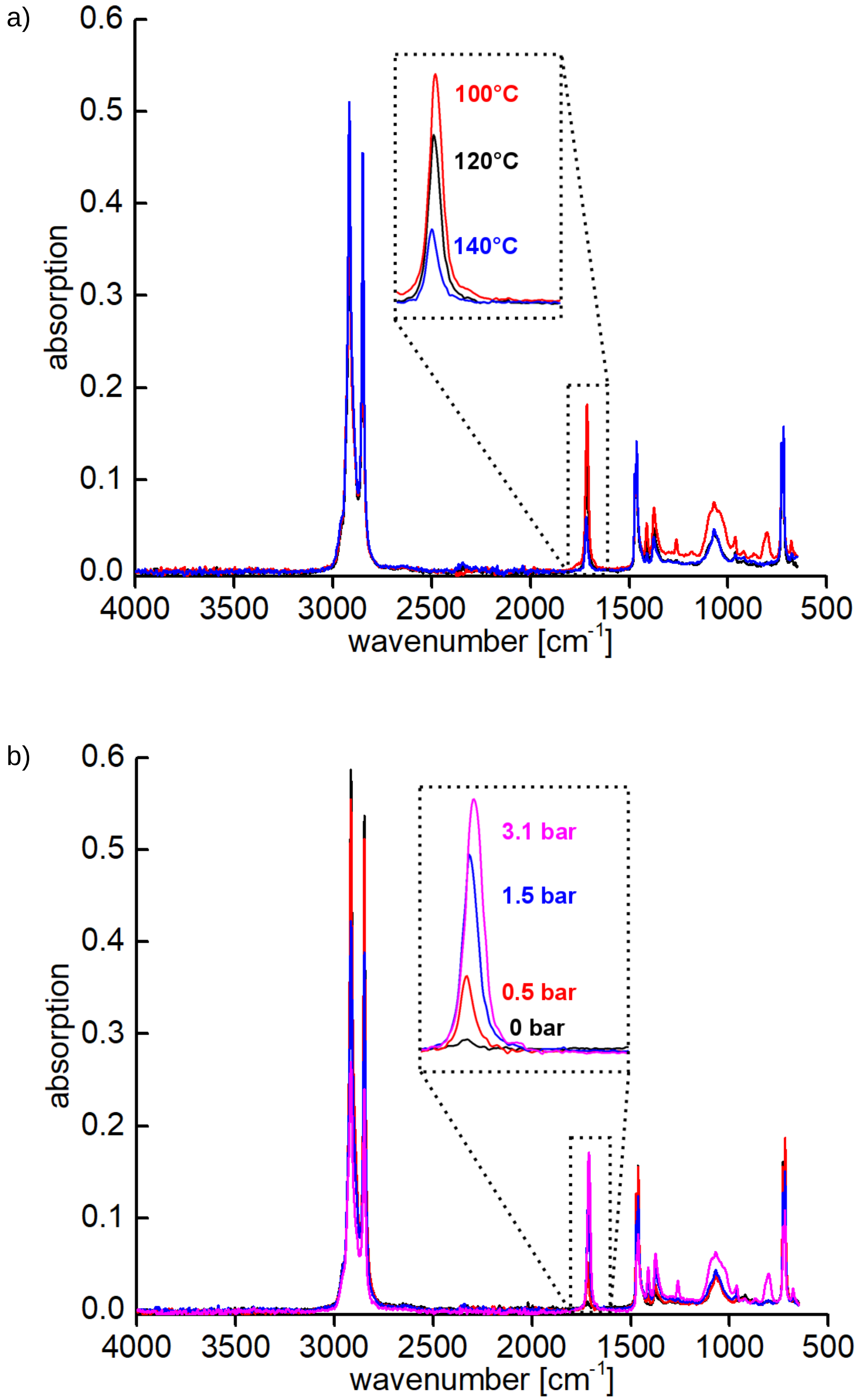

Supplementary Figure 9: ATR-IR spectra of polyketones. Synthesis in tetrahydrofuran at different polymerization temperatures (a) and initial carbon monoxide pressures (b).

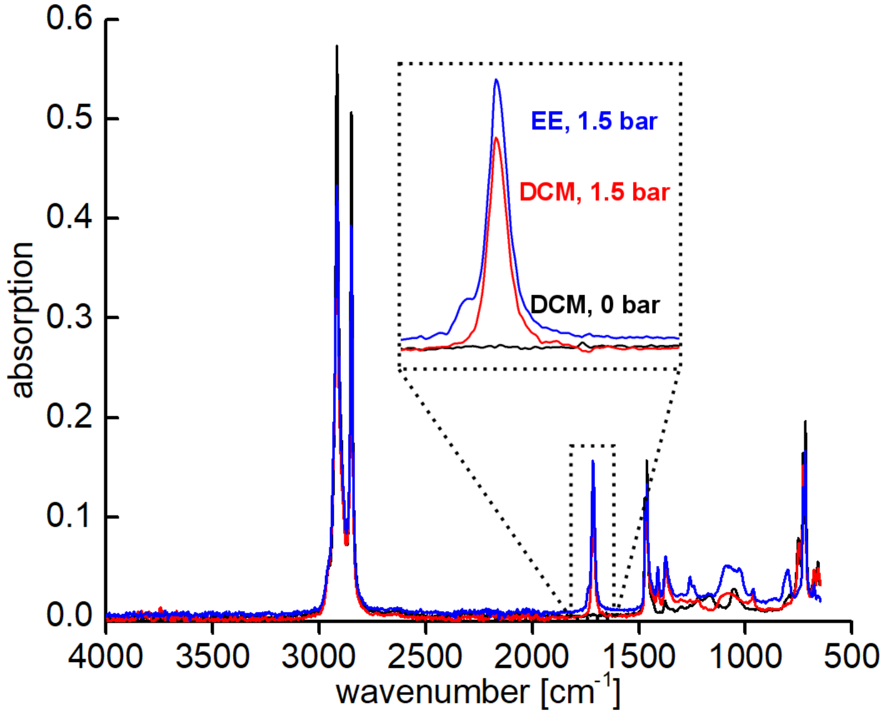

Supplementary Figure 10: ATR-IR spectra of polyketones. Synthesis in dichloromethane or ethyl acetate at different initial carbon monoxide pressures.

Analysis of microstructure by IR band deconvolution (solution polymerizations)

The IR absorption band of carbonyls in polyketones from Pd-catalyzed copolymerization was found to be indicative for the copolymers' microstructure in terms of spatial proximity of ketone groups to each other.[6] An increasing concentration of ketone groups in the surrounding PE matrix shifts the IR absorption maximum of a given C=O group gradually from 1718 cm^{-1} to 1690 cm^{-1}. Empirically, one can determine the amount of isolated ketones in a PE matrix (α-case), ketones in closer proximity to each other (β-case) and strictly alternating polyketones (γ-case) by deconvolution of the IR band with Lorentzian functions at three different spectral positions (Supplementary Figure 11). Polyketones from free-radical, especially dispersion, copolymerization additionally contain highly branched ketones which call for a fourth case (α')(Supplementary Figures 33 and 34). In contrast, the portion of highly branched ketones in polyketones from free-radical solution copolymerization is negligibly small (compare Supplementary Figures 6 and 33).

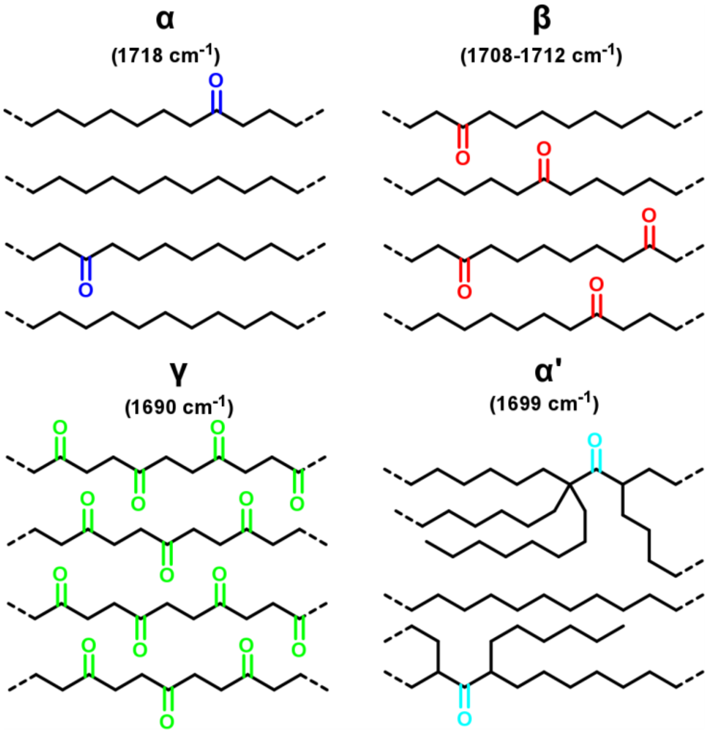

Supplementary Figure 11: Schematic representation of different ketones in polyketones. Spectral positions used in IR band deconvolution are given for: isolated ketones (α), ketone groups closer to each other (β), strictly alternating polyketone (γ) and highly branched, isolated ketone groups (α').

As a reference for isolated ketone groups in a PE matrix, we used hydrogenated, linear long-spaced polyketones from ADMET copolymerization of docosa-1,21-dien-11-one and undeca-1,10-diene with low ketone concentration which show a C=O band at 1718 cm^{-1}.[1] However, the ketones in these samples have long distances to the neighboring C=O's of the same chain, the IR absorption band starts to shift towards lower wavenumbers for ketone contents >3 mol-% (Supplementary Figure 12). Presumably, this arises from nearby C=O's of neighboring polymer chains in the solid. The maximum of the Lorentzian for ketones of the β-case was fitted in between 1708-1712 cm^{-1} since its spectral position is expected to be gradually decreasing the higher the local ketone concentration. The value of 1690 cm^{-1} for the γ-case was taken from literature.[6] 1699 cm^{-1} for the α'-case was determined by fit optimization on several IR spectra of polyketones containing highly branched C=O groups.

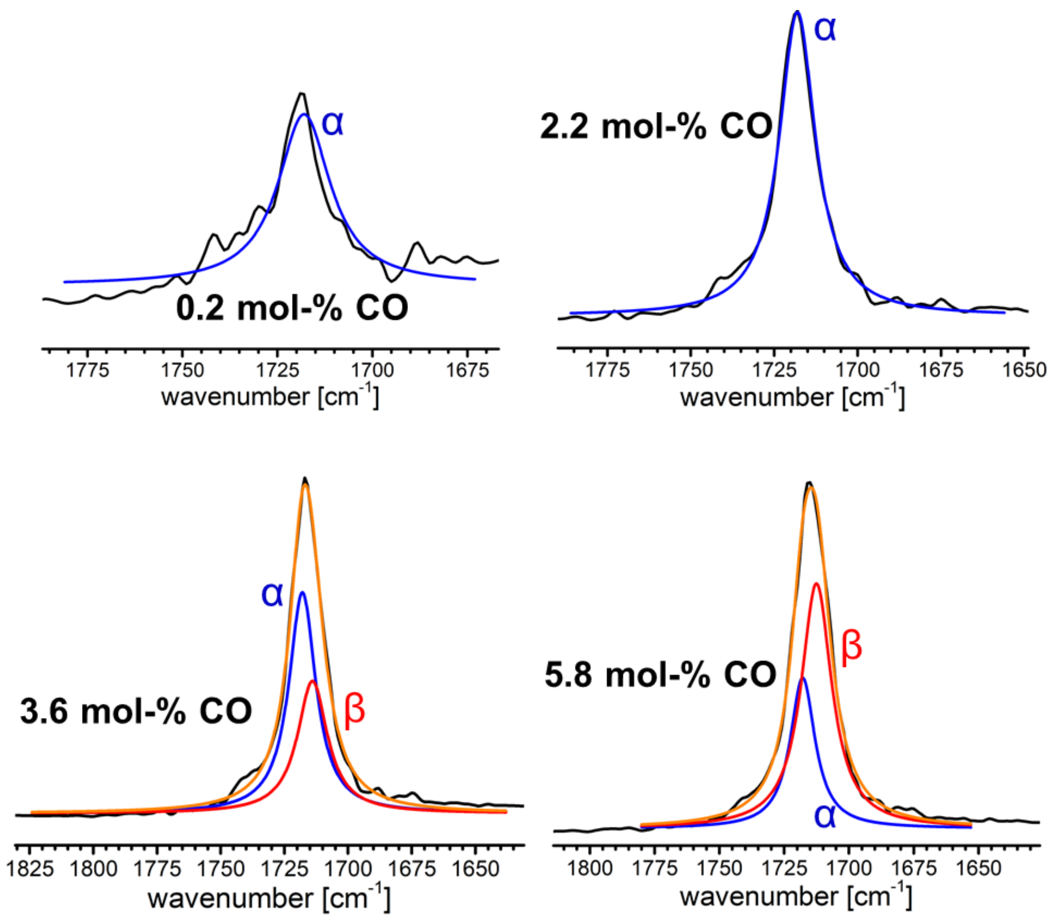

Supplementary Figure 12: ATR-IR band deconvolution. Samples: hydrogenated, long-spaced polyketones from ADMET copolymerization of docosa-1,21-dien-11-one and undeca-1,10-diene with different amounts of ketone groups.

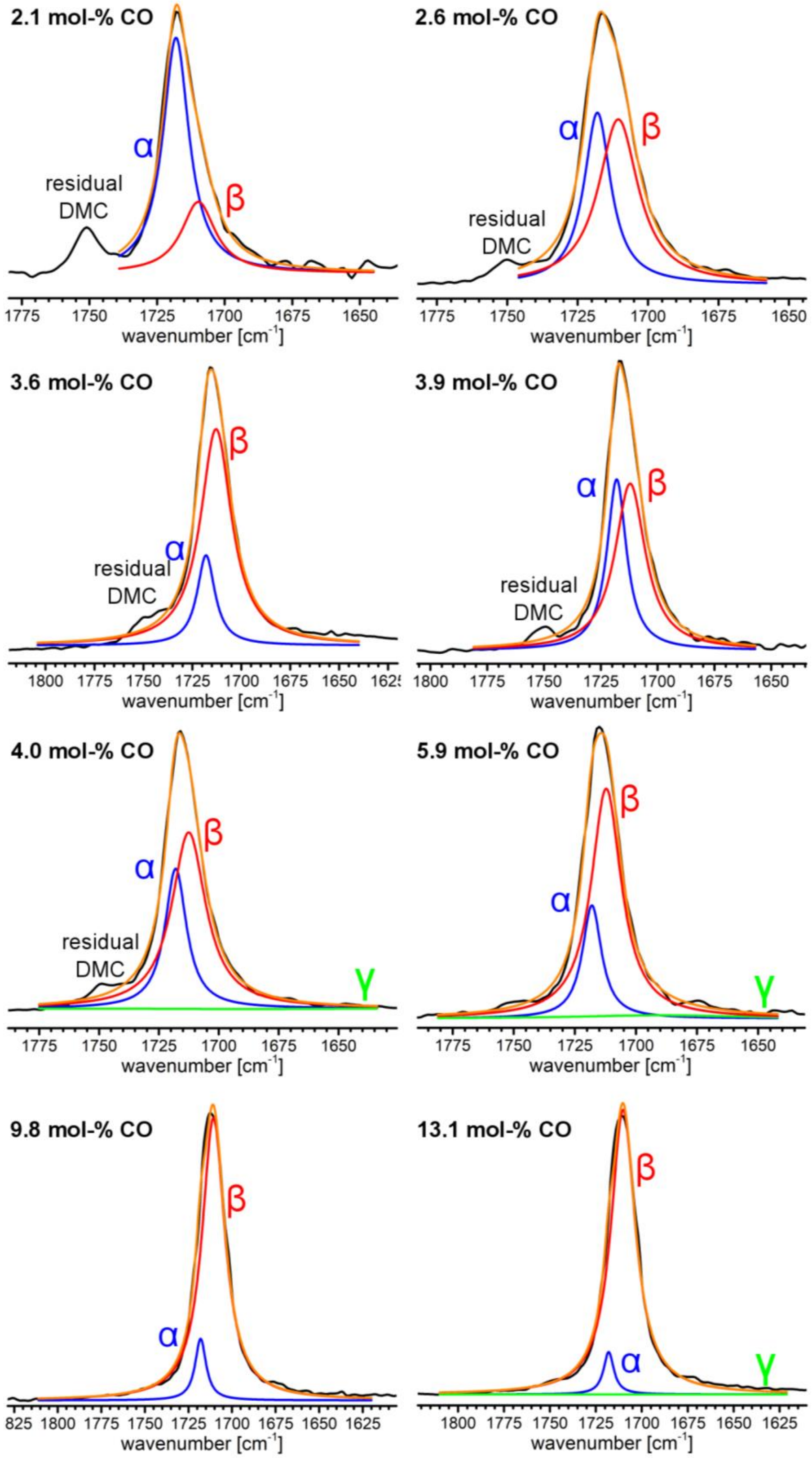

Supplementary Figure 13: ATR-IR band deconvolution. Samples: polyketones from free-radical solution copolymerization with different carbonyl contents.

DSC traces of copolymers (solution polymerizations)

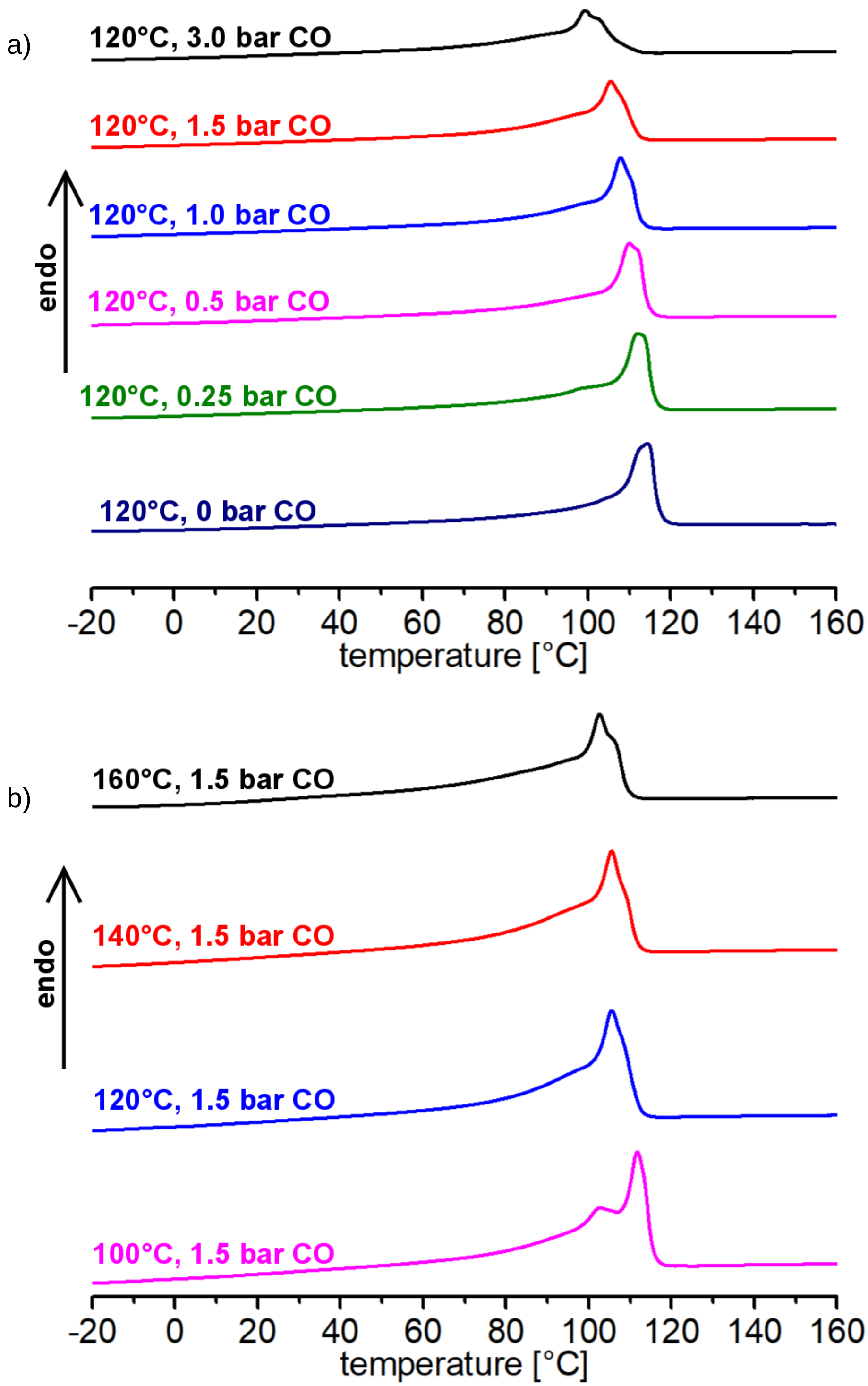

Supplementary Figure 14: DSC data of different polymers. Second DSC heating curves of bulk PE and polyketones synthesized in dimethyl carbonate at different initial carbon monoxide pressures (a) and polymerization temperatures (b).

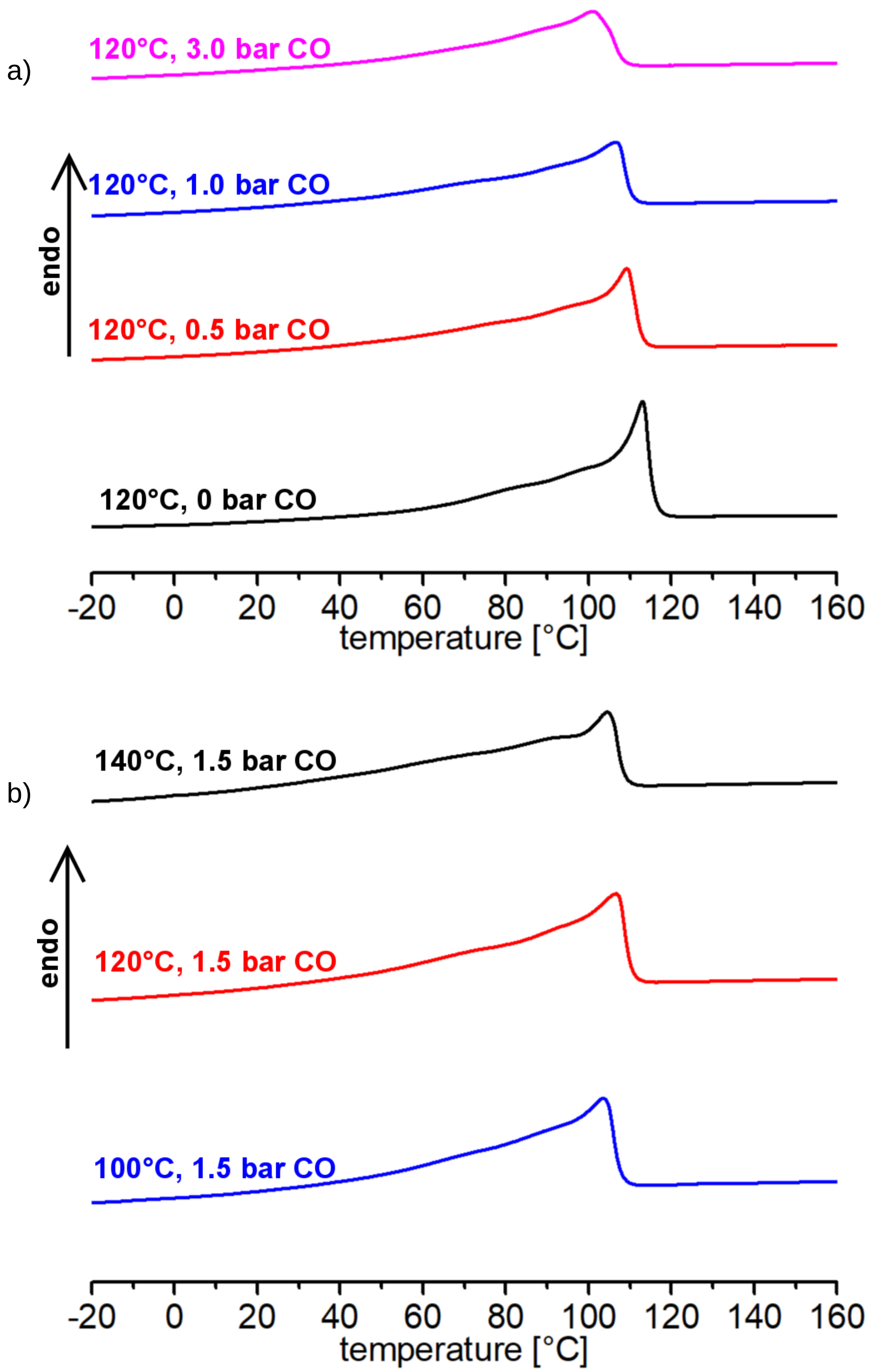

Supplementary Figure 15: DSC data of different polymers. Second DSC heating curves of bulk PE and polyketones synthesized in tetrahydrofuran at different initial carbon monoxide pressures (a) and polymerization temperatures (b).

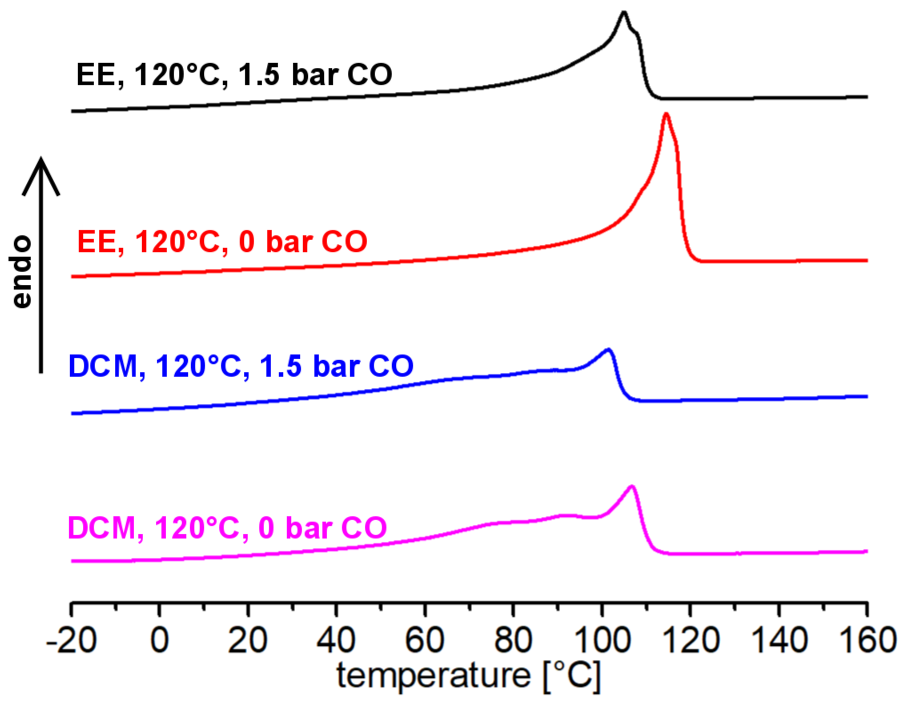

Supplementary Figure 16: DSC data of different polymers. Second DSC heating curves of bulk PE and polyketones synthesized in dichloromethane or ethyl acetate at different initial carbon monoxide pressures.

GPC traces of copolymers (solution polymerizations)

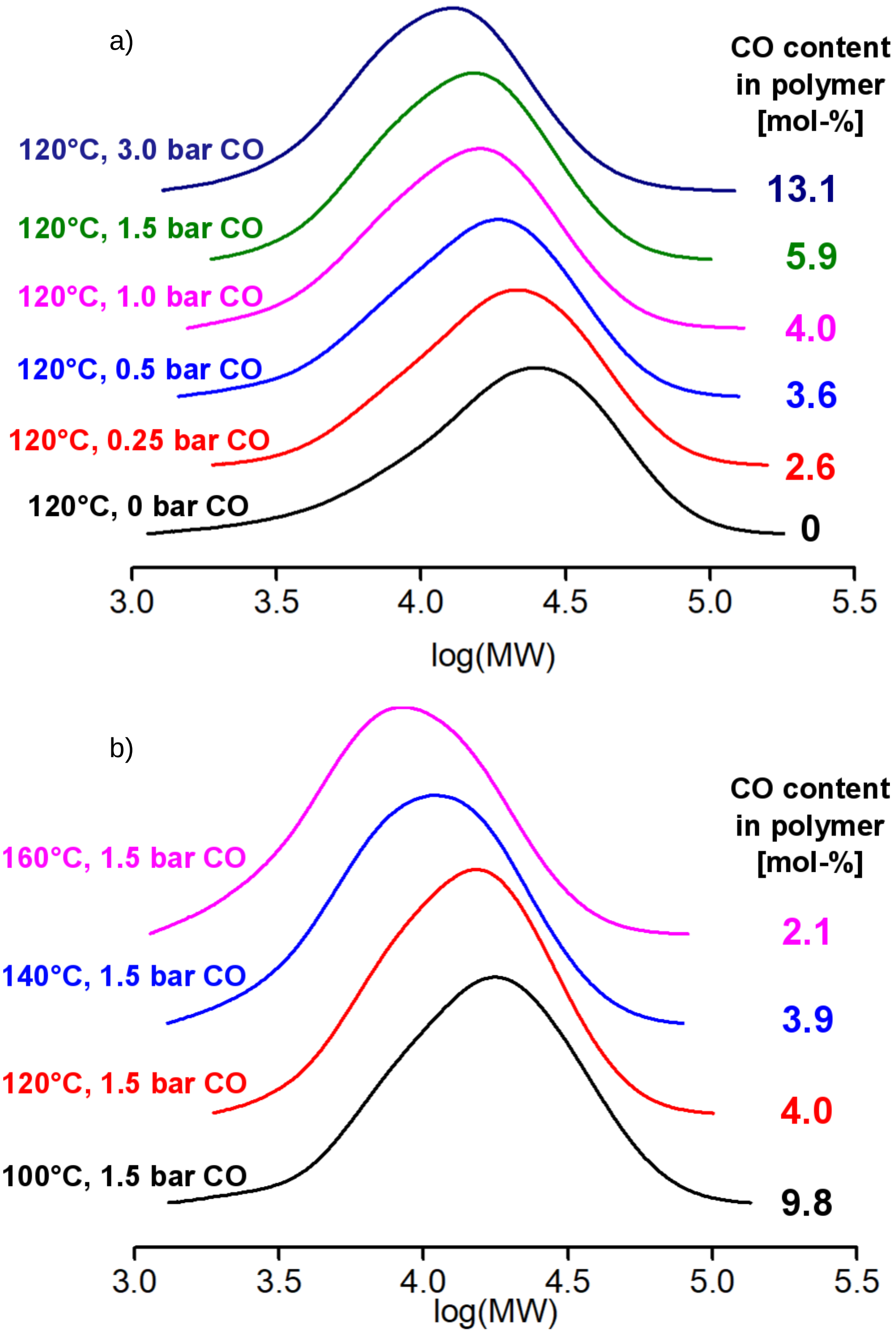

Supplementary Figure 17: GPC data of polymers. Molecular weight distributions of PE and polyketones synthesized in dimethyl carbonate with different initial carbon monoxide pressures (a) and polymerization temperatures (b).

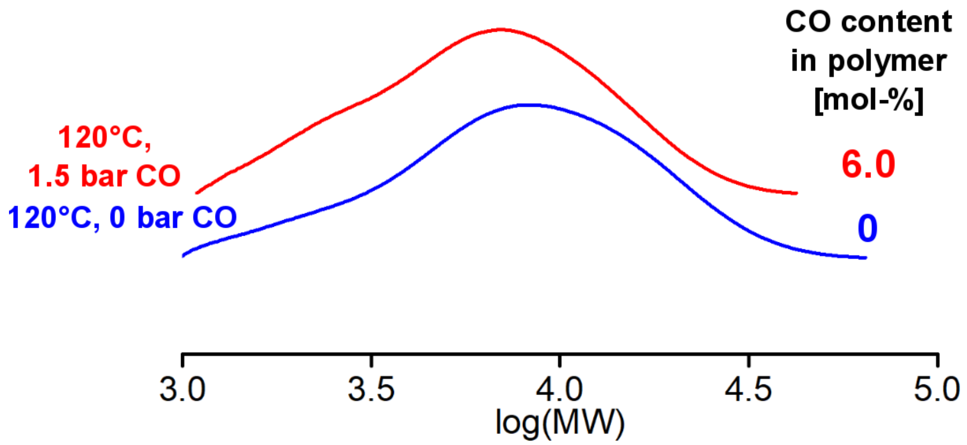

Supplementary Figure 18: GPC data of polymers. Molecular weight distributions of PE and polyketone synthesized in ethyl acetate with different initial carbon monoxide pressures.

Determination of C_2H_4 solubility under polymerization conditions

The reactor under 1 atm of nitrogen was filled with 75 mL (80 g, 0.89 mol) of dimethyl carbonate which was stirred with 200 rpm and then heated to the respective temperatures (120 or 140°C). The known volume of the syringe pump and the tubing between pump and reactor was filled with ethylene at a defined temperature and pressure which allows the calculation of the amount of ethylene by its equation of state. We used an equation whose correction terms were determined from experimental data.[7]

Ethylene amount in pump before pressurization of the reactor:

T = 19°C, p = 81.5 bar, V = 266.4 mL (syringe pump) + 7 mL (tubing)

→ Calculated with equation of state: n(ethylene) = 3.2 mol (89 g)

Ethylene amount in pump after pressurization of the heated reactor (120°C, 300 bar):

T = 19°C, p = 301.0 bar, V = 35.8 mL (syringe pump) + 7 mL (tubing)

→ Calculated with equation of state: n(ethylene) = 0.67 mol (19 g)

Amount of ethylene in the reactor: 2.5 mol (70 g)

Ethylene amount in pump after pressurization of the heated reactor (140°C, 300 bar):

T = 19°C, p = 300.0 bar, V = 46.7 mL (syringe pump) + 7 mL (tubing)

→ Calculated with equation of state: n(ethylene) = 0.83 mol (23 g)

Amount of ethylene in the reactor: 2.3 mol (65 g)

The volume of dimethyl carbonate increases upon dissolving ethylene. To determine the amount of dissolved ethylene in the organic solvent compared to supercritical ethylene above the reaction mixture, the volume of the first phase has to be measured under polymerization conditions. The total reactor volume is 269 mL.

In order to observe the expansion of the solvent, the reactor was filled with a dispersion of 75 mL of dimethyl carbonate and 1 g of carbon black. While slowly stirring (100 rpm) the reactor was heated to 120°C followed by careful pressurization with ethylene up to 300 bar. The mixture was equilibrated for 1 h followed by cooling and slow depressurization. The carbon black deposited on the complete reactor wall, the reactor lid and even in the fittings connected to the latter. This

observation suggests that the reactor contains a monophasic mixture of ethylene and dimethyl carbonate under polymerization conditions and that there is no separate supercritical ethylene phase.[8] This is also in accordance with the fact that formed polyketones deposited on both the reactor walls and the lid in free-radical solution copolymerizations of CO and ethylene. Note that the deposition of carbon black in a control experiment with only 30 bar of ethylene indicated two phases and ~30% volume expansion of DMC.

With the determined amount of ethylene, the known amount of dimethyl carbonate (80 g, 0.89 mol) and the total reactor volume of V = 269 mL, the concentrations of ethylene under the respective conditions can be calculated:

Concentration of ethylene in dimethyl carbonate at 120°C and 300 bar:

c = 9.3 mol×L^{-1}, mole fraction of ethylene x = 0.74, 47 wt.-%, mixture density ρ = 0.56 g×mL^{-1}

Concentration of ethylene in dimethyl carbonate at 140°C and 300 bar:

c = 8.7 mol×L^{-1}, mole fraction of ethylene x = 0.72, 45 wt.-%, mixture density ρ = 0.54 g×mL^{-1}

Comparison with literature values of mole fractions of ethylene in other solvents under similar conditions:[9]

x = 0.775 (in hexane, 101.3 bar, 100°C)
x = 0.670 (in hexane, 101.3 bar, 150°C)
x = 0.458 (in ethanol, 121.3 bar, 75°C)
x = 0.224 (in acetone, 70.9 bar, 100°C)
x = 0.457 (in ethyl acetate, 70.9 bar, 100°C)
x = 0.411 (in ethyl acetate, 70.9 bar, 125°C)
x = 0.359 (in ethyl acetate, 70.9 bar, 150°C)

CO contents in the C_2H_4-DMC-mixtures under polymerization conditions

Since the reaction mixtures contain ~45 wt.-% (73 mol-%) ethylene, we assume a complete solubility of the initial small CO amounts in this medium. Carbon monoxide was filled into the reactor containing 75 mL of dimethyl carbonate at pressures of 0.5 to 3 bar at a temperature of 20°C prior to pressurization with ethylene. Solvent expansion can be neglected under these conditions, so that the total amount of CO in the reactor n_{tot} can be calculated as the sum of the CO in the gas phase n_{gas} (with a volume of V_{gas} = 194 mL) and the CO dissolved in the dimethyl carbonate n_{sol} (V_{sol} = 75 mL). n_{gas} is approximated with the ideal gas law.

$$n_{\text{tot}} = n_{\text{sol}} + n_{\text{gas}} = c \cdot V_{\text{sol}} + \frac{pV_{\text{gas}}}{RT} \qquad \text{(Supplementary Equation 1)}$$

The solubility of carbon monoxide in dimethyl carbonate at 20°C and 1 bar was estimated from solubility data for other solvents:[10]

$x = 1.73\times10^{-3}$ (in heptane, 1 bar, 25°C)
$x = 9.92\times10^{-4}$ (in cyclohexane, 1 bar, 24°C)
$x = 3.74\times10^{-4}$ (in methanol, 1 bar, 20°C)
$x = 4.88\times10^{-4}$ (in ethanol, 1 bar, 20°C)
$x = 5.495\times10^{-4}$ (in 1-propanol, 1 bar, 20°C)
$x = 6.49\times10^{-4}$ (in acetone, 1 bar, 20°C)
$x = 8.397\times10^{-4}$ (in methyl acetate, 1 bar, 20°C)
$x = 9.83\times10^{-4}$ (in ethyl acetate, 1 bar, 20°C)
$x = 1.17\times10^{-3}$ (in propyl acetate, 1 bar , 25°C)
$x = 1.229\times10^{-3}$ (in diethyl ether, 1 bar, 20°C)

A possible parameter for quantification of solvents' properties are their dielectric constants ε and the absolute value of their dipole moments d. We indeed find an empirical correlation of x and respective ε-values of the solvents in terms of higher CO solubility in solvents with a lower ε. For dimethyl carbonate $\varepsilon = 3.2$ and $d = 0.91$ D have been reported.[11,12] This is close to the values of diethyl ether ($\varepsilon = 4.33$, $d = 1.1$ D) which suggest that the CO solubility in dimethyl carbonate at 20°C and 1 bar is comparable $x \approx 1.2\times10^{-3}$ ($c = 0.014$ mol×L^{-1}, 0.037 wt.-%). Assuming that the solubility of carbon monoxide is proportional to the CO pressure in the range of 0.5 to 3 bar, the initial amount of CO in the reactor can be calculated for the different CO pressures at 20°C.

Monomer reactivity ratio for free-radical CO-ethylene-copolymerization

From the observed pressure drop and the absolute CO amount in the yielded copolymers, the monomer conversion of CO could be estimated (see Supplementary Table 1). Using the measured ethylene concentration at 120 and 140°C to determine the initial amount of ethylene in the reactor, its conversion was calculated to 0.4, 0.5 and 3.5% for entries 4, 6 and 9, respectively. This means $[CO] \approx [CO]_0$ and $[E] \approx [E]_0$ is a valid assumption for reactions at low T, but a rough estimation when $T \geq 140°C$.

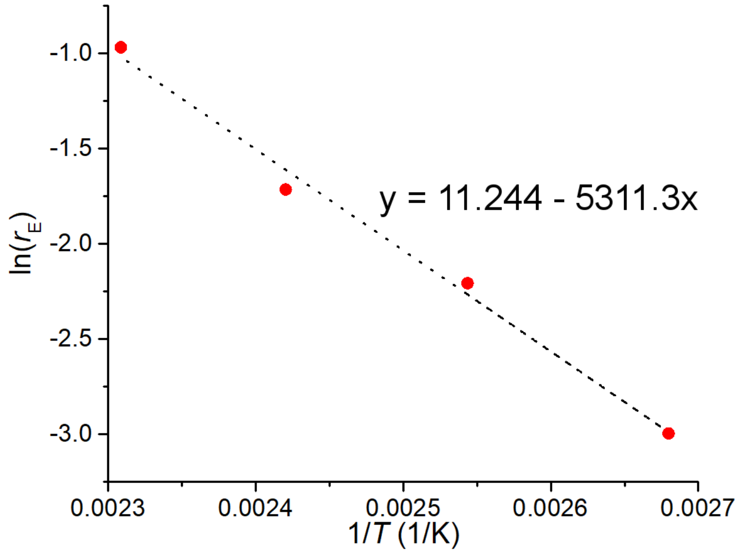

Supplementary Figure 19: Arrhenius plot for the temperature dependence of the monomer reactivity ratio r_E. The difference in global activation energies for $k_{e\text{-}E}$ and $k_{e\text{-}CO}$ is ΔE_a = 44.2 kJ×mol^{-1}.

Monomer reactivity ratio for catalytic CO-ethylene-copolymerization

For comparison, the reactivity ratio of ethylene and carbon monoxide r_E can be estimated from data on catalytic copolymerizations assuming same simplifications as already discussed for the free-radical copolymerization. Luo *et al.* synthesized low-molecular weight linear polyketones with $[CO]_{poly}$ < 10 mol-% at 20.7 bar and 90 to 110°C with an *in situ* generated catalyst from $Pd(OAc)_2$ and 2-[di(2-anisyl)phosphino]benzenesulfonic acid in dichloromethane (Supplementary Table 2).[13] Note that $[CO] \approx [CO]_0$ is a rougher estimation in this case, because CO conversions are typically in the range of 20-40 %.

The solubility of ethylene in dichloromethane (ε = 8.93) at 100°C and 20 bar was estimated to $x \approx$ 0.1 from known solubility data of other chlorinated hydrocarbons:[9]
x = 0.134 (in tetrachloromethane with ε = 2.24, 20 bar, 60°C)
x = 0.124 (in chloroform with ε = 4.81, 20.3 bar, 100°C)
x = 0.116 (in 1,2-dichloroethane with ε = 10.36, 17.8 bar, 60°C)

The solubility of carbon monoxide in dichloromethane at 100°C and 1–2 bar was estimated to $x \approx$ 1-2 10^{-3} from known solubility data of similar solvents:[10]
$x = 8.586\times10^{-4}$ (in tetrachloromethane, 1 bar, 20°C)
$x = 9.327\times10^{-4}$ (in tetrachloromethane, 1 bar, 60°C)
$x = 6.44\times10^{-4}$ (in chloroform, 1 bar, 25°C)
$x = 4.72\times10^{-4}$ (in 1,2-dichloroethane, 1 bar, 20°C)

Extrapolation of the densities for dichloromethane at 10 and 100 bar and temperatures between 20 and 40°C determined by Chorazewski *et al.*[14] results in a density of 1.18 $g\times mL^{-1}$ of the solvent at reaction conditions (neglecting the impact of dissolved ethylene). This allows for calculation of the initial concentrations of the monomers $[ethylene]_0$ and $[CO]_0$ and the monomer reactivity ratio (see Supplementary Table 3).

$[ethylene]_0 = 1.53\ mol\times L^{-1}$ (in dichloromethane, 20 bar, 90-110°C)
$[CO]_0 = 0.0138\ mol\times L^{-1}$ (in dichloromethane, 1 bar, 90-110°C)

Synthesis and characterization of terpolymers of ethylene, CO and difunctional monomers

EGDMA is incorporated more efficiently than DVA due to the higher reactivity of methacrylates compared to vinyl esters in free-radical copolymerization with ethylene (see Supplementary Table 4). That is, rather high amounts of EGDMA (up to 2.5 mol-%) can be incorporated with low concentrations (0.1 M) in the initial reaction mixture. There is no evidence for unreacted methacrylate in neither IR nor NMR spectra which means EGDMA is always incorporated with both functionalities. However, M_W increases only from 10 000 to 21 000 g×mol^{-1} and M_W/M_n from 1.7 to 2.2. This suggests that the distribution of difunctional comonomer is rather inhomogeneous in the sample. The high reactivity of EGDMA compared to ethylene leads to a pronounced compositional drift during the polymerization, meaning methacrylate-rich polymer is formed at an early stage and less EGDMA is incorporated later. However DVA is incorporated less (1 mol-% in terpolymerization in the presence of 0.1 M), its effect on the molecular weight distribution is more pronounced. We ascribe this to its more homogeneous distribution in the sample due to similar reactivities of vinyl esters and ethylene. Both IR and NMR show that 20-30% of vinyl groups in incorporated DVA remain unreacted.

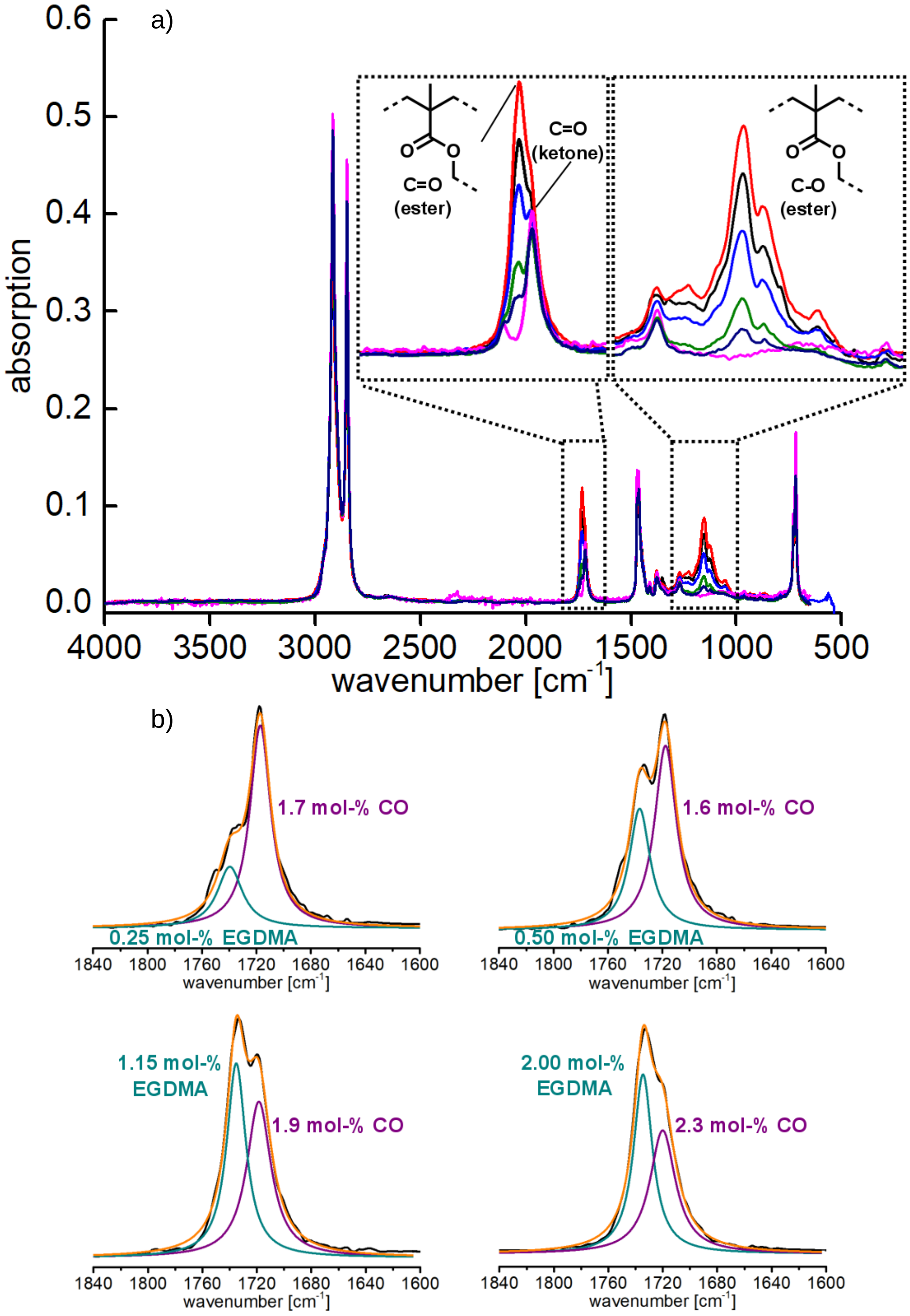

Supplementary Figure 20: ATR-IR spectra of terpolymers of ethylene, CO and EGDMA. Synthesis in DMC with different concentrations of difunctional comonomer (a). IR band deconvolution for the determination of ester and ketone group concentration in obtained terpolymers (b).

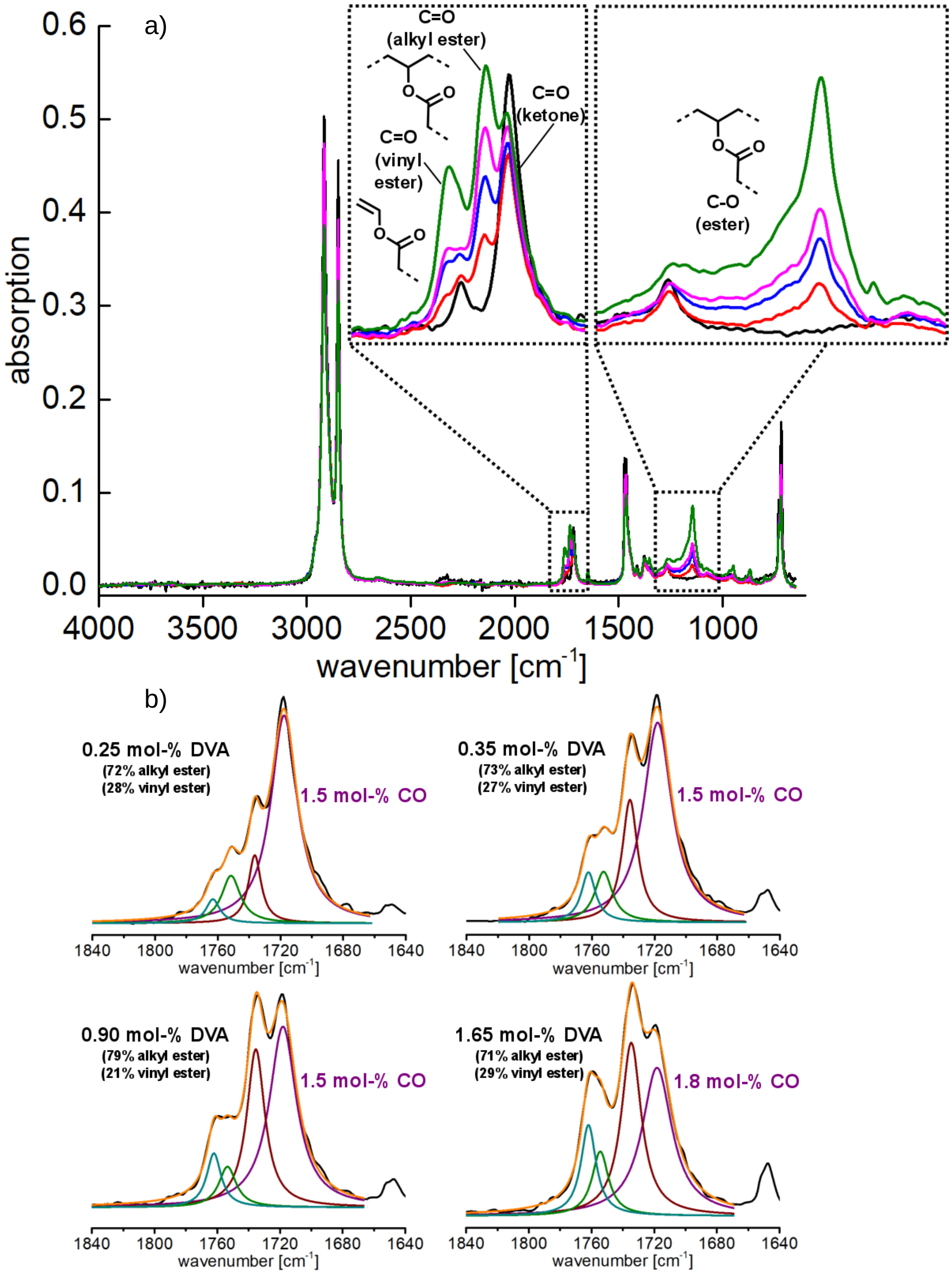

Supplementary Figure 21: ATR-IR spectra of terpolymers of ethylene, CO and DVA. Synthesis in DMC with different concentrations of difunctional comonomer (a). IR band deconvolution for the determination of ester (alkyl+vinyl) and ketone group concentration in obtained terpolymers (b). Green band: Residual DMC (after 1 day under vacuum at 60°C).

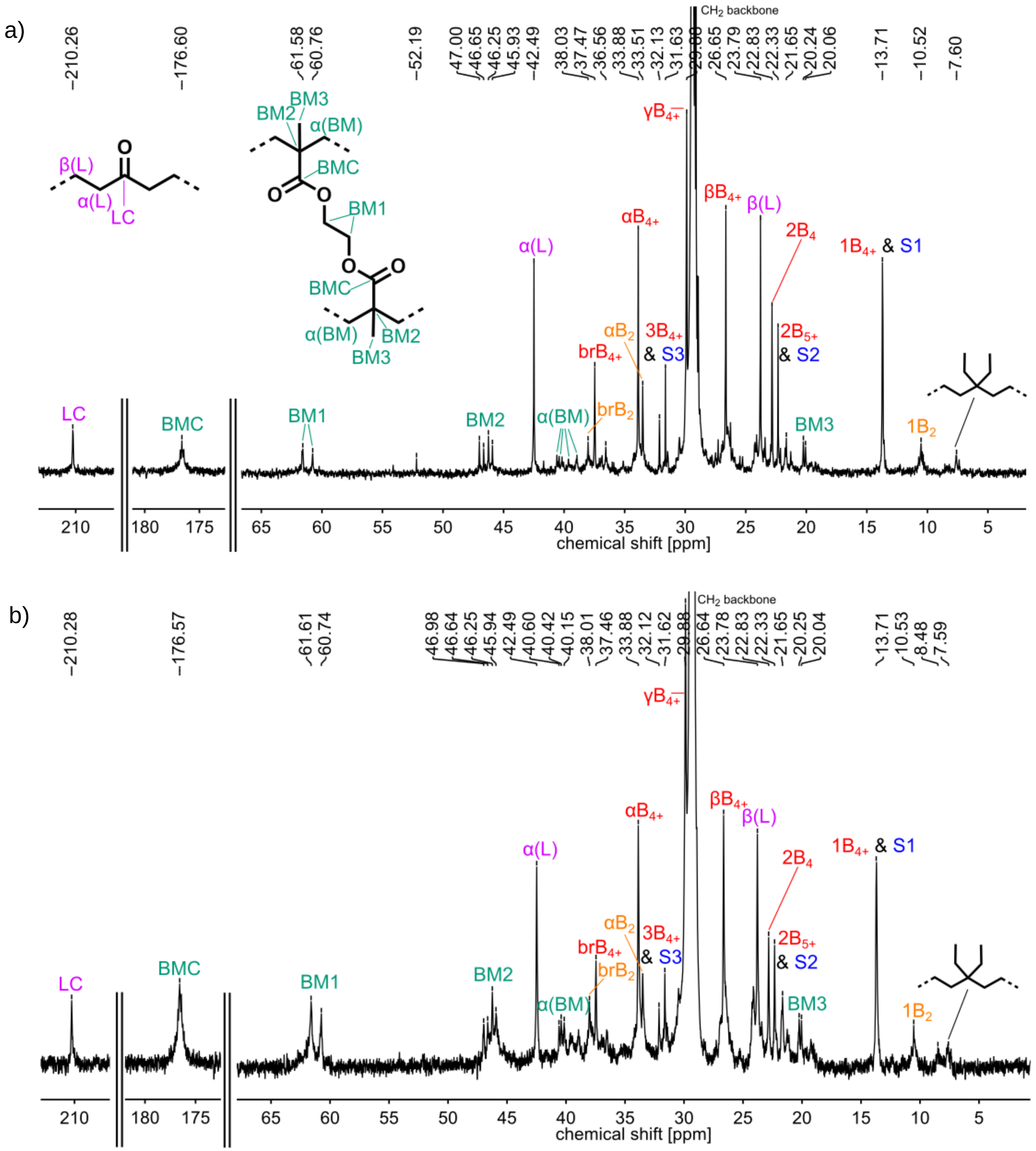

Supplementary Figure 22: ^{13}C NMR spectra of terpolymers. Inverse-gated spectra at 100°C in 1,1,2,2-tetrachloroethane-*d*$_2$ of terpolymers of ethylene, CO and EGDMA synthesized in DMC. a: Terpolymer with 1.4 mol-% CO and 1.3 mol-% EGDMA. b: Terpolymer with 1.8 mol-% CO and 2.5 mol-% EGDMA.

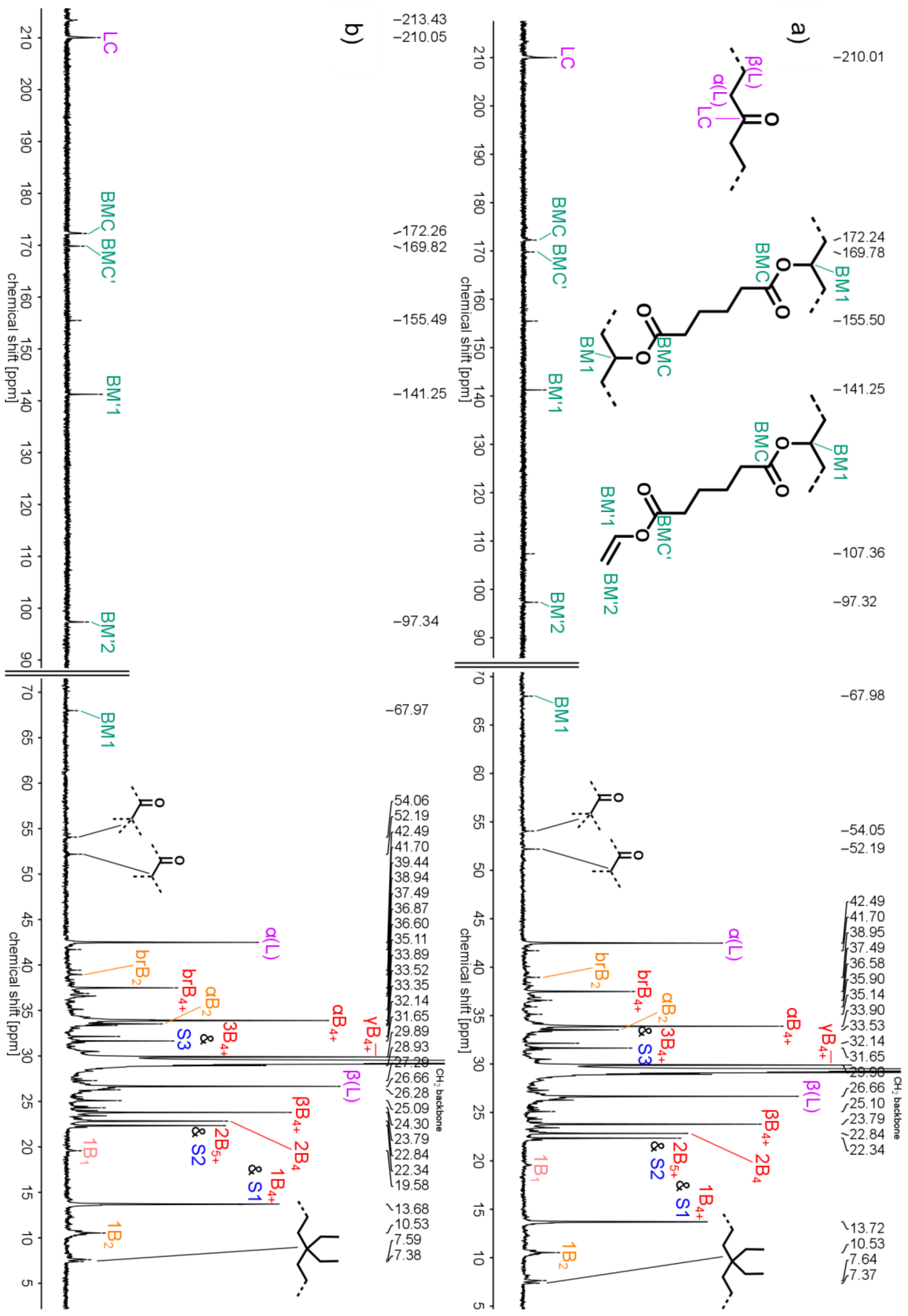

Supplementary Figure 23: ^{13}C NMR spectra of terpolymers. Inverse-gated spectra at 100°C in 1,1,2,2-tetrachloroethane-*d*$_2$ of terpolymers of ethylene, CO and DVA synthesized in DMC. a: Terpolymer with 1.1 mol-% CO and 0.2 mol-% DVA. b: Terpolymer with 1.1 mol-% CO and 0.35 mol-% DVA.

Specimen preparation and stress-strain tests

Specimens of both polyketones and terpolymers were prepared by injection molding (Supplementary Figures 24, 25). Melts of polyketones have honey-like viscosities at temperatures applied for injection molding (150-190°C). In contrast, melts of DVA-containing terpolymers were rubbery, sticky and did not flow on an observable timescale without applying pressure. ATR-IR spectra of the polymer samples before and after injection molding were unchanged, excluding significant (partial) oxidation of keto groups or PE segments by oxygen under injection molding conditions. Tensile tests (Supplementary Figures 26, 27 and Supplementary Table 5) were carried out as previously described.

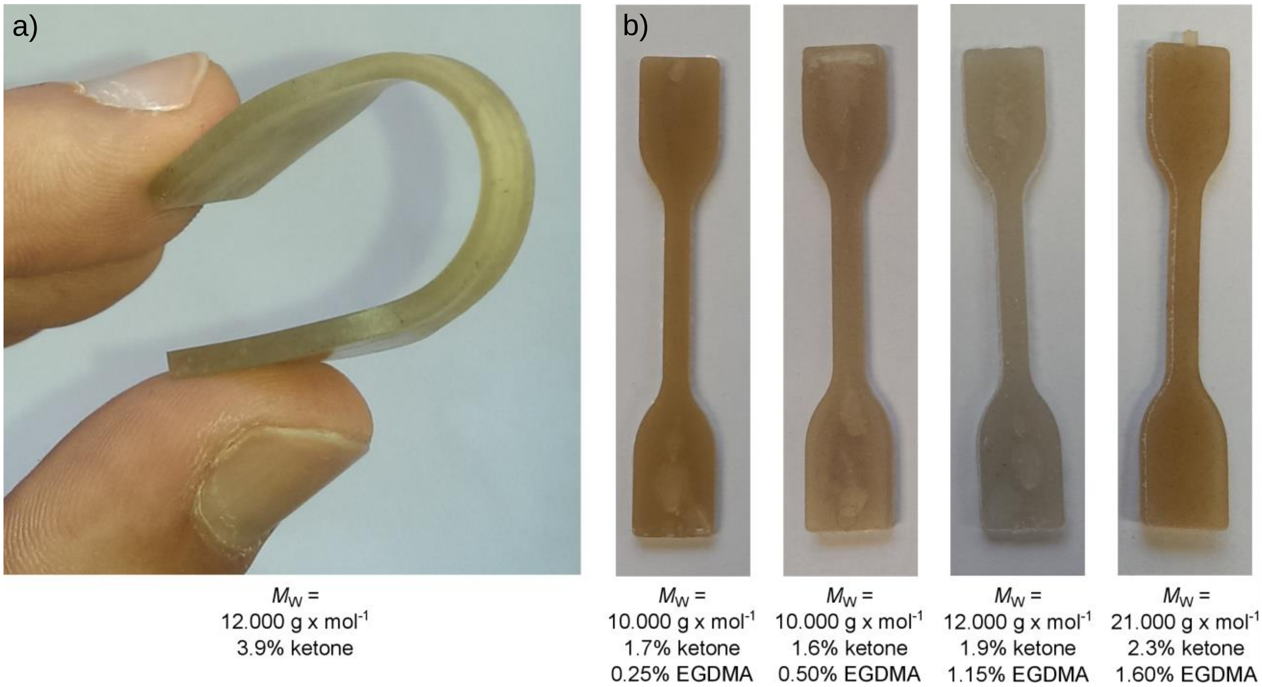

Supplementary Figure 24: Appearance of polyketone/terpolymer specimens. Deformation of a dogbone-shaped specimen prepared by injection molding of a polyketone without difunctional comonomer (a). Appearance of dogbone specimens prepared from terpolymers with different contents of carbon monoxide and EGDMA (b).

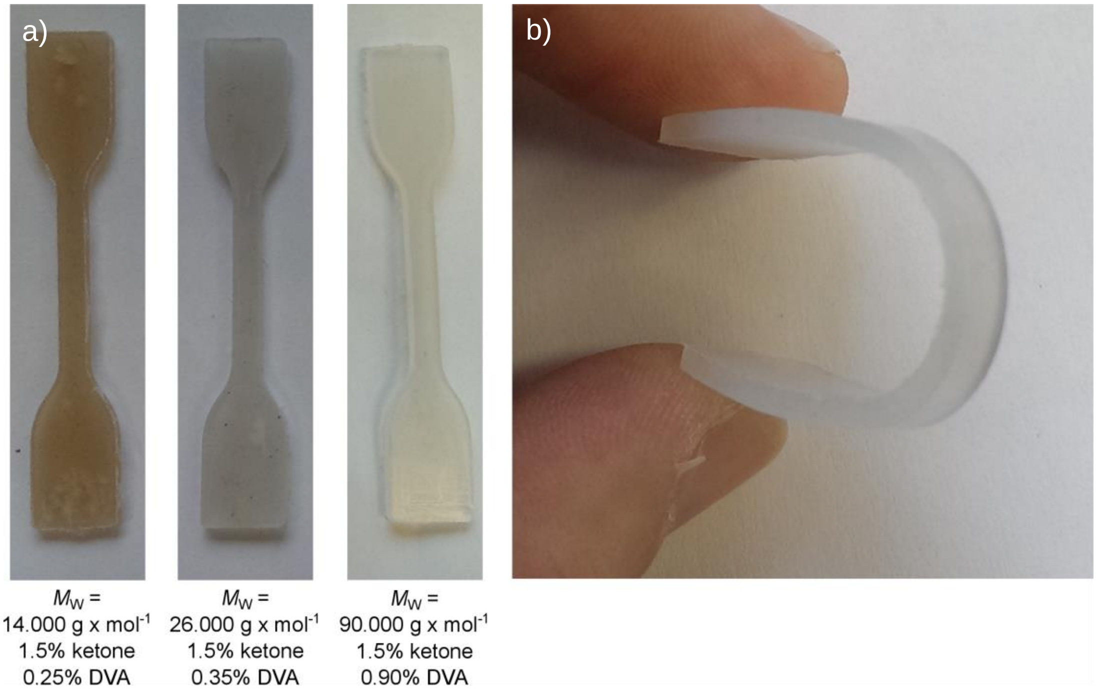

Supplementary Figure 25: Appearance of terpolymer specimens. Appearance of dogbone specimens prepared by injection molding of terpolymers with different contents of DVA (a). Deformation of a dogbone-shaped specimen of a DVA-containing terpolymer (b).

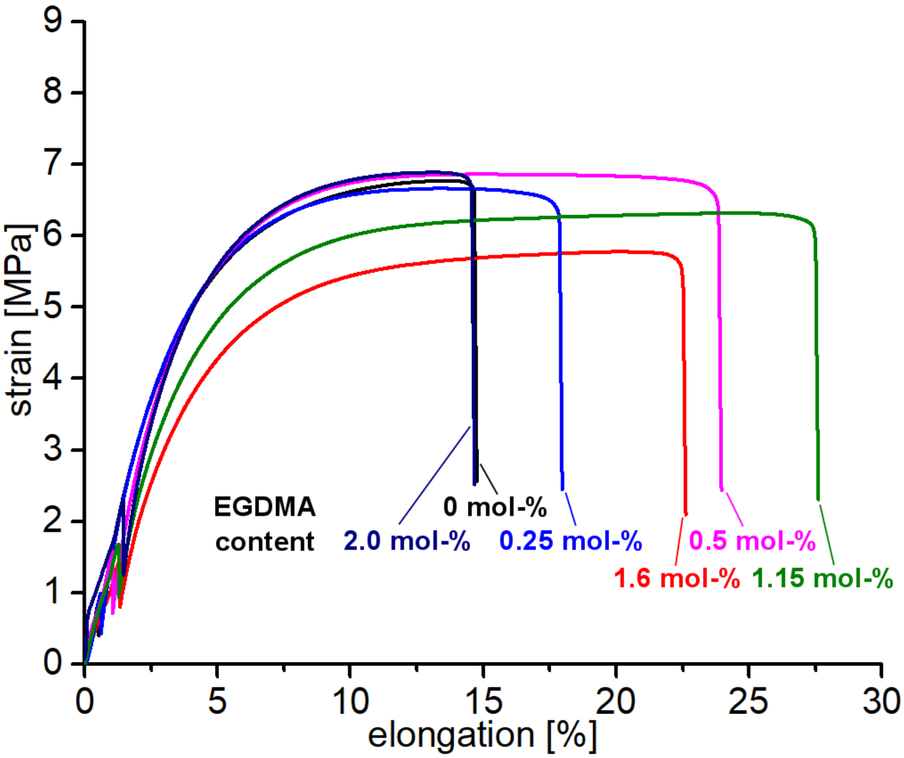

Supplementary Figure 26: Stress-strain curves of terpolymers of ethylene, carbon monoxide and EGDMA (75 × 12.5 × 2 mm^3 dogbone specimens, 5 mm×min^{-1} crosshead speed, according to ISO 527-2, type 5A).

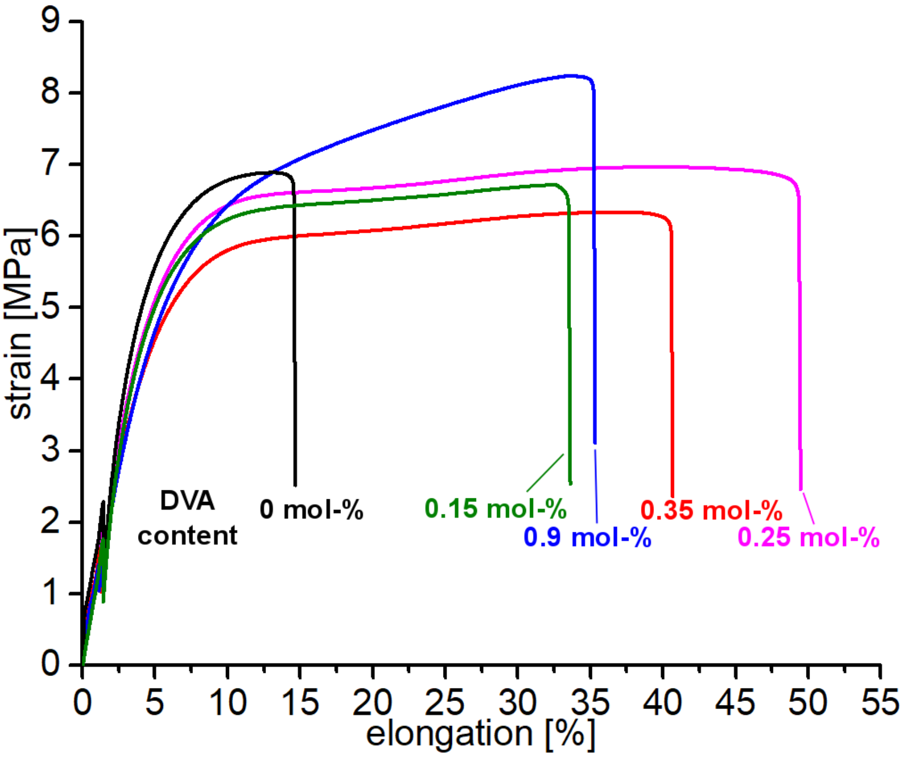

Supplementary Figure 27: Stress-strain curves of terpolymers of ethylene, carbon monoxide and DVA (75 × 12.5 × 2 mm^3 dogbone specimens, 5 mm×min^{-1} crosshead speed, according to ISO 527-2, type 5A).

Weathering study

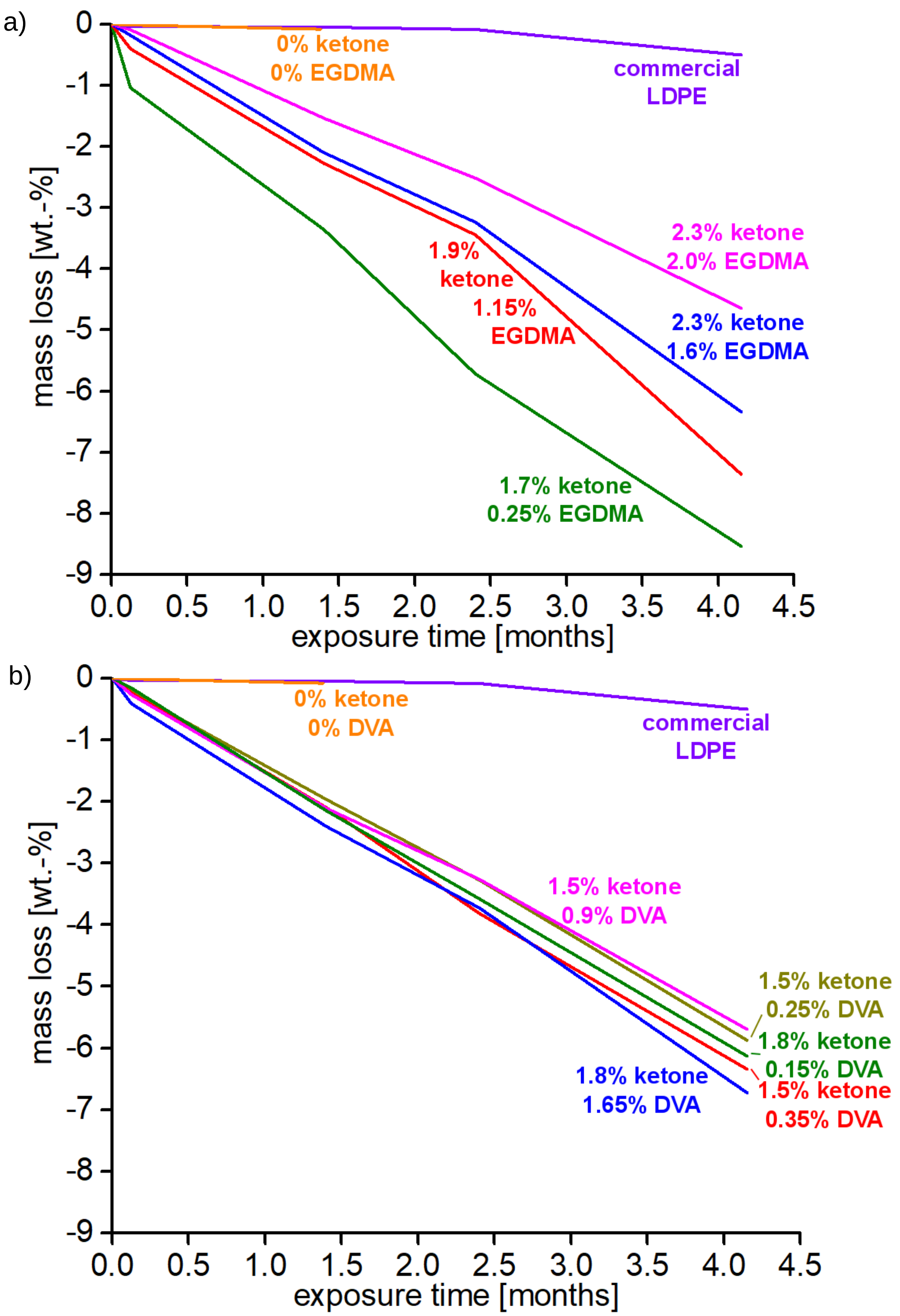

Supplementary Figure 28: Results of specimen weathering studies. Mass loss over time under UV-irradiation for specimens of commercial LDPE, terpolymers of ethylene, CO and EGDMA (a) or DVA (b) and an ethylene homopolymer comparative sample. Weathering conditions: 30 W×m^{-2} light intensity, optical filter for daylight simulation on sea level, samples are permanently located in distilled water of 35°C. 1 simulated month ≈ 4 months under natural sunlight in Southern Europe.

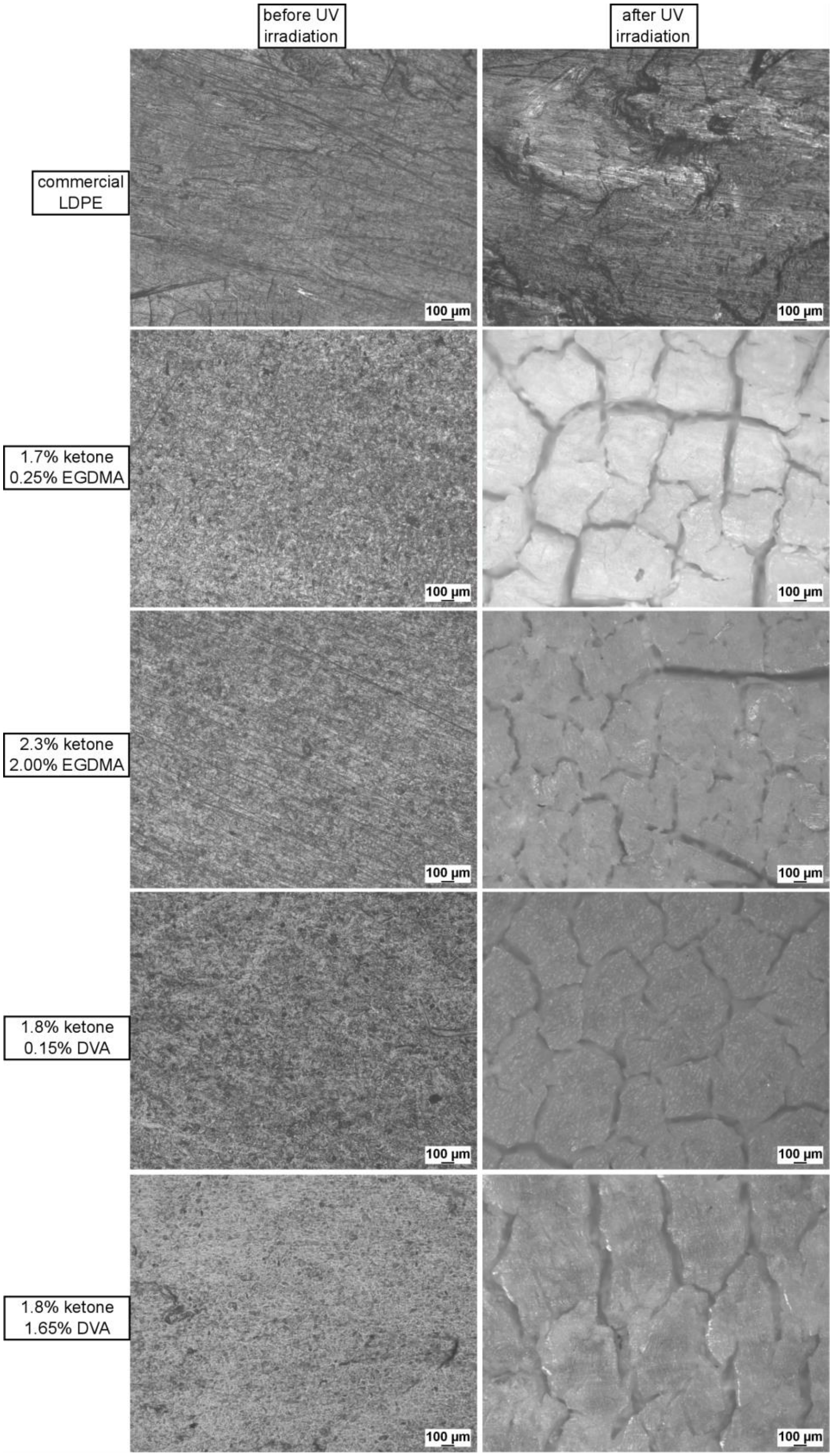

Supplementary Figure 29: Specimen surface analysis. Reflected light microscopy images of specimen surfaces before and after 4 months of UV-irradiation (30 W m^2 light intensity, samples flooded with distilled water of 35°C). As opposed to unfunctionalized LDPE, surfaces of ketone-modified polymers exhibit 100-500 µm deep cracks and become uneven due to degradation of the surface.

TEM images of polyketone nanoparticles (aqueous polymerizations)

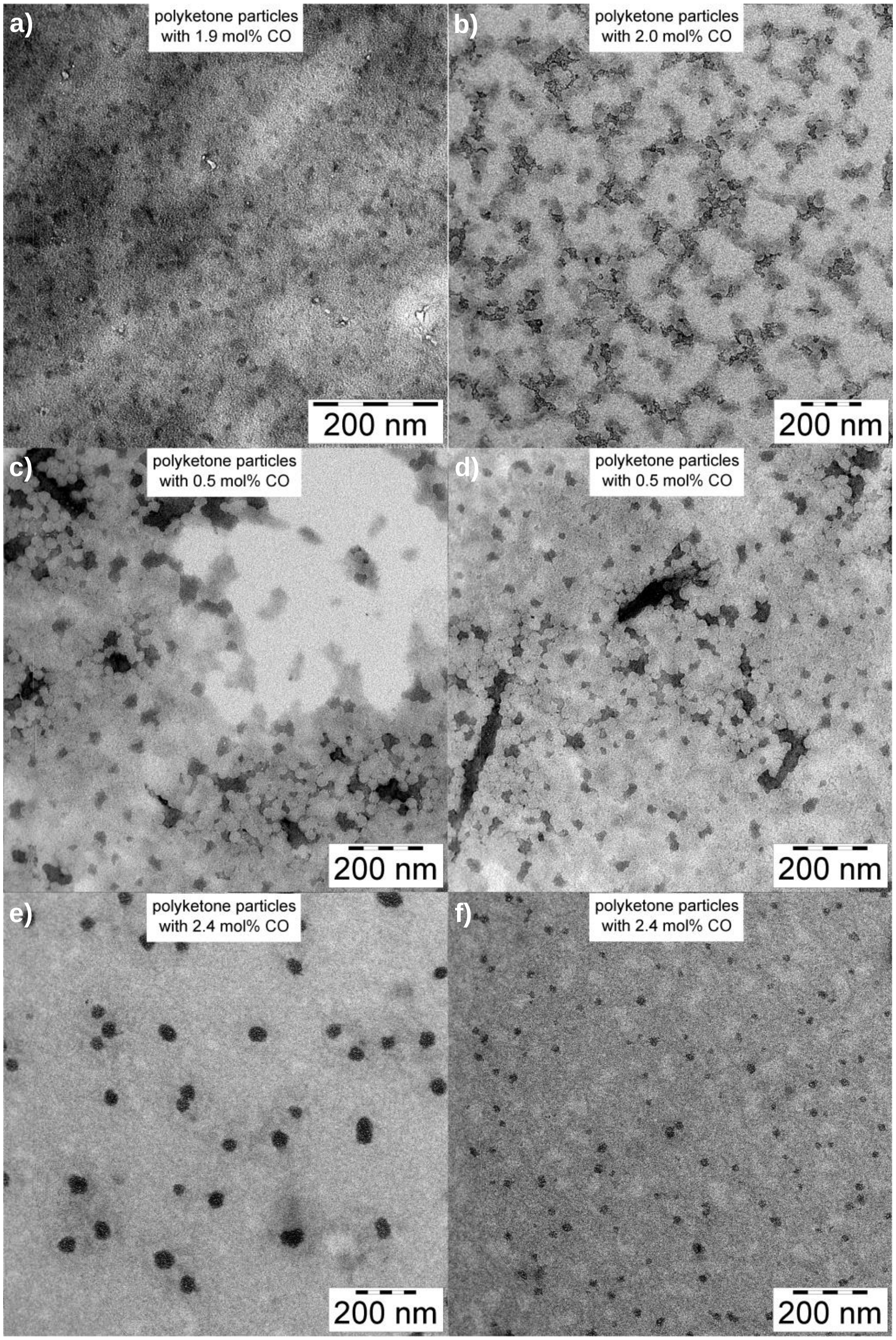

Supplementary Figure 30: TEM images of polyketone nanoparticles. Samples with different CO contents synthesized via surfactant-free dispersion copolymerization (a,b) and dispersion copolymerization in the presence of SDS (c,d,e,f).

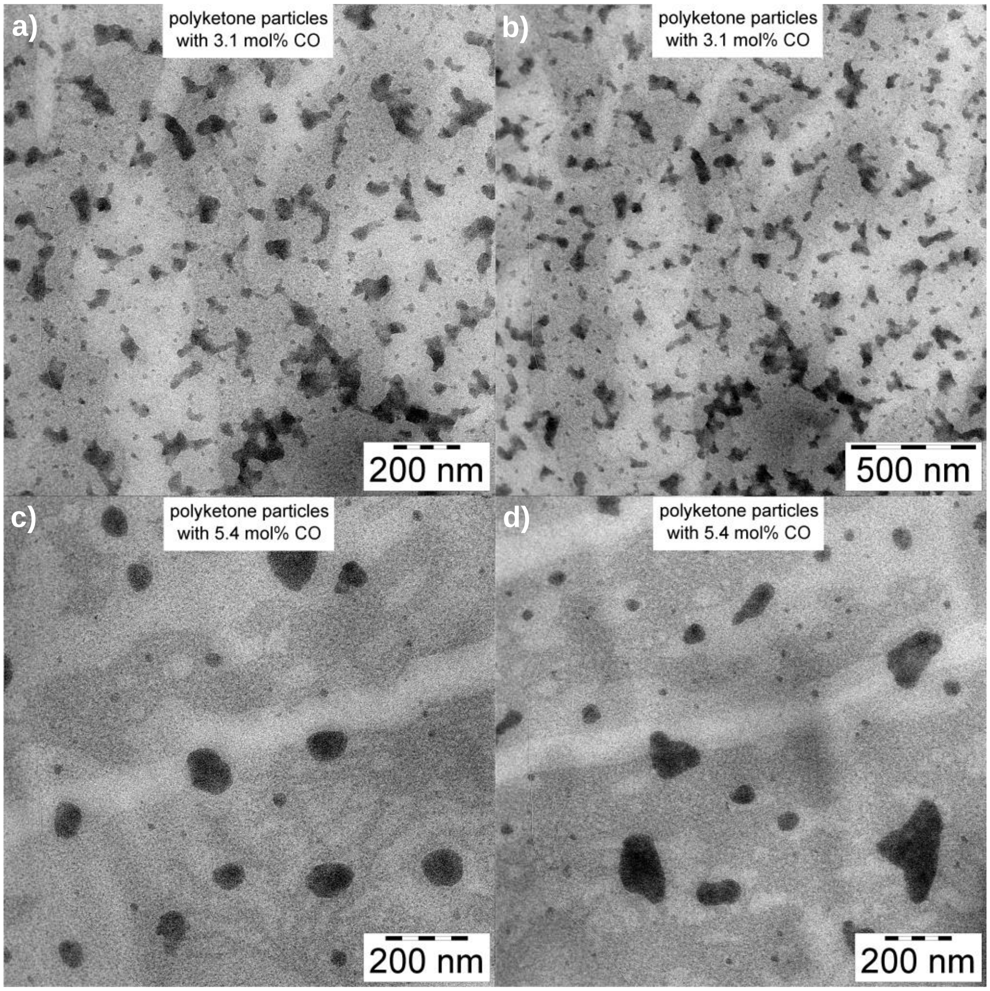

Supplementary Figure 31: TEM images of polyketone nanoparticles. Samples with different CO contents synthesized via dispersion copolymerization in the presence of SDS. a) 3.1 mol-% CO. b) 5.4 mol-% CO.

NMR spectra of copolymers (aqueous polymerizations)

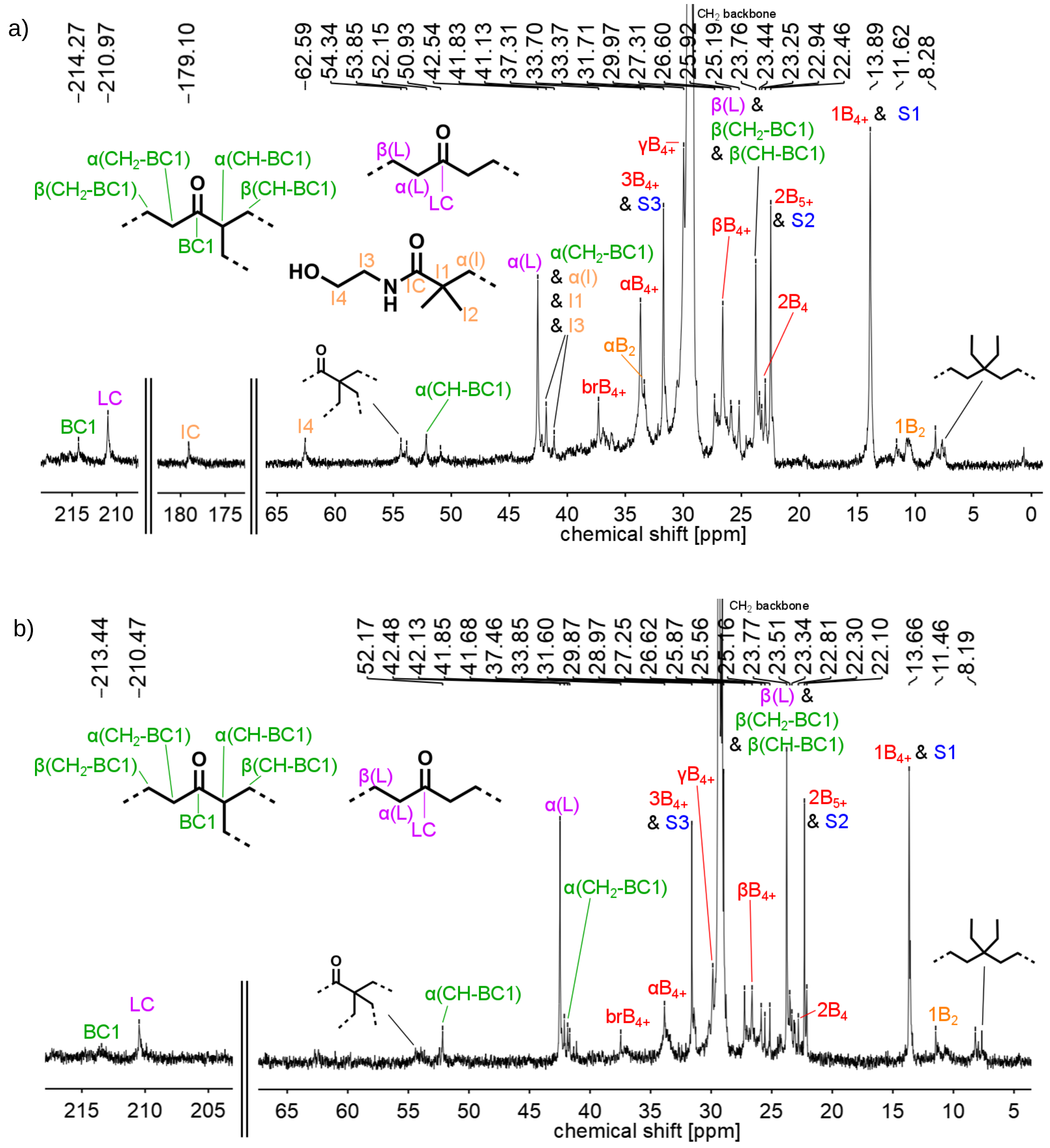

Supplementary Figure 32: ^{13}C NMR spectra of polyketones. Inverse-gated spectra at 100°C in 1,1,2,2-tetrachloroethane-d_2 of polyketones synthesized in water with CO contents of 2.3 (a) and 7.5 (b) mol-%, respectively. Signals of branches or backbone methylene groups in close proximity to branches were assigned according to [2]. LC: linear carbonyl. BC: α-branched carbonyl.

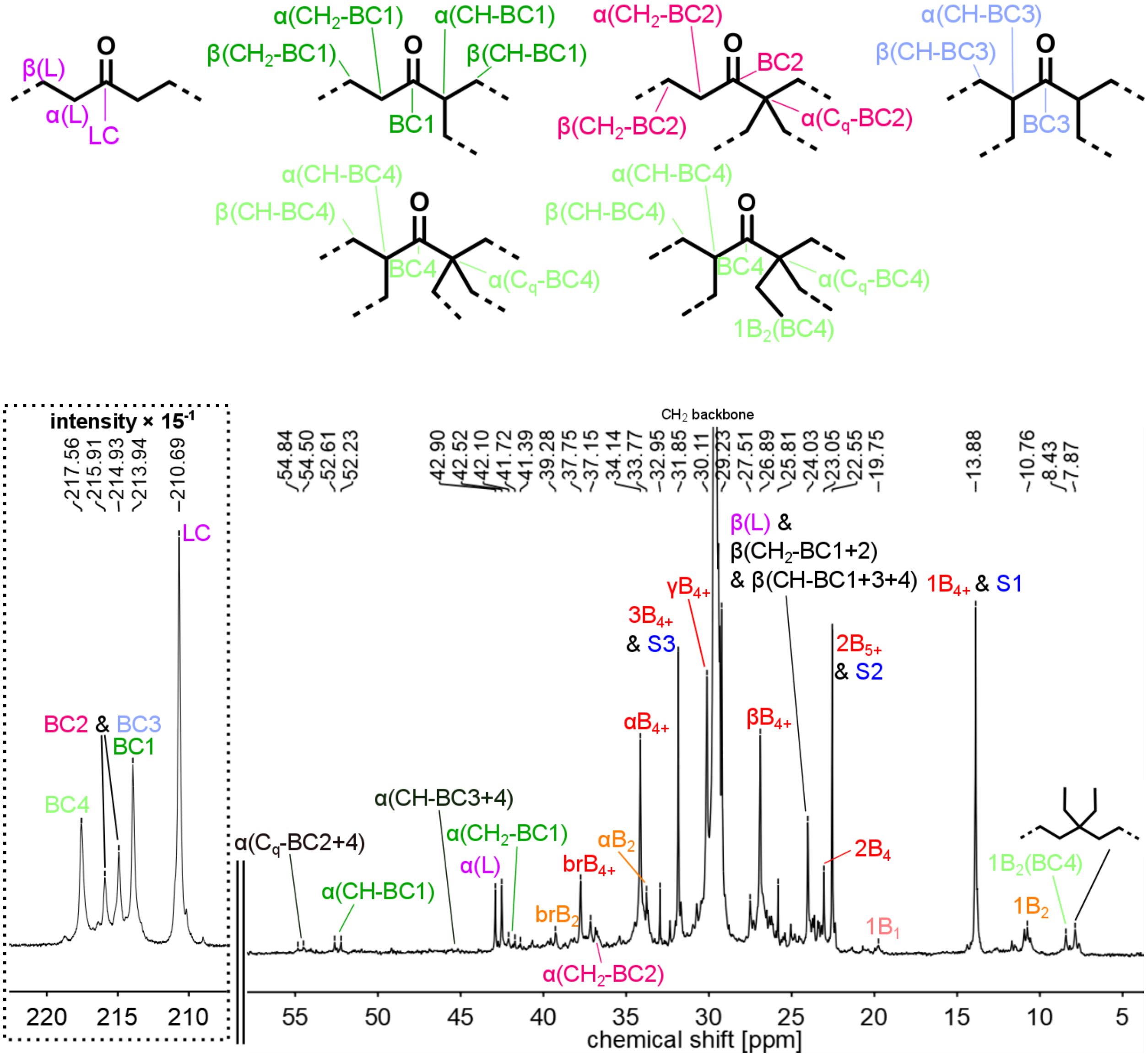

Supplementary Figure 33: ^{13}C NMR spectrum of a ^{13}C-labeled polyketone. Inverse-gated spectrum at 100°C in 1,1,2,2-tetrachloroethane-d_2 of a polyketone synthesized in water with ^{13}CO content of 1.5 mol-%. Peaks of the different ketone groups, α- and β-carbons were assigned in accordance to HMBC, HSQC (Supplementary Figure 34) and spectra of reference compounds. LC: linear carbonyl. BC: α-branched carbonyl.

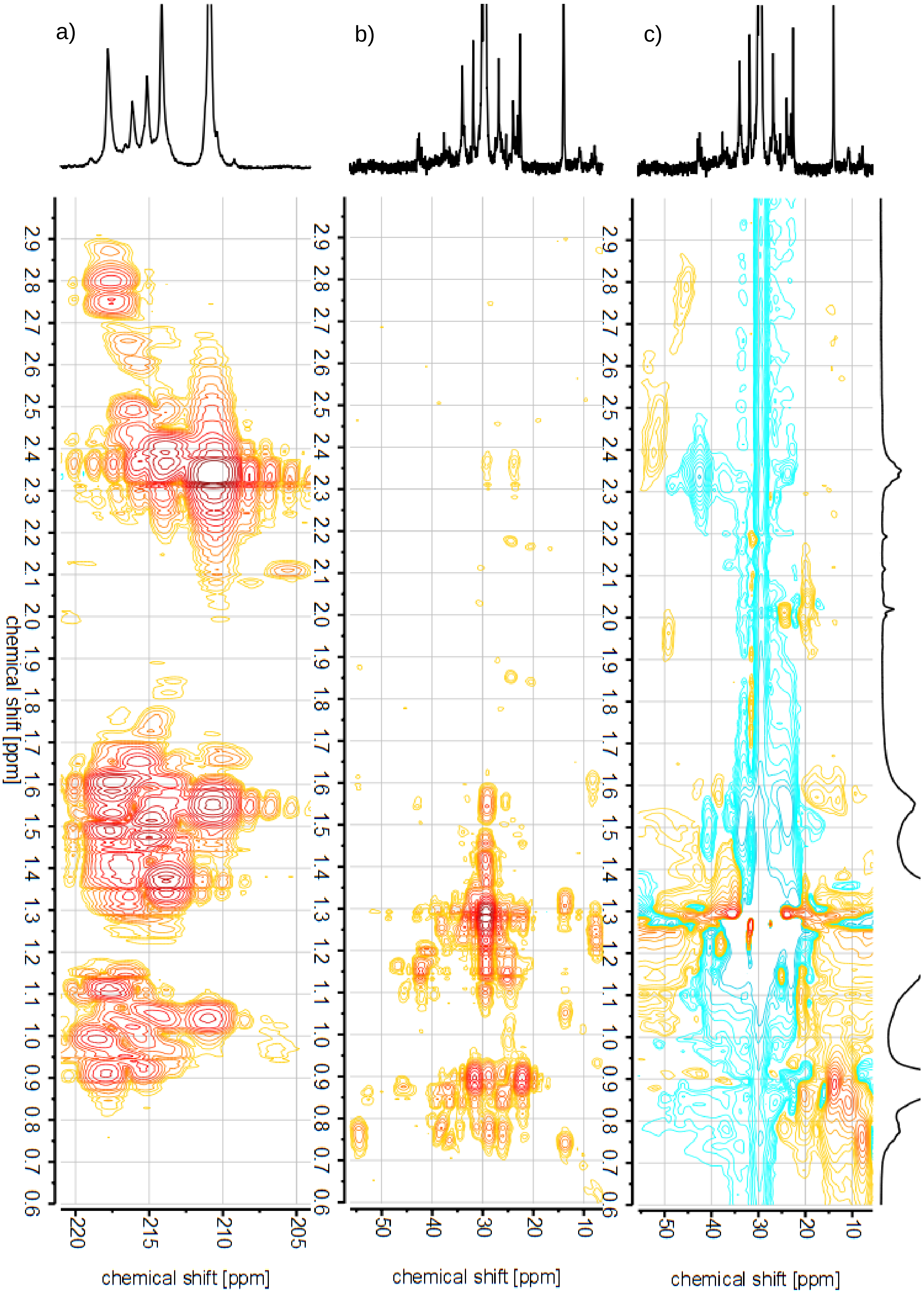

Supplementary Figure 34: 2D NMR spectra of a ^{13}C-labeled polyketone. ^{1}H-^{13}C HMBC of carbonyl carbons (205-220 ppm; a), ^{1}H-^{13}C HMBC (5-55 ppm; b) and ^{1}H-^{13}C HSQC (5-55 ppm; c).

ATR-IR spectra of copolymers (aqueous polymerizations)

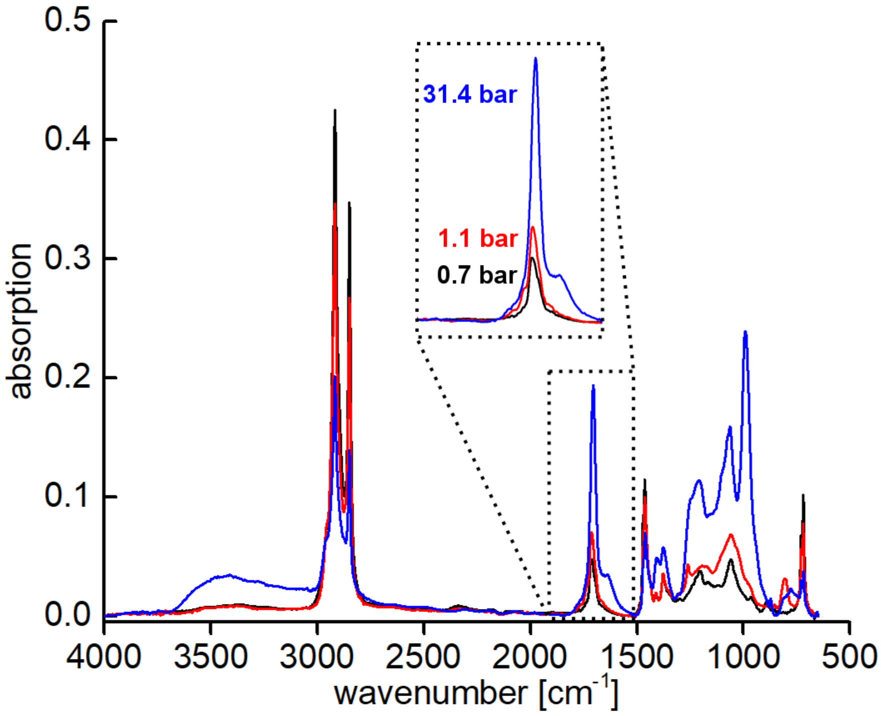

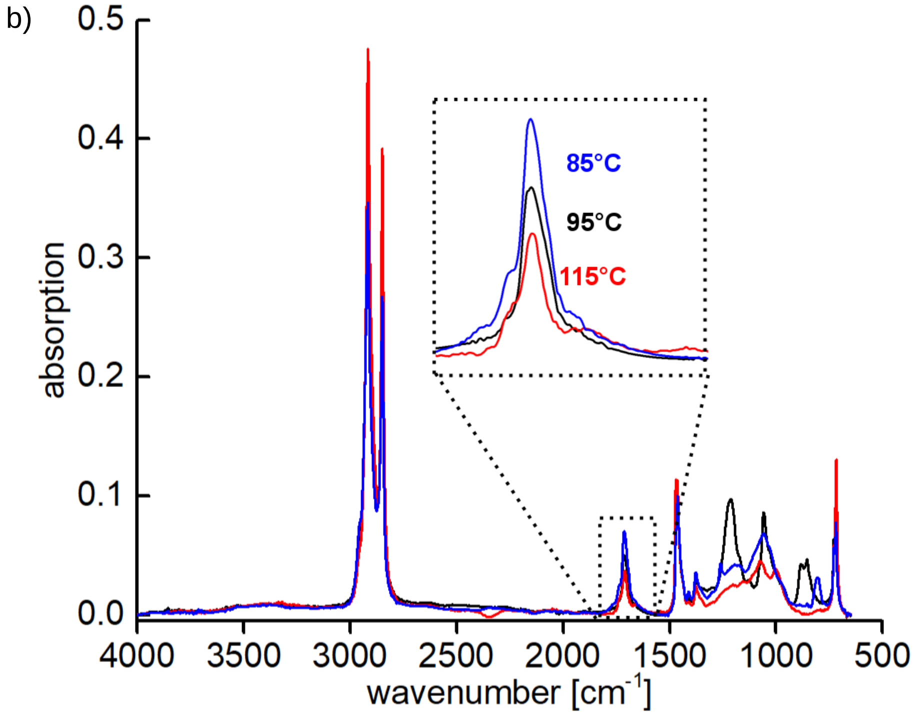

Supplementary Figure 35: ATR-IR spectra of polyketones. Synthesis in water (KPS-initiated) at different initial carbon monoxide pressures (a) and polymerization temperatures (b).

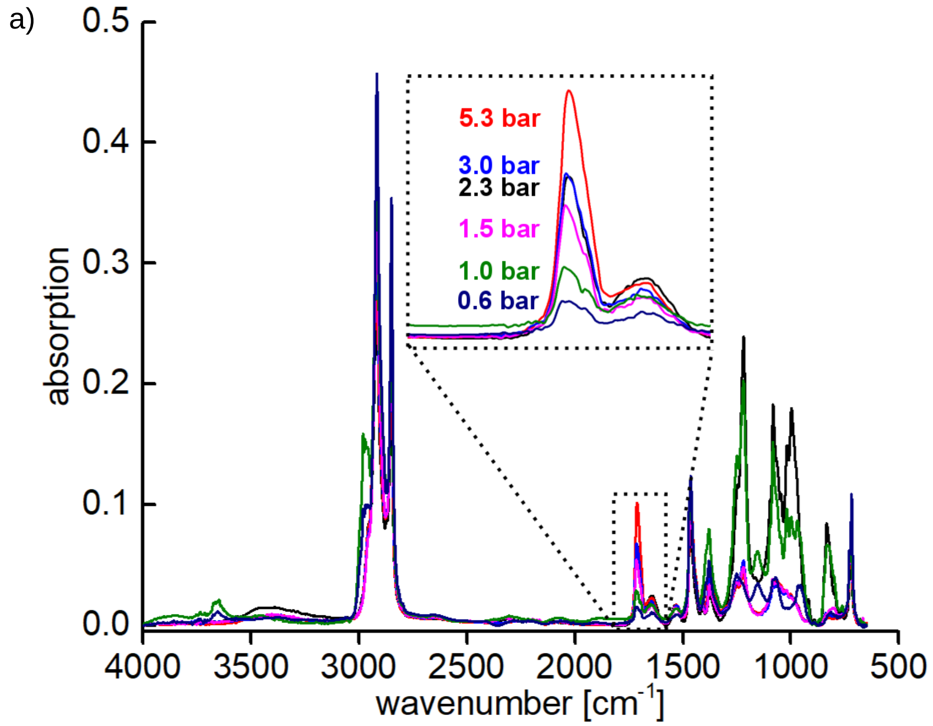

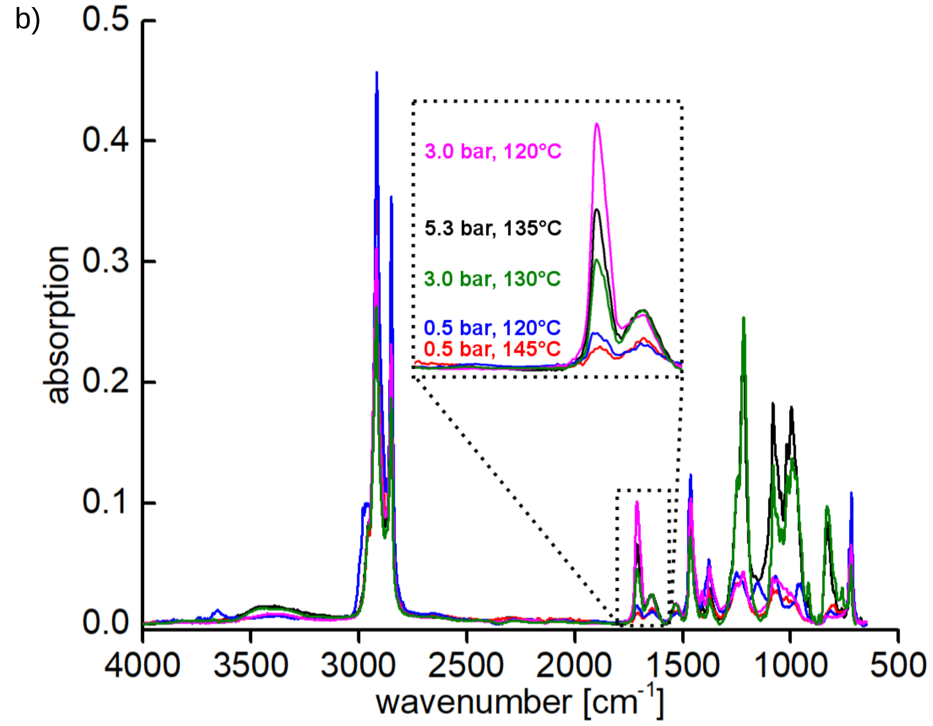

Supplementary Figure 36: ATR-IR spectra of polyketones. Synthesis in water (VA-086-initiated) at different initial carbon monoxide pressures (a) and polymerization temperatures (b).

Analysis of microstructure by IR band deconvolution (aqueous polymerizations)

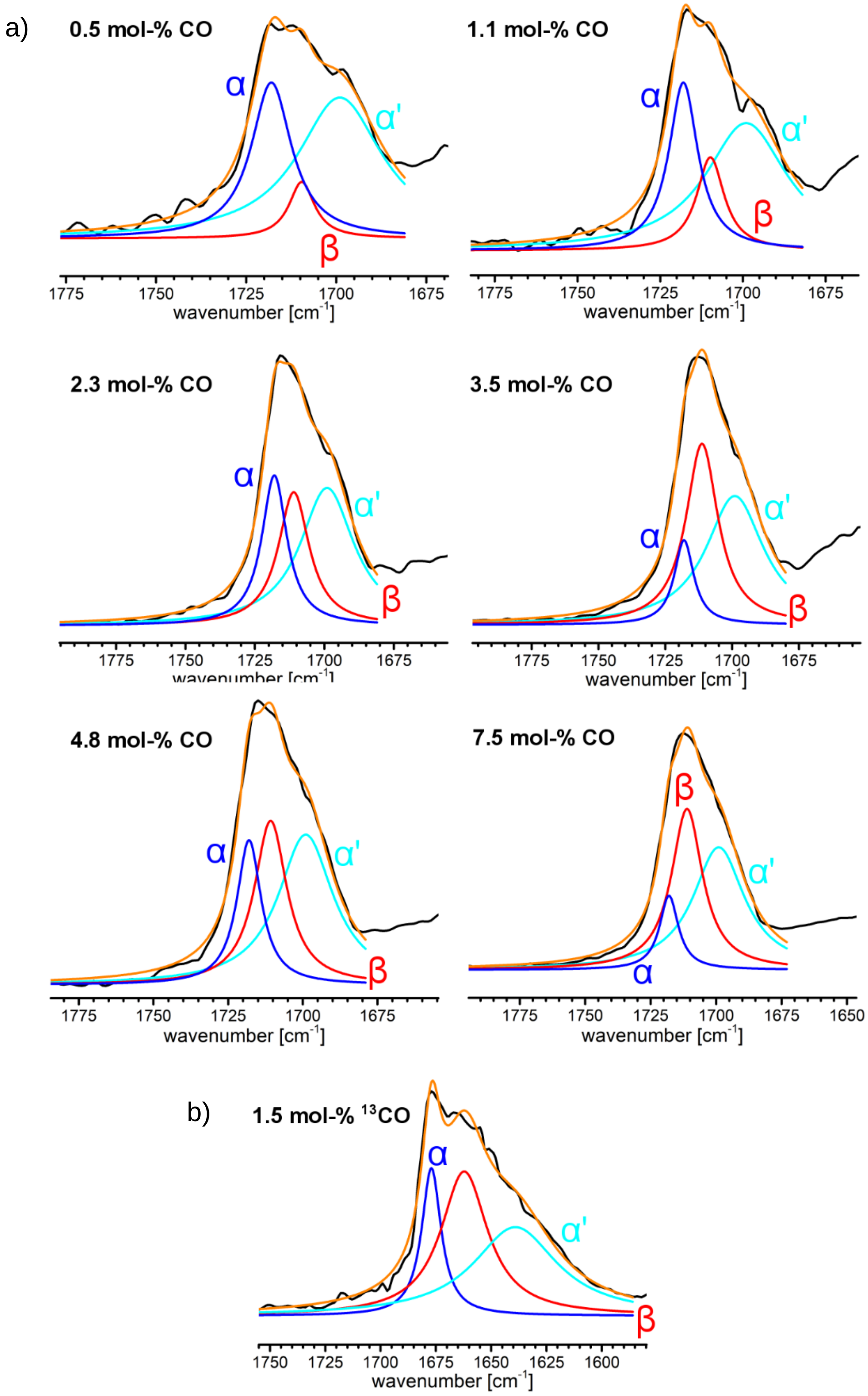

Supplementary Figure 37: ATR-IR band deconvolution. Samples: polyketones from free-radical dispersion copolymerization initiated with VA-086. All three ketone species are shifted equably to lower wavenumbers in spectra of polyketones with ^{13}C-labeled ketone groups (b).

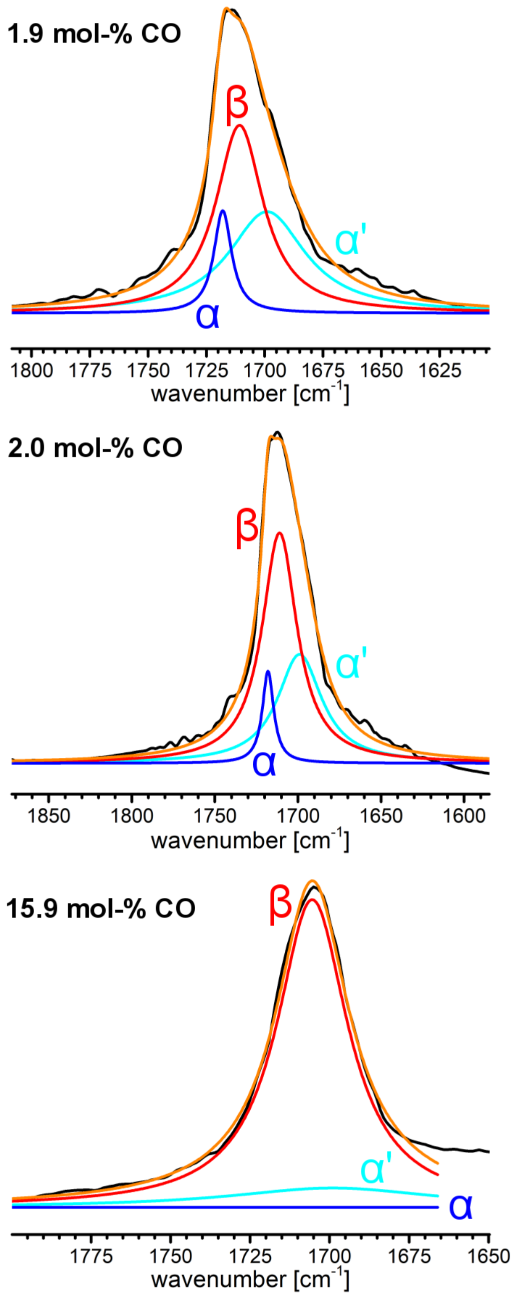

Supplementary Figure 38: ATR-IR band deconvolution. Samples: polyketones from free-radical dispersion copolymerization initiated with potassium persulfate (KPS).

A comparison of the ratios of α and β depending on the C=O content of different polyketones (Supplementary Figure 39) shows that they are similar for solution and dispersion copolymerization and, additionally, independent of the type of initiator used. To compare the spacial distribution of keto groups to a system in which C=O groups are randomly distributed in a PE-matrix, we melted commercial LDPE in the presence of different amounts of 4-heptanone for 8 h. After cooling the mixtures back to r.t., the PE-pellets were washed with ethyl acetate, dried for 1 d under ambient conditions and sliced in two. ATR-IR spectra were recorded on the cut surface. Since 4-heptanone is soluble in PE in these low concentrations, the ketones that diffused inside the PE are randomly distributed. The IR-band of the keto groups of these mixtures could be deconvoluted with the same model previously described. The ratio of α- to β-band shows the same dependence on the C=O content as in polyketones synthesized in free-radical copolymerization (Supplementary Figure 39), which underlines that they are statistical copolymers in which the carbonyl groups are randomly distributed.

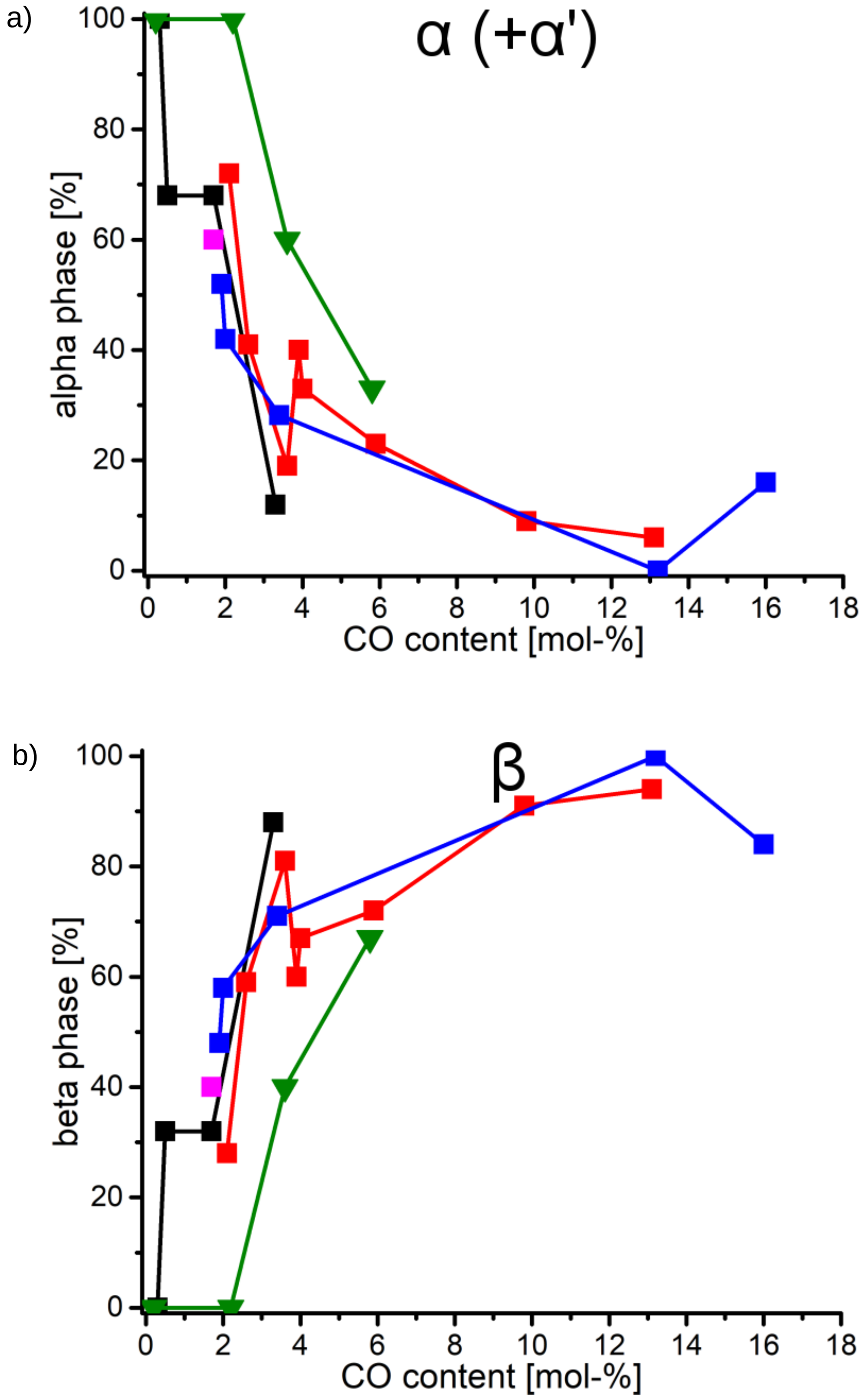

Supplementary Figure 39: Ratio of α- and β-ketones in different samples. Percentage of ketones of a) α- (or α+α' for highly branched polyketones, respectively) and b) β-case for statistical polyketones from free-radical copolymerization (red, blue, magenta squares), long-spaced polyketones from ADMET copolymerization followed by hydrogenation (green triangles) and physical mixtures of 4-heptanone and commercial LDPE (black squares). Polyketones from free-radical copolymerization were synthesized in different media or with different initiators: Dispersion copolymerization in water, KPS-initiated (blue squares); dispersion copolymerization in water, VA-086-initiated (magenta squared); solution copolymerization in DMC (red squares).

DSC traces of copolymers (aqueous polymerizations)

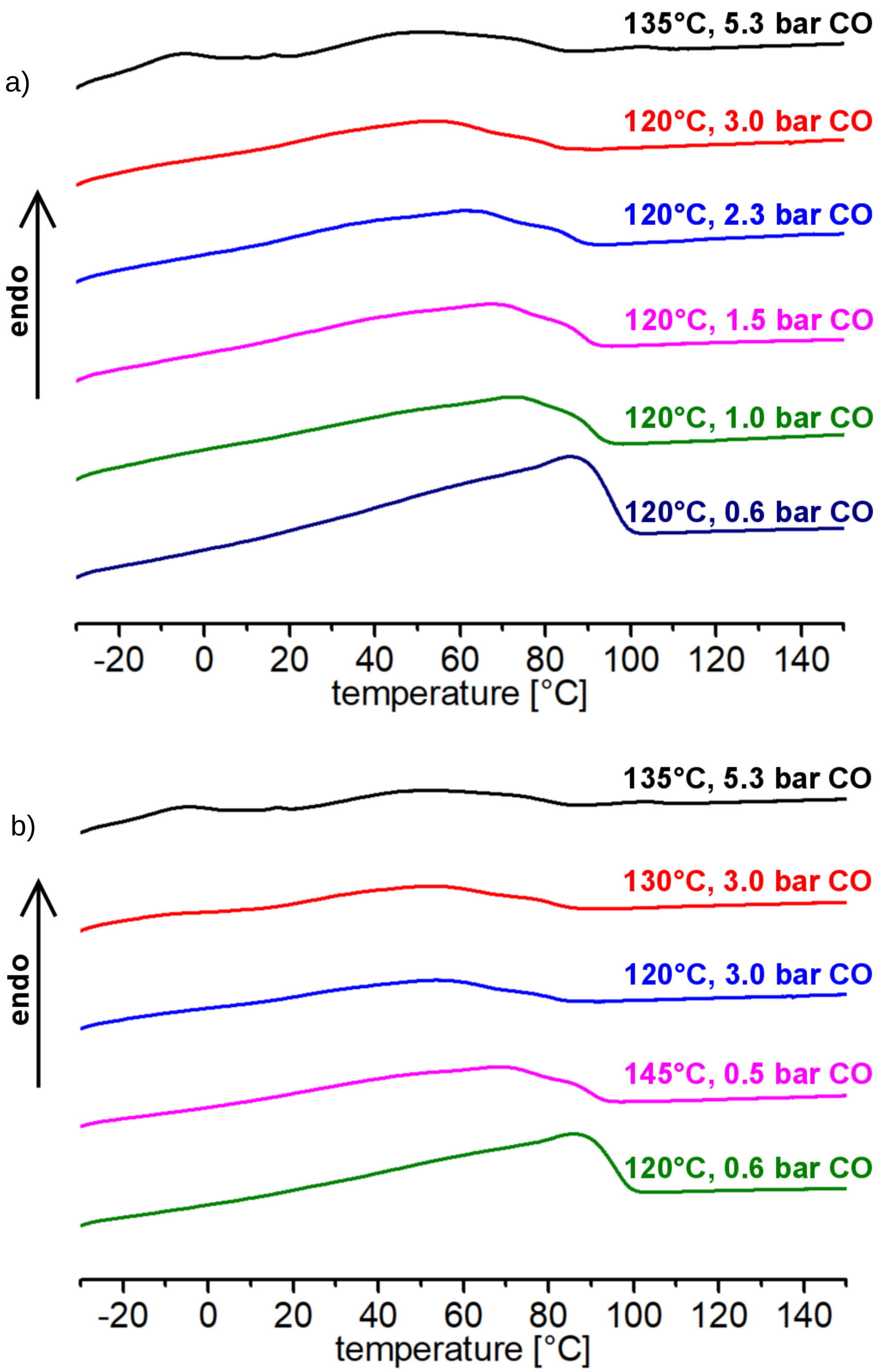

Supplementary Figure 40: DSC data of different polymers. Second DSC heating curves of polyketones synthesized in water at different initial carbon monoxide pressures (a) and different polymerization temperatures (b).

GPC traces of copolymers (aqueous polymerizations)

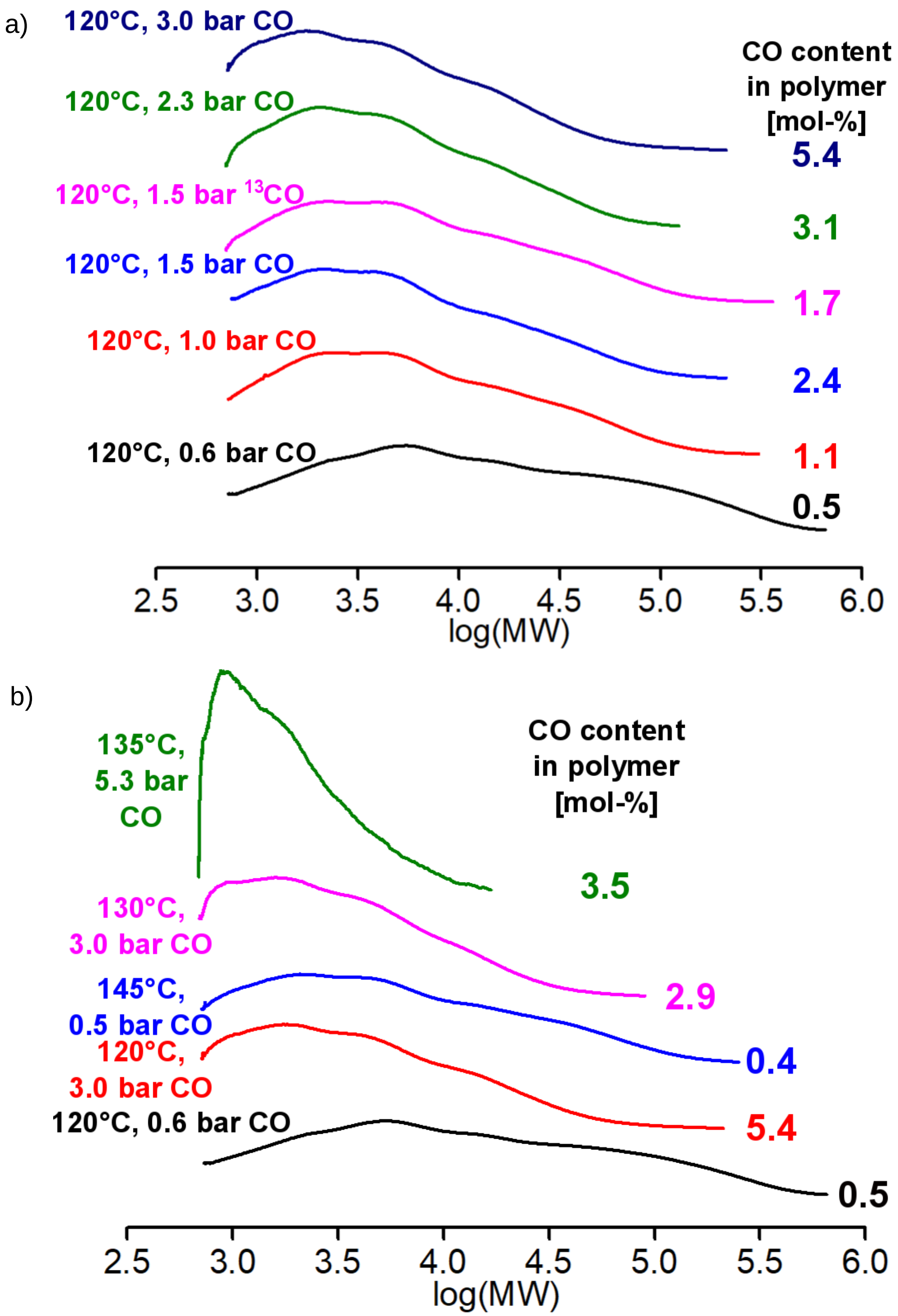

Supplementary Figure 41: GPC data of polymers. Molecular weight distributions of polyketones synthesized in water with different initial carbon monoxide pressures (a) and polymerization temperatures (b).

Effect of CO partial pressure and reaction temperature in different media

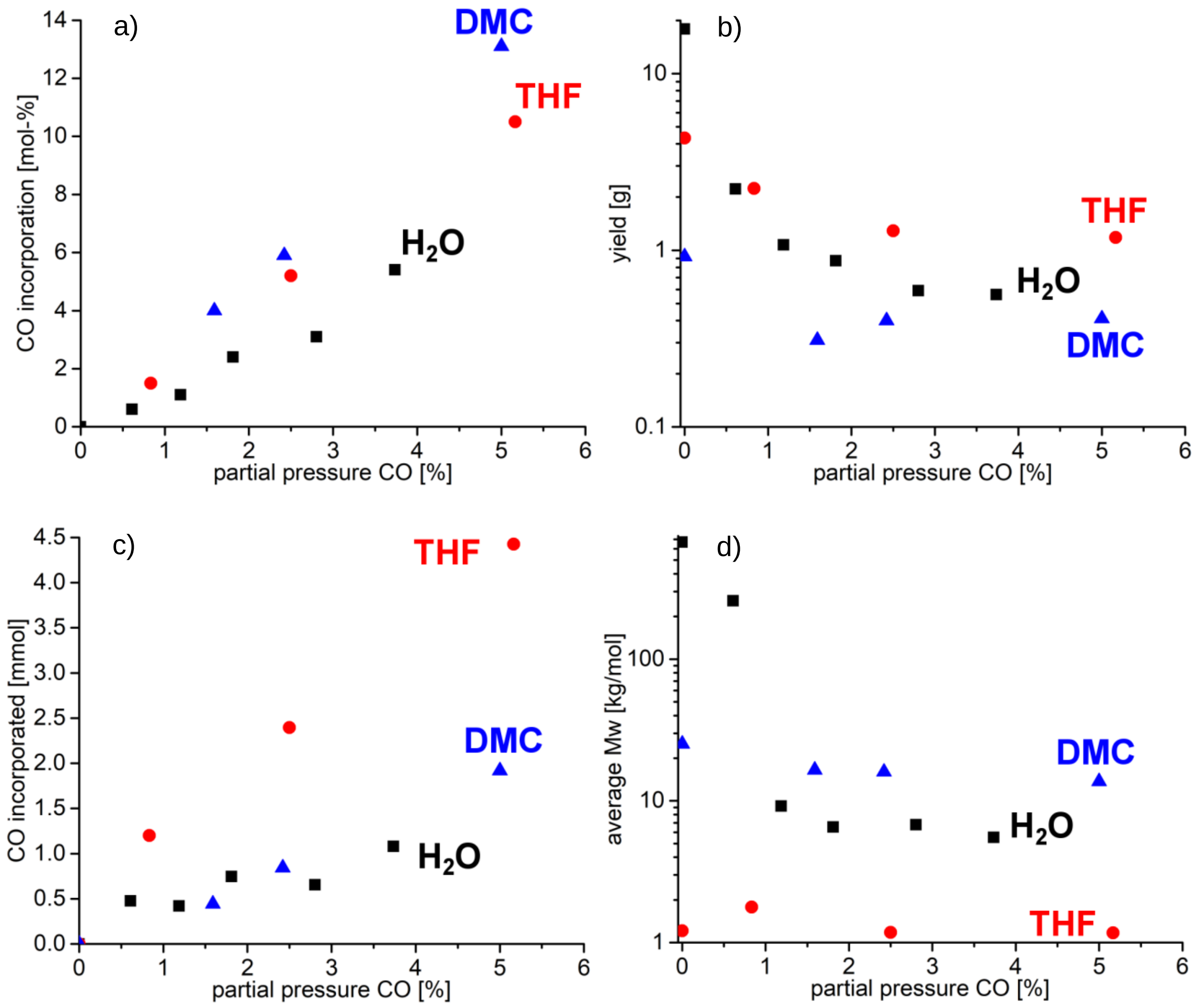

Supplementary Figure 42: Comparison of different reaction media. Effect of partial carbon monoxide pressure adjusted at 20°C on the relative (a) and total CO incorporation (c), the polymer yield (b) and the polymer weight-average molecular weight (d) in different media. Reaction conditions: 120°C, 260-330 bar total pressure, 1 h, 150 mL of water (black squares) or 75 mL of the respective organic solvent (THF: red circles, DMC: blue triangles), 0.3 mmol free-radical initiator (VA-086 in water, di-*tert*-butyl peroxide in organic solvents), 1000 rpm stirring rate with a pitched blade stirrer.

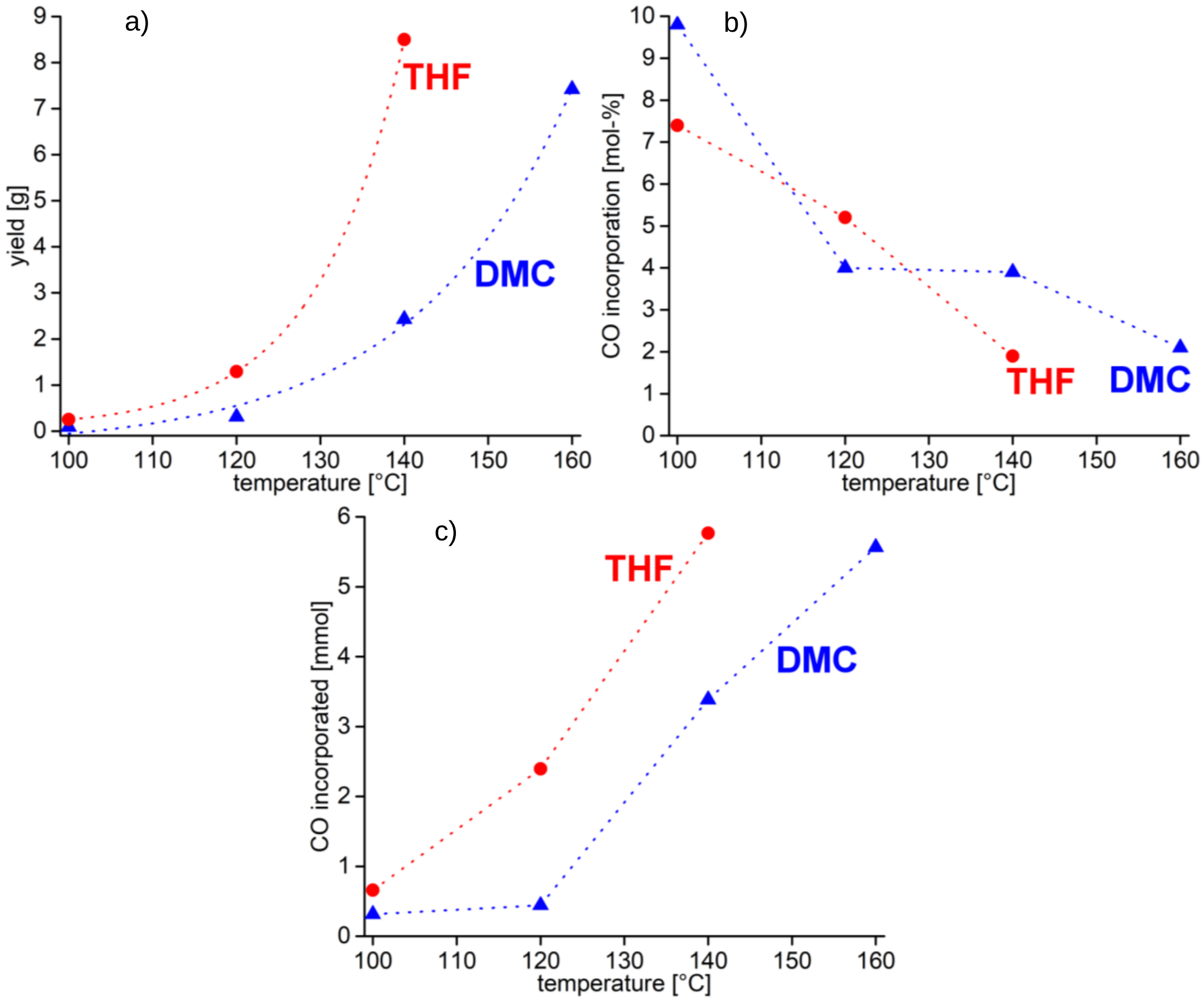

Supplementary Figure 43: Influence of temperature. Effect of *T* on the polymer yield (a) and the relative (b) and total CO incorporation (c) in different media. Reaction conditions: 1.5 bar CO and 58.5 bar ethylene at 20°C, 300-330 bar total pressure, 1 h, 75 mL of organic solvent (THF: red circles, DMC: blue triangles), 0.3 mmol di-*tert*-butyl peroxide, 1000 rpm stirring rate with a pitched blade stirrer. Dashed lines are merely a guide to the eye.

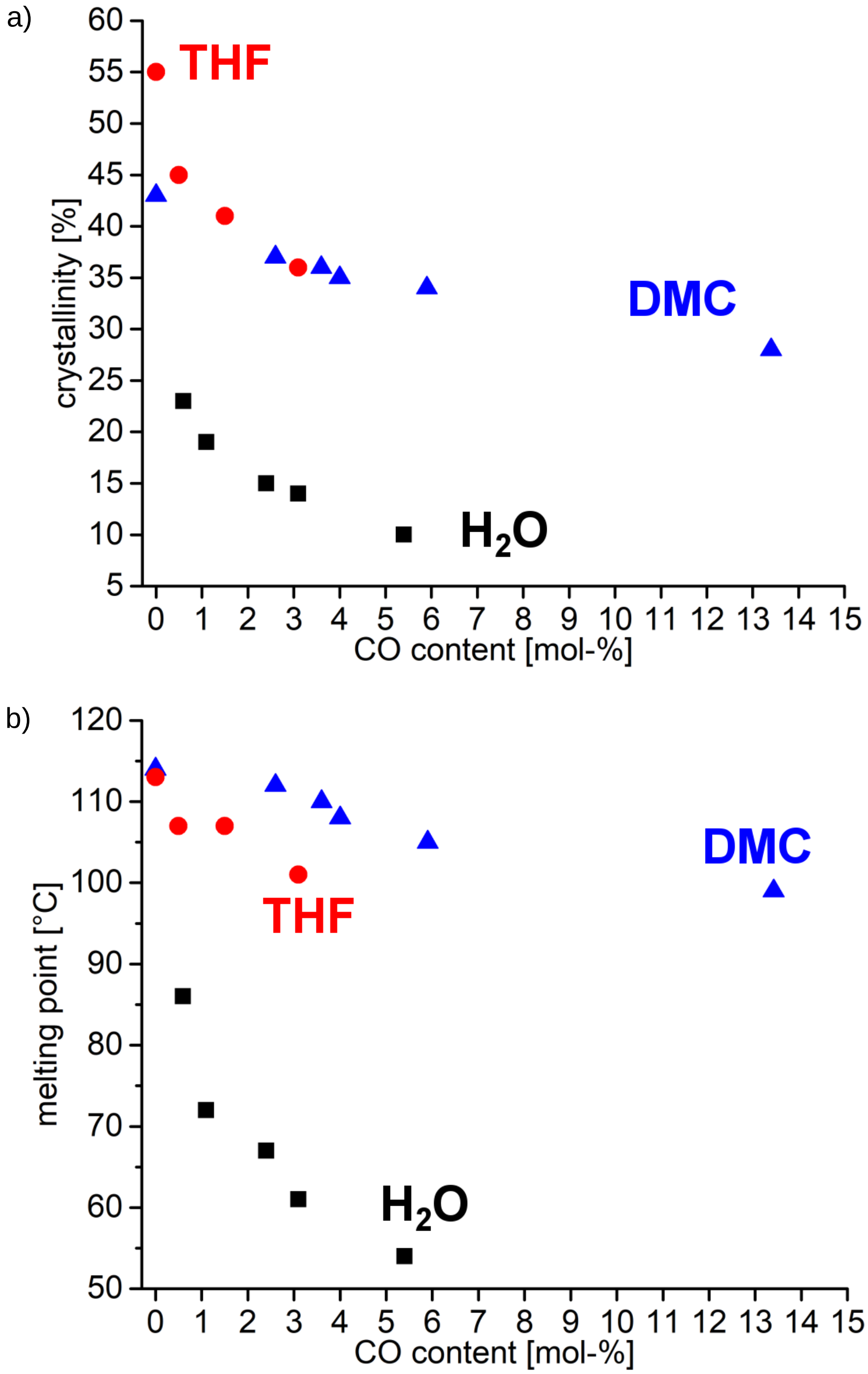

Supplementary Figure 44: Correlation of the copolymer CO content and its thermal properties. Polymerizations were carried out under the following reaction conditions: 120°C, 260-330 bar total pressure, 1 h, 150 mL of water (black squares) or 75 mL of the respective organic solvent (THF: red circles, DMC: blue triangles), 0.3 mmol free-radical initiator (VA-086 in water, di-*tert*-butyl peroxide in organic solvents), 1000 rpm stirring rate with a pitched blade stirrer. The branching density was only little affected by the CO concentration, but depends mostly on the reaction medium. Branches per 1000 carbon backbone atoms by ^{13}C NMR: ~34 for copolymers from water, ~10 for copolymers from THF and ~13 for copolymers from DMC.

Film preparation and characterization

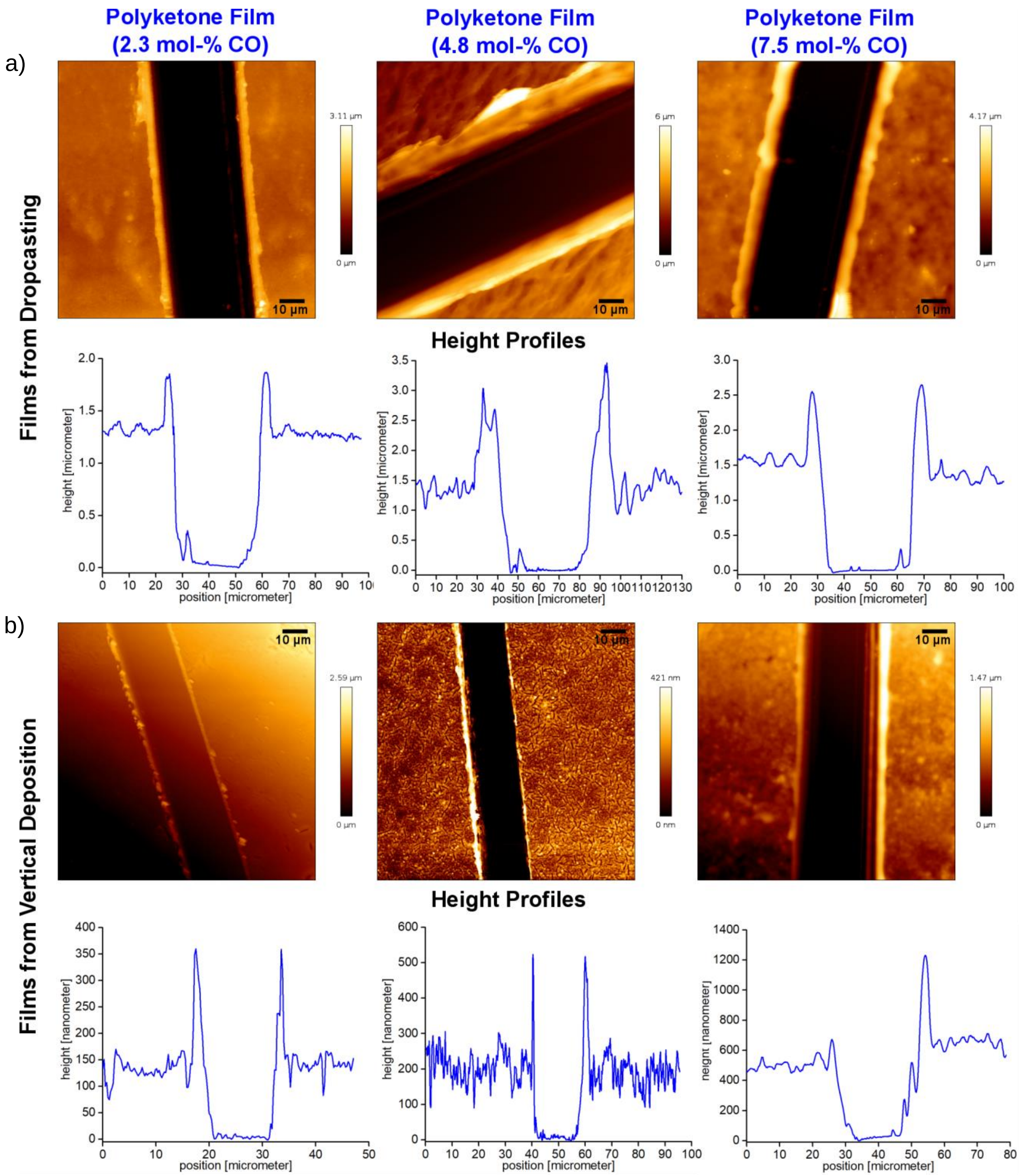

Supplementary Figure 45: Film analysis. AFM images of polyketone films with different CO contents deposited from aqueous polyketone dispersions by dropcasting (a) or vertical deposition (b) on silicon substrates. The measured height profiles over cuts, introduced for this purpose, were used for determination of the film thickness and the films' surface roughness.

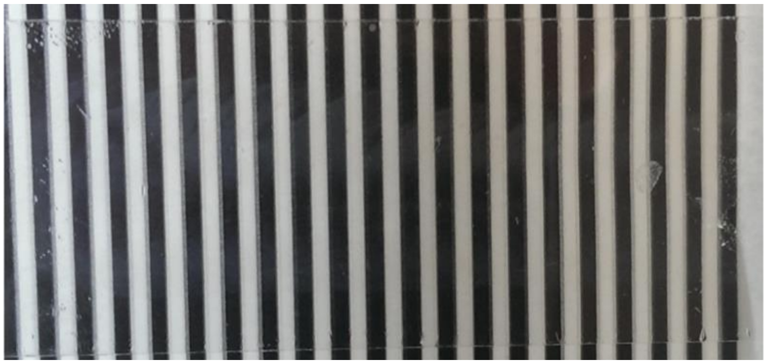

Supplementary Figure 46: Optical appearance of an exemplary polyketone film (0.5 mol-% CO content, M_n = 22 000 g×mol^{-1}, 3 μm film thickness) on a glass substrate in front of a striped background.

Film decomposition under UV-irradiation

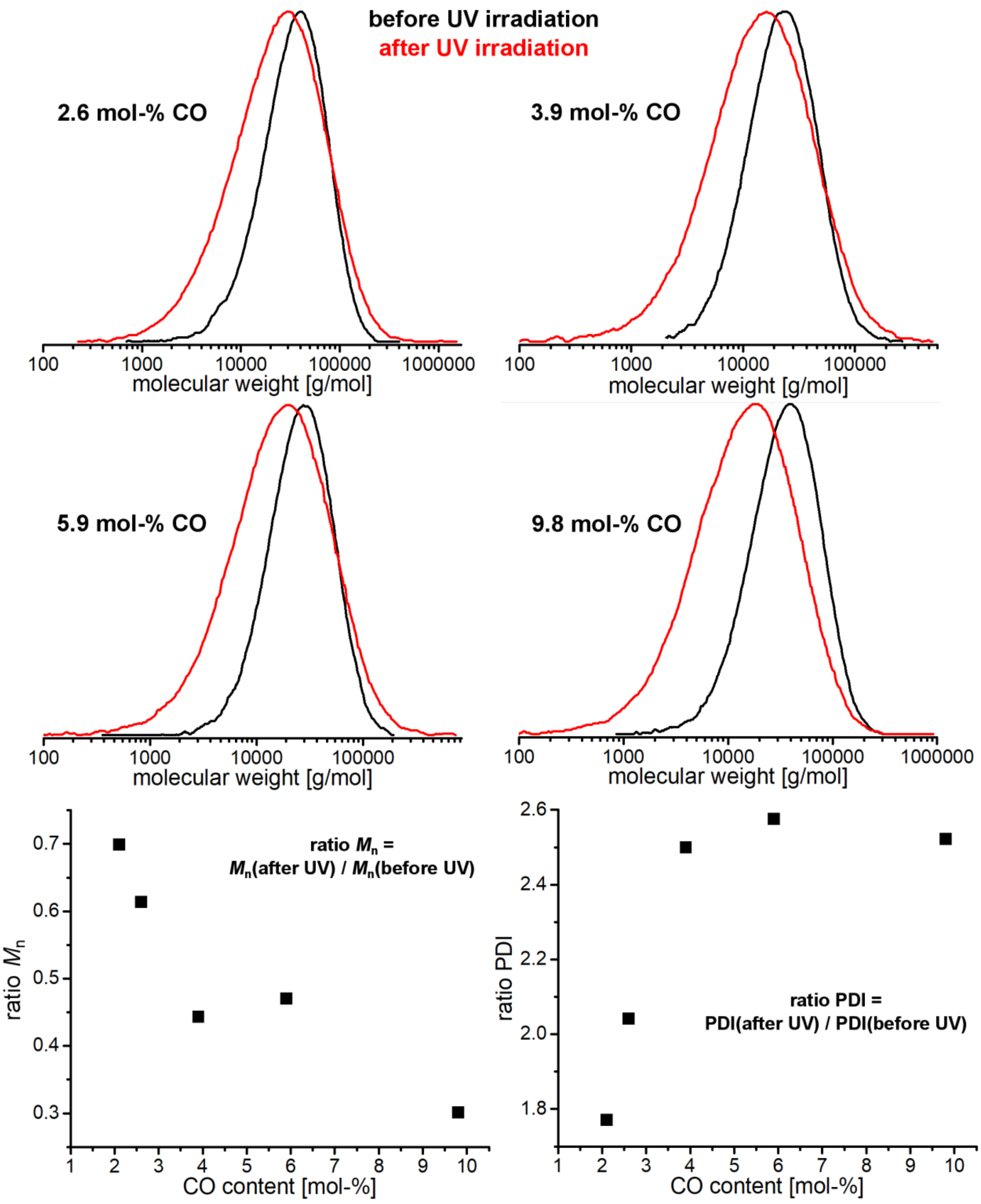

Supplementary Figure 47: UV irradiation of polyketone films. Changes in molecular weight distributions of films with different CO-contents after 6 h of irradiation with UV light (350–400 nm, 4 × 8W tubes). Films were prepared from toluene solutions of polyketones synthesized in DMC (entries 2, 4, 6, 8 and 10 in Table 1).

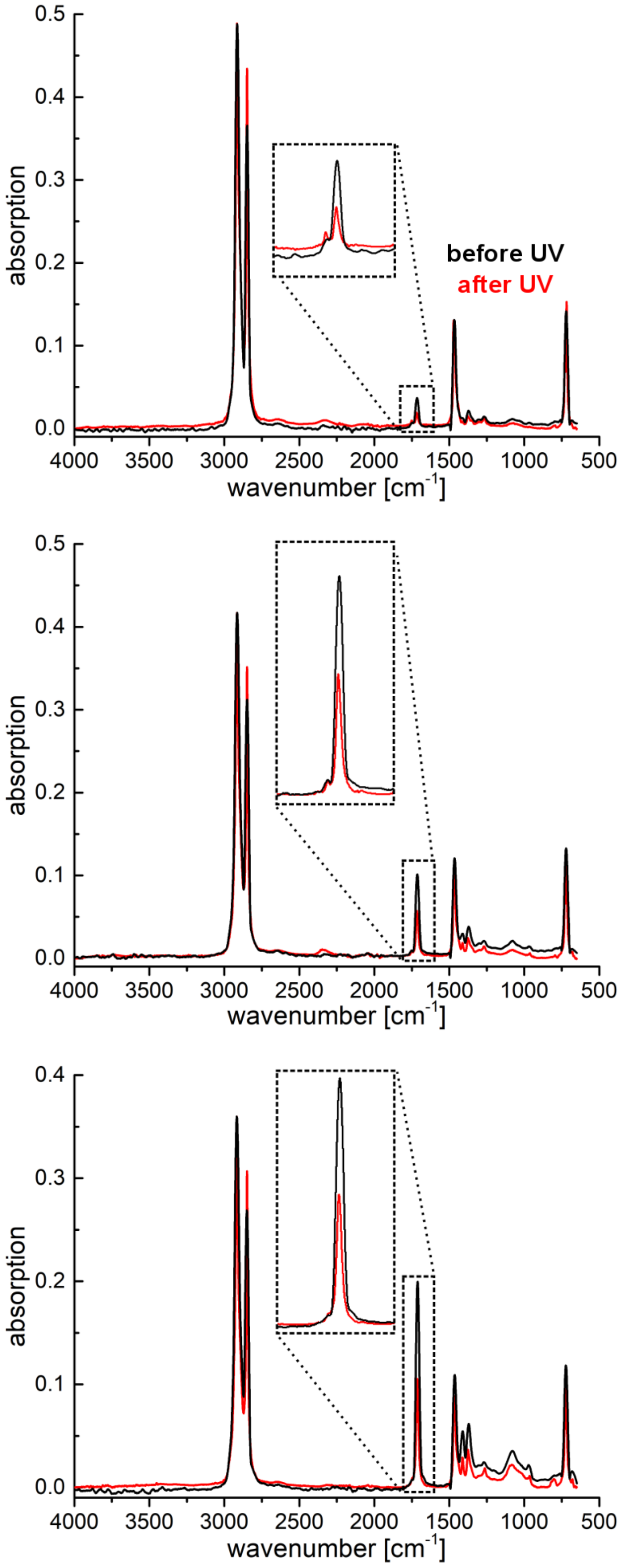

Supplementary Figure 48: UV irradiation of polyketone films. ATR-IR spectra of different polyketone films related to Supplementary Figure 47 before and after UV irradiation (6 h, 350–400 nm, 4 × 8W tubes). The carbonyl content decreased upon irradiation indicating Norrish type reactions.

Supplementary Tables

Monomer reactivity ratio for free-radical CO-ethylene-copolymerization

Supplementary Table 1. Concentrations of ethylene and carbon monoxide in the reaction mixtures of entries 4, 6, 8, 9 and 10 of Table 1, compositions of the obtained polyketones and calculated reactivity ratios $r_E = k_e/k_x$.

# (Tab. 1)	*p*(CO) at 20°C [bar]	*T* [°C]	$[ethylene]_0$ [mol×L^{-1}]	$[CO]_0$ [mol×L^{-1}]	CO convers. [%]	$[ethylene]_{poly}$ [%]	$[CO]_{poly}$ [%]	r_E
4	1.0	120	9.3	0.045	4	96.0	4.0	0.11
6	1.5	120	9.3	0.068	5	94.1	5.9	0.11
8	1.5	100	9.9[a]	0.068	2	90.2	9.8	0.05
9	1.5	140	8.7	0.068	19	96.1	3.9	0.18
10	1.5	160	8.1[a]	0.068	30	97.9	2.1	0.38

a) Value from linear extrapolation from measured solubilities at 120 and 140°C.

Monomer reactivity ratio for catalytic CO-ethylene-copolymerization

Supplementary Table 2. Selected data of Luo *et al.* for the calculation of relative monomer reactivity of Pd-catalyzed copolymerization of ethylene and carbon monoxide.[13]

# (from Tab. 1 in [13])	*T* [°C]	CO pressure [bar]	ethylene pressure [bar]	CO convers. [%]	$[CO]_{poly}$ [%]
2	90	1.0	20.7	23	6
5	110	1.4	20.7	38	8
7	110	1.7	20.7	37	9

Catalyst: $Pd(OAc)_2$ + P^SO_3H, in dichloromethane.

Supplementary Table 3. Estimated concentrations of ethylene and carbon monoxide in the reaction mixtures of Supplementary Table 2,[13] compositions of the obtained polyketones and calculated reactivity ratio $r_E = k_e/k_X$.

# (from Tab. 1 in [13])	CO pressure [bar]	T [°C]	[ethylene]$_0$ [mol×L^{-1}]	[CO]$_0$ [mol×L^{-1}]	CO convers. [%]	[ethylene]$_{poly}$ [%]	[CO]$_{poly}$ [%]	r_E
2	1.0	90	1.53	0.0138	23	94	6	0.13
5	1.4	110	1.53	0.0193	38	92	8	0.13
7	1.7	110	1.53	0.0235	37	91	9	0.14

Synthesis and characterization of terpolymers of E, CO and difunctional monomer

Supplementary Table 4. Terpolymerizations of ethylene, CO and ethylene glycol dimethacrylate (EGDMA, entries 2-6) or divinyl adipate (DVA, entries 7-11) as difunctional monomers (DM).[a]

#	difunc. mon. (DM)	DM conc. [mM]	yield [g]	peak T_m	cryst. [%][b]	M_w[c] [10^3 g×mol^{-1}]	M_w/M_n[c]	χ(CO)[d] [mol-%]	χ(DM)[d] [mol-%]
1	-	-	7.42	103	34	10.2	1.7	2.1	-
2	EGDMA	9	7.98	102	32	10.1	1.9	1.7 (1.5)	0.3 (0.25)
3	EGDMA	18	7.36	104	32	9.9	1.9	1.6 (1.3)	0.5 (0.30)
4	EGDMA	38	8.13	102	36	11.8	1.8	1.9	1.2
5	EGDMA	75	7.30	101	28	12.3	2.0	2.3 (1.4)	1.6 (1.3)
6	EGDMA	113	6.62	100	27	20.7	2.2	2.3 (1.8)	2.0 (2.5)
7	DVA	20	7.20	102	38	n.a.	n.a.	1.8	0.2
8	DVA	39	8.50	101	38	14.2	2.2	1.5 (1.1)	0.3 (0.20)
9	DVA	75	9.67	99	37	25.9	3.9	1.5 (1.1)	0.4 (0.35)
10	DVA	100	9.42	98	34	90.0	11.8	1.5	0.9
11	DVA	151	9.63	99	35	-[e]	-[e]	1.8	1.7

[a]Reaction conditions: 1 h reaction time, 75 mL of DMC, 4 mM di-*tert*-butyl peroxide, 1.5 bar CO pressure at 20°C, initial ethylene pressure 310-320 bar, 160°C reaction temperature, 1000 rpm stirring rate (pitched blade). [b]Determined by 2nd heating cycle of DSC on the isolated bulk polymer. [c]Determined by GPC at 160°C calibrated with linear PE. [d]CO or difunctional monomer (DM) incorporation from ATR-IR (calibrated with a polyketone reference). In brackets: according to ^{13}C NMR. [e]Sample is meltable but not completely soluble in di- or trichlorinated benzenes at 160°C.

Specimen preparation and stress-strain tests

Supplementary Table 5. Mechanical properties of polyketones (entries 1 and 2) and terpolymers of ethylene, carbon monoxide and either EGDMA (3-7) or DVA (8-11).[a]

#	difunc. mon. (DM)	χ(DM)[b] [mol-%]	M_w[c] [10^3 g×mol^{-1}]	cryst. [%][d]	E_t [MPa]	ε_{tb} [%]	ε_{max} [%]	σ_b [MPa]	σ_{max} [MPa]
1	-	-	12.3	35 (32)	250	10	9	3.2	8.7
2	-	-	10.2	45 (34)	347	15	13	2.5	6.9
3	EGDMA	0.3	10.1	- (32)	160	18	13	2.5	6.7
4	EGDMA	0.5	9.9	- (32)	165	24	15	2.4	6.9
5	EGDMA	1.2	11.8	38 (36)	158	28	25	2.3	6.3
6	EGDMA	1.6 (1.3)	12.3	22 (28)	126	23	20	2.1	5.8
7	EGDMA	2.0 (2.5)	20.7	20 (27)	149	15	14	2.6	6.8
8	DVA	0.2	n.a.	38 (38)	134	34	32	2.5	6.7
9	DVA	0.3	14.2	41 (38)	167	50	40	2.5	7.0
10	DVA	0.4	25.9	41 (37)	325	41	37	2.4	6.3
11	DVA	0.9	90.0	39 (34)	186	35	34	3.1	8.2

[a]Tensile testing conditions according to ISO 527-2, type 5A (injection molded, dogbone-shaped specimens with 75 × 12.5 × 2 mm^3, 5 mm min^{-1} crosshead speed). [b]Difunctional Monomer (DM) incorporation from ATR-IR (calibrated with a polyketone reference). In brackets: according to ^{13}C NMR. [c]Determined by GPC at 160°C calibrated with linear PE. [d]From 1st (2nd) heating cycle of DSC on the isolated bulk polymer.

Film preparation and characterization

Supplementary Table 6. Results of AFM analysis of films with different polymer contents deposited from aqueous polyketone dispersions by dropcasting (DC) or vertical deposition (VD).

#	CO content [mol-%]	dispersion's polymer content [wt.-%]	deposition method	average film thickness [nm]	arithmetic average roughness [nm]	root mean square roughness [nm]
**1**	0.5	0.5	DC	3800	110	133
		2.2	VD	140	15	21
**2**	2.3	0.3	DC	1300	29	37
		0.9	VD	130	15	20
**3**	4.8	0.3	DC	1350	92	116
		0.6	VD	190	34	43
**4**	7.5	0.3	DC	1460	53	64
		0.6	VD	550	25	32

Surface roughness parameters are defined as follows and were determined over 20-30 µm (100 to 120 image data points):

1) Arithmetic average roughness (AAR):

$$\mathrm{AAR} = \frac{1}{n}\sum_{i=1}^{n}\left|h_i - \bar{h}\right| \qquad \text{(Supplementary Equation 2)}$$

2) Root mean square roughness (RMSR):

$$\mathrm{RMSR} = \sqrt{\frac{1}{n}\sum_{i=1}^{n}\left(h_i - \bar{h}\right)^2} \qquad \text{(Supplementary Equation 3)}$$

with number of data points n, height h_i of the film at position i and average height $\bar{h}$.

Supplementary References

1. Ortmann, P., Wimmer, F. P., Mecking, S. Long-Spaced Polyketones from ADMET Copolymerizations as Ideal Models for Ethylene/CO Copolymers. *ACS Macro Lett.* **4**, 704-707 (2015).
2. Galland, G. B., de Souza, R. F, Mauler, R. S., Nunes, F. F. ^{13}C NMR Determination of the Composition of Linear Low-Density Polyethylene Obtained with [η^3-Methallyl-nickel-diimine]PF_6 Complex. *Macromolecules* **32**, 1620-1625 (1999).
3. Grau, E., Broyer, J.-P., Boisson, C., Spitz, R., Monteil, V. Free Radical Ethylene Polymerization under Mild Conditions: The Impact of the Solvent. *Macromolecules* **42**, 7279-7281 (2009).
4. Grau, E., Broyer, J.-P., Boisson, C., Spitz, R., Monteil, V. Unusual activation by solvent of the ethylene free radical polymerization. *Polym. Chem.* **2**, 2328-2333 (2011).
5. Luo, R., Newsham, D. K., Sen, A. Palladium-Catalyzed Nonalternating Copolymerization of Ethene and Carbon Monoxide: Scope and Mechanism. *Organometallics* **28**, 6994-7000 (2009).
6. Soomro, S. S., Cozzula, D., Leitner, W., Vogt, H., Müller, T. E. The microstructure and melt properties of CO-ethylene copolymers with remarkably low CO content. *Polym. Chem.* **5**, 3831-3837 (2014).
7. Sychev, V. V., Vasserman, A. A., Golovsky, E. A., Kozlov, A. D., Spiridonov, G. A., Tsymarny, V. A. *Thermodynamic Properties of Ethylene* (*National Standard Reference Data Service of the USSR)*, 1st ed., Selover, T. B. Jr., Ed., Springer, 1987, Vol. 7.
8. Grau, E., Broyer, J.-P., Boisson, C., Spitz, R., Monteil, V. Supercritical behavior in free radical polymerization of ethylene in the medium pressure range. *Phys. Chem. Chem. Phys.* **12**, 11665-11669 (2010).
9. Hayduk, W., Battino, R., Clever, H. L., Derrick, M. E., Fogg, P. G. T. *IUPAC, Analytical Chemistry Division Commission on Solubility Data. Solubility Data Series – Ethene*; Lorimer, J. W., Fogg, P. T., Young, C. L., Gujral, P. D., Eds., Oxford University Press, 1994, Vol. 57.
10. Cargill, R. W., Battino, R., Bo, S., Clever, H. L., Gjaldbaek, J. C., Wiesenburg, D. A., Wilhelm, E., Yampol'skii, Y. P., Young, C. L. *IUPAC, Analytical Chemistry Division*

Commission on Solubility Data. Solubility Data Series – Carbon Monoxide; Lorimer, J. W., Gujral, P. D., Eds., Pergamon Press, 1990, Vol. 43.

11. Naejus, R., Lemordant, D., Coudert, R., Willmann, P. Excess thermodynamic properties of binary mixtures containing linear or cyclic carbonates as solvents at the temperatures 298.15 and 313.15 K. *J. Chem. Thermodyn.* **29**, 1503-1515 (1997).
12. Thiebaut, J., Rivail, J., Greffe, J. Dielectric studies of non electrolyte solutions. Part 3.-Conformational equilibria in 1,2-dichloroethane and dimethyl carbonate. *J. Chem. Soc., Faraday Trans. 2* **72**, 2024-2034 (1976).
13. Luo, R., Newsham, D. K., Sen, A. Palladium-Catalyzed Nonalternating Copolymerization of Ethene and Carbon Monoxide: Scope and Mechanism. *Organometallics* **28**, 6994-7000 (2009).
14. Chorazewski, M., Troncoso, J., Jacquemin, J. Thermodynamic Properties of Dichloromethane, Bromochloromethane, and Dibromomethane under Elevated Pressure: Experimental Results and SAFT-VR Mie Predictions. *Ind. Eng. Chem. Res.* **54**, 720-730 (2015).
